# Five new species of *Inosperma* from China: Morphological characteristics, phylogenetic analyses, and toxin detection

**DOI:** 10.3389/fmicb.2022.1021583

**Published:** 2022-10-31

**Authors:** Sai-Nan Li, Fei Xu, Pan Long, Feng Liu, Ping Zhang, Yu-Guang Fan, Zuo-Hong Chen

**Affiliations:** ^1^Mycology Laboratory, College of Life Sciences, Hunan Normal University, Changsha, China; ^2^Department of Physical and Chemical, Ningxia Hui Autonomous Region Center for Disease Control and Prevention, Yinchuan, China; ^3^Key Laboratory of Tropical Translational Medicine of Ministry of Education, Hainan Key Laboratory for R&D of Tropical Herbs, School of Pharmacy, Hainan Medical University, Haikou, China

**Keywords:** Inocybaceae, new species, phylogeny, taxonomy, muscarine, psilocybin, ibotenic acid, muscimol

## Abstract

Many species of *Inosperma* cause neurotoxic poisoning in humans after consumption around the world. However, the toxic species of *Inosperma* and its toxin content remain unclear. In the present study, we proposed five new *Inosperma* species from China, namely, *I. longisporum, I. nivalellum*, *I. sphaerobulbosum*, *I. squamulosobrunneum*, and *I. squamulosohinnuleum*. Morphological and molecular phylogenetic analyses based on three genes (ITS, nrLSU, *rpb2*) revealed that these taxa are independent species. A key to 17 species of *Inosperma* in China is provided. In addition, targeted screening for the most notorious mushroom neurotoxins, muscarine, psilocybin, ibotenic acid, and muscimol, in these five new species was performed by using ultrahigh-performance liquid chromatography-tandem mass spectrometry (UPLC-MS/MS). Our results show that the neurotoxin contents in these five species varied: *I. sphaerobulbosum* contains none of the tested neurotoxins; *I. nivalellum* is muscarine positive; *I. longisporum* and *I. squamulosohinnuleum* contain both ibotenic acid and muscimol, and *I. squamulosobrunneum* only contains muscimol; psilocybin was not detected in these five new species.

## Introduction

Macrofungi have important ecological and economical values. The species diversity, taxonomy, and phylogeny of macrofungi have been extensively investigated in recent years, and many new species have been discovered (Han et al., 2016; Cui et al., 2019; Shen et al., 2019; Sun et al., 2020, 2022; Cao et al., 2021; Liu et al., 2021a,b, 2022a,2022b; Zhang et al., 2021; Ji et al., 2022; Song et al., 2022; Wang et al., 2022). The genus *Inosperma* (Kühner) Matheny and Esteve-Rav. (type: *I. calamistratum* [Fr.] Matheny and Esteve-Rav. = *Inocybe calamistrata* [Fr.] Gillet) is a group of macrofungi which was first conceived as a subgenus of *Inocybe* (Fr.) Fr. by Kühner (1980), and recent studies elevated it to the genus rank (Matheny et al., 2019). Currently, 12 *Inosperma* species have been reported from northeast, northwest, southwest, and southernmost China, five of which are new species that were recently discovered (Fan and Bau, 2018; Deng et al., 2021a,b, 2022). Members of this genus are characterized by small- to medium-sized basidiomata, rimose to scaly pileus, even or bulbous stipe base, rubescent, or brunnescent context, often phaseoliform basidiospores, hyaline, or necropigmented basidia, thin-walled cheilocystidia, lack of pleurocystidia, and often have distinctive odors (Matheny et al., 2019; Deng et al., 2021a). The genus occurs on the ground, associates with vascular plants in mycorrhizal symbiosis, and is composed of more than 70 known taxa that are distributed in Africa, Asia, Australasia, Europe, and North America (Matheny et al., 2019; Deng et al., 2021a). Multigene (ITS, nrLSU, *rpb2*, and mtSSU) molecular studies have confirmed that the genus is monophyletic and is classified into six major lineages, the *Cervicolores* clade, Maculatum clade, *I. misakaense* lineage, *I. africanum* lineage, and two Old World tropical clades (Larsson et al., 2009; Pradeep et al., 2016; Matheny et al., 2019; Deng et al., 2022).

Foraging for and consuming wild *Inosperma* mushrooms has led to an increased incidence of neurotoxic mushroom poisoning, the main symptoms of which include sweating, salivation, and lachrymation (Lurie et al., 2009; White et al., 2018). As early as 1963, *I. erubescens* (A. Blytt) Matheny and Esteve-Rav. led to mass poisonings in humans in Germany (Herrmann, 1964; Lurie et al., 2009). In recent years, Bijeesh et al. (2020) reported two cases of poisoning by *I. carnosibulbosum* (C. K. Pradeep and Matheny) Matheny and Esteve-Rav. from southwestern India. Parnmen et al. (2021) reported 10 cases of poisoning caused by *Inosperma* species in Thailand from 2010 to 2018. In China, *I.* aff. *virosum* (K. B. Vrinda, C. K. Pradeep, A. V. Joseph, and T. K. Abraham ex C. K. Pradeep, K. B. Vrinda and Matheny) Matheny and Esteve-Rav. and *I.* cf. *virosum*, which stimulate the parasympathetic nervous system, are two species that were newly discovered as poisonous mushrooms (Li et al., 2020). *Inosperma muscarium*, a new species in China, was responsible for two poisoning incidents involving seven people (Deng et al., 2021a). In the latest report, a new poisonous *Inosperma* species, *I. zonativeliferum* caused poisoning in 10 people (Deng et al., 2022).

Muscarine, psilocybin, ibotenic acid, and muscimol are the four main mushroom neurotoxins (Chen et al., 2016). Muscarine is the principal toxin in *Inosperma* which binds to acetylcholine receptors and induces classic parasympathetic stimulation that includes profuse sweating, lacrimation, and salivation (Diaz, 2005; White et al., 2018). Another toxin that occurs in *Inosperma* mushrooms is psilocybin. As a tryptamine alkaloid, psilocybin acts on serotonin 5-HT2A/C receptor sites and induces central nervous system (CNS) effects, illusions, hallucinations, altered sense of time, space synesthesia, and feelings of euphoria (Griffiths et al., 2006; White et al., 2018). Based on a review of the literature and their own work on toxin detection, Kosentka et al. (2013) outlined 99 muscarine- and psilocybin-containing species of *Inocybe s.l.* from 1960 to 2013, including 13 species of *Inosperma*. Of these, there was a lack of psilocybin-positive species, and only three species [*I. erubescens*, *I. maculatum* (Boud.) Matheny and Esteve-Rav., and *I. vinaceobrunneum* (Matheny, Ovrebo and Kudzma) Haelew.] were muscarine-positive. Gartz (1986) suggests the presence of psilocybin in *I. calamistratum*. However, it has since been demonstrated to be psilocybin negative (Stijve and Kuyper, 1988). Recently, Latha et al. (2020) first reported the occurrence of muscarine in *I. virosum*, and Deng et al. proposed three new *Inosperma* species from tropical China, namely, *I. muscarium*, *I. hainanense* and *I. zonativeliferum*, all of which are muscarine-positive (Deng et al., 2021a,^2022^). In addition, *I. carnosibulbosum* is probably a muscarine-positive species due to a recent report of poisonous cases (Bijeesh et al., 2020).

Other frequently occurring mushroom neurotoxins are ibotenic acid and muscimol, which are the main isoxazole derivatives. Ibotenic acid and muscimol resemble and act as two main neurotransmitters of the central nervous system, namely, glutamic acid and γ-aminobutyric acid, and cause neuroexcitatory effects, such as drowsiness and manic excitement (White et al., 2018). Several analytical strategies have been reported for the identification of ibotenic acid and muscimol, such as high-performance liquid chromatography (HPLC) (Fuchs et al., 2011; Plenert et al., 2012; Brandenburg and Ward, 2018), liquid chromatography-mass spectrometry (LC-MS) (Stebelska, 2013), liquid chromatography-tandem mass spectrometry (LC-MS-MS) (Govorushko et al., 2019), and gas chromatography-mass spectrometry (GC-MS) (Audi et al., 2005; Verougstraete et al., 2018). In Europe and North America, the majority of reported cases of isoxazole derivative mushroom poisoning are caused by *Amanita muscaria* (L.) Lam. and *A. pantherina* (DC.) Krombh. In addition, *A. gemmata* (Fr.) Bertill., *A. cothurnata* G. F. Atk., *A. cokeri* E.-J. Gilbert and Kühner ex E.-J. Gilbert, *A. frostiana* (Peck) Sacc., *A. ibotengutak* T. Oda, C. Tanaka and Tsuda and *A. strobiliformis* (Paulet ex Vittad.) Bertill. also contain isoxazole derivatives (Spoerke and Rumack, 1994; Benjamin, 1995; Chen et al., 2016). In southern Vietnam, Doan et al. (2017) confirmed the presence of ibotenic acid in *Paraisaria heteropoda* (Kobayasi) Luangsa-ard, Mongkols. and Samson. To date, no isoxazole derivative detection in *Inosperma* has been reported worldwide. In this study, the contents of ibotenic acid and muscimol in five new species of *Inosperma* were determined by UPLC/MS-MS, which is the first time that ibotenic acid and muscimol have been detected in species of *Inosperma*.

In this study, (1) we describe five new species of *Inosperma* based on morphological and multigene phylogenetic evidence, and a key to 17 species of *Inosperma* in China was provided; (2) the presence of ibotenic acid and muscimol in *Inosperma* species was identified for the first time; and (3) we determined the muscarine and psilocybin contents to provide accurate data for the prevention and clinical treatment of potential *Inosperma* poisoning accidents.

## Materials and methods

### Specimen collection and drying treatment

Specimens were opportunistically collected from Hubei, Yunnan, and Sichuan Provinces. The fresh basidiomata were dried using an EVERMAT electric dryer operated at 45°C for 10 h. The dried specimens were deposited in the Mycological Herbarium of Hunan Normal University (MHHNU), Changsha, China. A small piece of fresh basidioma was also dried with silica gel for molecular analysis for each specimen.

### Morphological studies

Specimens were photographed *in situ* using a Sony digital camera (LICE-7, Sony, Tokyo, Japan). The macromorphological characteristics of fresh mushrooms were recorded as soon as possible after collection. Color codes were described following Kornerup and Wanscher (1978). Microscopic structures were studied from dried materials mounted in 5% aqueous KOH, and 1% Congo red resolution was used as a stain when necessary. All measurements were performed at 1000 × magnification, and a minimum of 20 basidiospores from each basidioma were measured in the side view. Micromorphological investigations were performed by means of a Nikon Eclipse 50i microscope (Nikon, Tokyo, Japan). The dimensions of basidiospores and *Q* values are given as (a) b–c (d), where “b–c” cover a minimum of 90% of the measured values, and “a” and “d” represent extreme values. *Q* is the ratio of the length to width of an individual basidiospore and *Q*_*m*_ is the average *Q* of all basidiospores ± sample standard deviation. The abbreviation *n* is the number of basidiospores, coll. means collections, av. means average, and SD means standard deviation. The descriptive terms are in accordance with Matheny and Bougher (2017) and Deng et al. (2021a).

### DNA extraction, amplification, and sequencing

DNA was extracted from dried basidiomata using a fungal DNA extraction kit manufactured by Omega Bio-Tek (Norcross, GA, USA). The following primer pairs were used for PCR amplification and sequencing: ITS5 and ITS4 for the internal transcribed spacer (ITS) region (White et al., 1990); LR0R and LR7 for the nuclear ribosomal large subunit (nrLSU) region (Vilgalys and Hester, 1990); and bRPB2-6F and bRPB2-7.1R for the RNA polymerase II second largest subunit (*rpb2*) region (Matheny, 2005). The PCR protocols for ITS and nrLSU were as described in White et al. (1990), and those for *rpb2* were as described in Matheny (2005). The PCR products were purified and sequenced by Tsingke Biological Technology Co., Ltd. (Beijing, China).

### Sequence alignment and phylogenetic analyses

Thirty-nine sequences (13 for ITS, 13 for nrLSU, and 13 for rpb2) were newly generated in this study and were deposited in GenBank (Supplementary Table 1). Additional sequences were retrieved from GenBank^1^ and previously published articles (Supplementary Table 1; Pradeep et al., 2016; Latha and Manimohan, 2017; Matheny and Bougher, 2017; Fan and Bau, 2018; Matheny et al., 2019; Aïgnon et al., 2021; Bandini et al., 2021; Deng et al., 2021a,b, 2022).

The sequences were aligned using MAFFT v7.310 (Katoh and Standley, 2013) and manually edited using BioEdit v7.0.5 (Hall, 1999). Maximum likelihood (ML) analysis was performed using the W-IQ-TREE web service^2^ with 1,000 ultrafast bootstrap replicates (Trifinopoulos et al., 2016). Bayesian inference (BI) was performed in MrBayes v3.2 (Ronquist et al., 2012). The optimal substitution model was determined using the Akaike information criterion (AIC) as implemented in MrModeltest v2.3 (Nylander, 2004). The phylograms from ML and BI analyses were visualized with FigTree v1.4.3 (Rambaut, 2009).

### Analysis of toxins by ultrahigh-performance liquid chromatography-tandem mass spectrometry

Dried basidiomata of the target taxon were used for toxin analyses using the methods of Xu et al. (2020) and Deng et al. (2022) with slight modifications. The dried mushroom pileus (0.05 g) was crushed into a fine powder and mixed with 2 mL of methanol–water solution (7:3 v/v). The mixture was vortexed for 30 min at room temperature and treated in an ultrasonic bath for 30 min. After centrifugation at 10,000 rpm for 5 min, the supernatant was purified using a QuEChERS-PP column. Subsequently, the extract was mixed with acetonitrile to a final volume of 1.0 mL. The obtained sample solution was centrifuged at 21,000 rpm for 2 min before ultrahigh-performance liquid chromatography-tandem mass spectrometry (UPLC-MS/MS) analysis. *Lentinula edodes* was used as a blank sample.

The presence of neurotoxins, especially muscarine, psilocybin, ibotenic acid, and muscimol (Alta Scientific Co., Ltd., Tianjin, China), was evaluated through UPLC–MS/MS, which was carried out with a Waters ACQUITY I-Class UPLC system coupled with a Waters Xevo TQ-S MS/MS system (Waters, Milford, MA, USA) under the conditions shown in Table 1. The analytical results were reported as X ± U (*k* = 2, *p* = 95%), where *X* is the analytical content and *U* is the expanded measurement uncertainty.

**TABLE 1:** Instrument parameters for the UPLC-MS/MS analyses.

Compounds	Q1 Mass (Da)	Q3 Mass (Da)	DP (V)	CE (V)	Chromatographic condition	RT (min)
Muscarine	174.2	57.1^a^, 97.2^b^	73, 70	32, 26	Mobile phase solvent: 10 mmol/L ammonium acetate – 0.05% formic acid aqueous solution (A), acetonitrile (B) Gradient elution: 0.1–2.0 min, 5% B; 2.0–3.5 min, 5–80% B; 3.5–4.5 min, 80% B; 4.5–5.0 min, 80–5% B, 5.0–7.0 min, 5% B	1.30
Ibotenic acid	158.9	113.2^a^, 142.1^b^	50, 50	16, 17	Mobile phase solvent: 0.2% formic acid aqueous solution (A), acetonitrile (B)	0.88
Muscimol	115.1	98.0^a^, 68.1^b^	43, 40	17, 20	Gradient elution: 0.1–2.0 min, 2% B; 2.0–3.5 min, 2–80% B; 3.5–4.5 min, 80% B; 4.5–5.0 min, 80–2% B, 5.0–7.0 min, 2% B	0.88
Psilocybin	285.2	205.3^a^, 58.1^b^	62, 62	26, 53	Mobile phase solvent: 10 mmol/L ammonium acetate aqueous solution (A), acetonitrile (B) Gradient elution: 0.5–3.5 min, 5–90% B; 3.5–5.0 min, 90% B; 5.0–7.0 min, 90–5% B; 7.0–10.0 min, 5%B	1.31

DP, declustering potential; CE, collision energy; RT, retention time. a, quantitative ion; b, qualitative ion.

## Results

### Phylogenetic data

**FIGURE 1:**
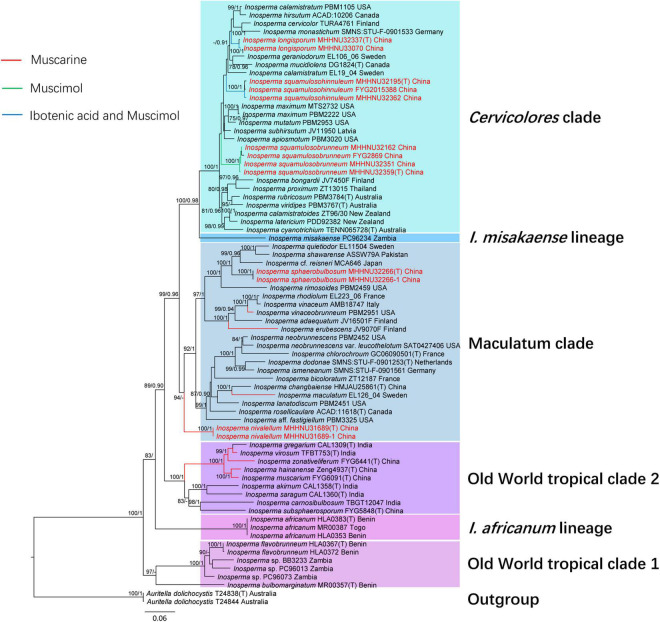
Phylogenetic relationship and placement of *Inosperma* inferred from the combined dataset (ITS, nrLSU, and rpb2) using ML and BI phylogenetic methods. Bootstrap values (MLB) ≥ 70% and Bayesian posterior probabilities (BPP) ≥ 0.90 are reported at relevant nodes (ML/BI). The new species are indicated in red. Only the ML tree is shown. Toxins refer to Kosentka et al. (2013), Bijeesh et al. (2020), and Deng et al. (2021a,2022).

The combined dataset (ITS, nrLSU, and *rpb2*) included 73 sequences of 56 taxa and contained 3,100 total characters with 882 bp ITS, 1,566 bp nrLSU, and 652 bp *rpb2*. An additional dataset file shows this in more detail (Supplementary Data Sheet 1). The best-fit models for the combined dataset selected by MrModeltest v2.3 is General Time Reversible + Proportion–Invariant + Gamma (GTR + I + G). The topologies of the ML (Supplementary Figure 1) and BI (Supplementary Figure 2) phylogenetic trees obtained in this study are practically the same, and only the ML tree with branch lengths and support values is shown in Figure 1. All members of *Inosperma* in the dataset formed a monophyletic lineage with strong support (MLB = 100%, BPP = 1.0). Phylogenetically, the *Cervicolores* clade is retrieved with strong support (MLB = 100%, BPP = 1.0), and *I. longisporum*, *I. squamulosobrunneum* and *I. squamulosohinnuleum* are members of this clade. The Maculatum clade is supported as monophyletic, and *I. sphaerobulbosum* is nested in this clade. *Inosperma nivalellum* is formed separate lineages in the Maculatum clade base (MLB = 94%, BPP < 0.90).

### Taxonomy

#### *Inosperma nivalellum* S. N. Li, Y. G. Fan, and Z. H. Chen, sp. nov.

Figures 2, 3

**FIGURE 2:**
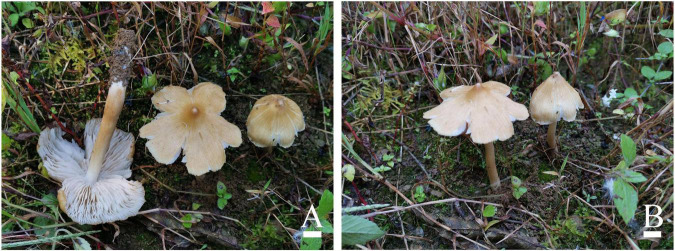
Basidiomata of *Inosperma nivalellum*. **(A,B)** MHHNU31689, holotype. Scale bars = 10 mm.

**FIGURE 3:**
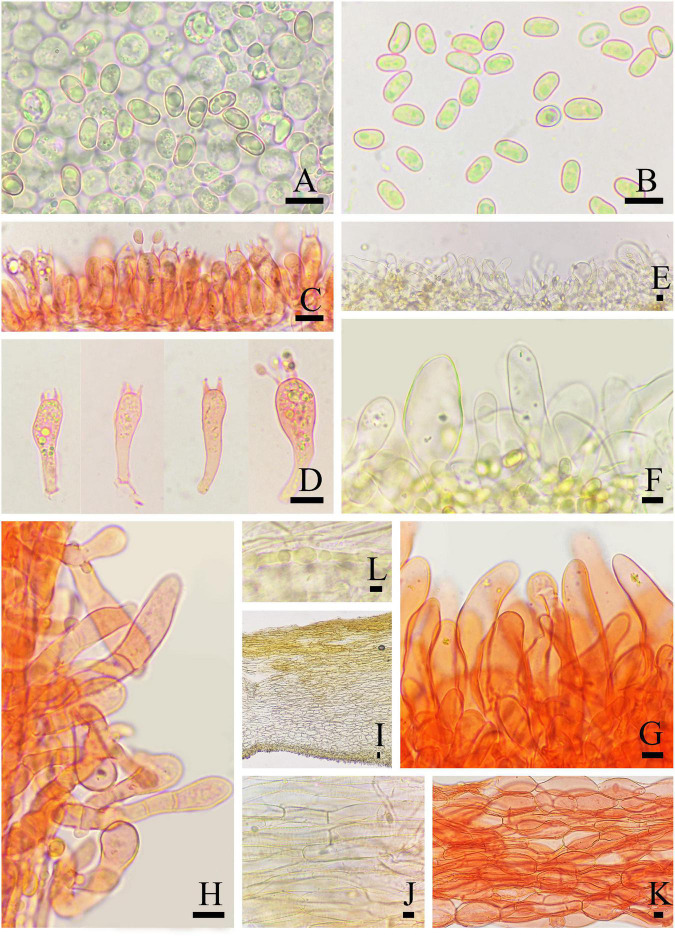
Microscopic features of *Inosperma nivalellum* (MHHNU31689, holotype). **(A,B)** Basidiospores; **(C,D)** basidia with probasidium; **(E)** gill edge; **(F,G)** cheilocystidia; **(H)** caulocystidia; **(I)** transverse section of pileus; **(J)** pileipellis; **(K)** hymenial hyphae; and **(L)** oleiferous hyphae. Scale bars = 10 μm.

Mycobank: 845644

Etymology: *Nivalis* (Latin), niveous; *lamella* (Latin), lamellae, referring to the snow-white hymenium.

Diagnostic features: Basidiomata medium to large. Pileus 40–91 mm in diameter, grayish yellow to light grayish yellow, smooth, radially fibrillose to rimulose, with a nipple-like umbo. Lamellae 2–5 mm wide, snow-white, adnexed. Stipe 68–100 × 5–11 mm, light grayish yellow in the middle and pure white at both ends. Basidiospores (6.0) 7.0–10.5 (12.0) μm × 4.0–5.5 μm, smooth, elliptic to oblong-elliptic or oblong-reniform. Cheilocystidia 22–75 × 5–29 μm, in clusters, various, mostly clavate, utriform, or narrowly cylindrical. Differs from *I. lanatodiscum* (Kauffman) Matheny and Esteve-Rav. by its larger basidiomata, nipple-like umbo, snow-white lamellae, and smaller basidiospores.

Holotype: CHINA. Hubei Province: Hefeng County, Xiaping Village, N: 110°13’93.8”, E: 30°05’98.1”, alt. 1680 m, on the ground in the subtropical montane forest dominated by *Castanea*, 22 September 2019, Z. H. Chen, P. Long, and Y. Q. Zeng, MHHNU31689 (GenBank accession no. ITS: OP135502; nrLSU: OP134006; *rpb2*: OP161556).

Basidiomata: Medium to large-sized. Pileus 40–91 mm wide, conical when young, then expanding to plane with a nipple-like umbo; margin decurved; surface dry, smooth, and unbroken in the center, radially fibrillose or rimulose elsewhere, rimose to deeply splitted when matured; veilpellis not observed, grayish yellow (4B5) around the center, paler outward, and light grayish yellow (4B4) toward the margin. Lamellae 2–5 mm wide, adnexed, often subsinuate, crowded (ca. 60–80); snow-white (1A1) even in mature basidiomata, yellowish upon drying, edge concolorous. Stipe 68–100 × 5–11 mm, cylindrical or attenuated toward apex; pure white (1A1) at the top and base, light grayish yellow (4B4) in the middle; nearly smooth, dry, and solid. Context snow-white (1A4) in stipe and pileus, not changing color upon exposure. Odor not recorded.

Basidiospores: (6.0) 7.0–10.5 (12.0) μm (av. 8.6 μm, SD 1.1 μm) × 4.0–5.5 μm (av. 4.8 μm, SD 0.5 μm), *Q* = (1.50) 1.56–2.25 (2.40), *Q*_*m*_ = 1.81 ± 0.22 (*n* = 80 of 4 coll.), smooth, oblong-elliptic or oblong-reniform, pale yellow with greenish tinge in 5% KOH. Basidia 28–35 × 8–10 μm, clavate to broadly clavate, 4-sterigmate, hyaline, or contain some oily inclusions. Pleurocystidia absent. Cheilocystidia 22–75 × 5–29 μm, in clusters, various, various, mostly clavate, utriform, or narrowly cylindrical with tapered apex, less often broadly fusiform, occasionally with a flexuous outline, apices often tapered, hyaline, thin-walled, walls yellowish-green in 5% KOH. Hymenophoral trama subregularly arranged, pale yellow to nearly colorless in 5%KOH, composed of smooth, thin-walled, and inflated hyphae 3–52 μm wide. Caulocystidia 22–60 × 7–24 μm, present at stipe apex, multiseptate, clavate to fusiform, sometimes with rounded to subcapitate apices, hyaline. Pileipellis is a cutis of coarsely encrusted and slightly expanded cylindrical hyphae mostly 6–29 μm wide regular, pale yellowish brown in 5% KOH; pileal trama composed of smooth, thin-walled, expanded cylindrical hyphae mostly 7–36 μm wide, regular to subregular, and nearly colorless in 5% KOH. Oleiferous hyphae present in pileal and stipe trama, 4–12 μm in diameter. Clamp connections are present and common in all tissues.

Known distribution: Known from the type locality in Hubei Province, China.

Habitat: Single to scattered on soil in subtropical montane forest dominated by *Castanea*.

Commentary: Morphologically, *I. lanatodiscum*, a widely distributed species in north temperate regions, is similar to *I. nivalellum* in its grayish yellow, distinctly umbonate, radially appressed fibrillose and rimose pileus. However, it differs from *I. nivalellum* in its smaller basidiomata (Pileus 3.5–4.5 cm wide), umbo not nipple-like, grayish white to yellowish brown lamellae, and larger basidiospores (9–11 × 5.5–6.5 μm) (Grund and Stuntz, 1968; Kropp et al., 2013; Fan and Bau, 2018). Phylogenetically, the two species are distant.

#### *Inosperma sphaerobulbosum* S. N. Li, Y. G. Fan, and Z. H. Chen, sp. nov.

Figures 4, 5

**FIGURE 4:**
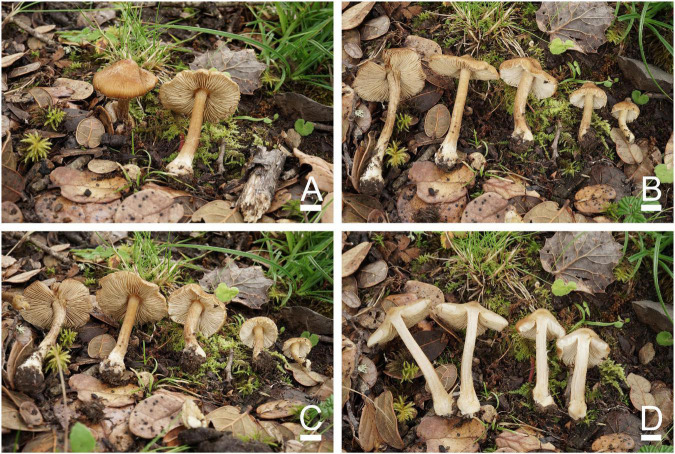
Basidiomata of *Inosperma sphaerobulbosum*. **(A–D)** MHHNU32266, holotype. Scale bars = 10 mm.

**FIGURE 5:**
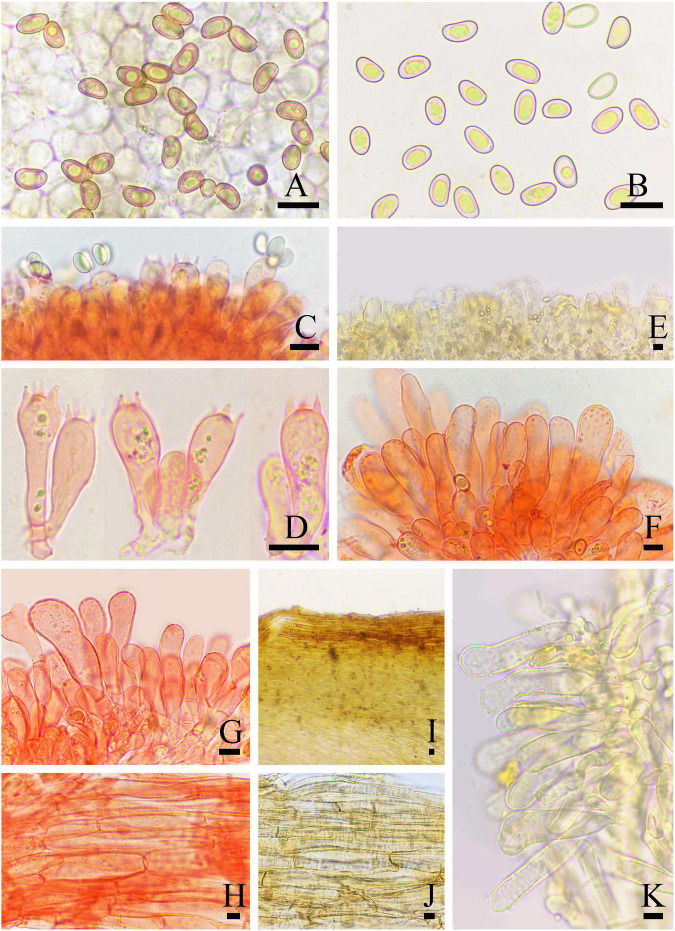
Microscopic features of *Inosperma sphaerobulbosum* (MHHNU32266, holotype). **(A,B)** Basidiospores; **(C,D)** basidia with probasidium; **(E)** gill edge; **(F,G)** cheilocystidia; **(H)** hymenial hyphae; **(I,J)** pileipellis; and **(K)** caulocystidia. Scale bars = 10 μm.

Mycobank: 845643

Etymology: *Sphaera* (Greek), spherical; *bulbous* (Latin), bulbus, referring to the bulbous base of the stipe.

Diagnostic features: Fruit bodies small to medium. Pileus 18–37 mm in diameter, brown orange to gray orange, fibrillose and rimose, with an obtuse umbo and obvious white veilpellis, especially in young pileus. Lamellae 3–4 mm wide, adnexed, milk white to grayish yellow or grayish orange. Stipe 18–55 × 3–6 mm, covered with white, woolly vesture when young; surface striped, concolorous with pileus, base enlarging into distinctive bulbous up to 13 mm wide. Odor spermatic. Basidiospores (6.5) 7.0–10.5 (11.0) μm × (4.5) 5.0–5.5 (6.0) μm, smooth, oblong-elliptic to oblong-phaseoliform. Cheilocystidia 30–65 × 7–25 μm, in clusters, cylindrical, broadly fusiform, or broadly clavate. Differs from *I. cookei* (Bres.) Matheny and Esteve-Rav. by its brown orange to gray orange pileus with obvious white veilpellis, stipe concolorous with pileus (except for the stipe base), larger and rounder bulbous base and spermatic odor.

Holotype: CHINA. Yunnan Province: Diqing Tibetan Autonomous Prefecture: Shangri-La, Pudacuo National Park, N: 99°96’29.6”, E: 27°90’53.6”, alt. 3800 m, on ground in cold temperate montane forests dominated by *Abies georgei* Orr var. *smithii* (Viguie et Gaussen) Cheng et L. and *Quercus semecarpifolia* Smith, 24 August 2020, Z. H. Chen, S. N. Li, and P. Long, MHHNU32266 (GenBank accession no. ITS: OP135501; nrLSU: OP134001; *rpb2*: OP161559).

Basidiomata: Small to medium-sized. Pileus 18–37 mm wide, conical, expanding with age, eventually plane and with a slightly decurved margin and an obtuse umbo; surface dry, smooth; white veilpellis covering entire pileus, and appendiculate veil remnants at the pileus margin when young, veilpellis gradually disappeared, fibrillose and rimose toward the margin with age; center or umbo brown orange (5C6), grayish orange (5B6) toward the margin. Lamellae 3–4 mm wide, adnexed, crowded (ca. 70–90); milk white (1A2), becoming grayish yellow (4B4) to grayish orange (5B4) with age. Stipe 18–55 × 3–6 mm, cylindrical or attenuated toward apex, base abruptly enlarges to bulbose up to 13 mm wide; with white, woolly vesture when young; after maturing, surface striped, apex with few scattered pallid furfuraceous fibrils, elsewhere finely fibrillose, concolorous with pileus, base milk white (1A2). Context milk white (1A2) to yellowish white (2A2) in stipe and grayish yellow (4B3) in pileus, not changing color upon exposure. Odor spermatic.

Basidiospores: (6.5) 7.0–10.5 (11.0) μm (av. 8.5 μm, SD 1.8 μm) × (4.5) 5.0–5.5 (6.0) (av. 5.1 μm, SD 0.4 μm) μm, *Q* = (1.30) 1.50–2.00 (2.10), *Q*_*m*_ = 1.64 ± 0.28 (*n* = 80 of 4 coll.), smooth, oblong-elliptic to oblong-phaseoliform, pale yellowish brown in 5% KOH, containing a bright yellow oily droplet inside. Basidia 29–35 × 8–11 μm, regular broadly clavate, 4-sterigmate, hyaline or contain some oily inclusions. Pleurocystidia absent. Cheilocystidia 30–65 × 7–25 μm, in clusters, cylindrical, or nearly, broadly fusiform or broadly clavate, sometimes with rounded to subcapitate apices and somewhat flexuose, thin-walled (occasionally coarse), occasionally septate, hyaline. Hymenophoral trama regularly to subregularly arranged, composed of thin-walled, cylindrical to inflated hyphae 3–27 μm wide. Caulocystidia 18–47 × 8–12 μm, present at the stipe apex, similar to cheilocystidia. Pileipellis is a cutis of coarsely encrusted (occasionally smooth), slightly expanded to inflated cylindrical hyphae mostly 6–29 μm wide, regular to subregular, yellowish brown in 5% KOH. Clamp connections are present and common in all tissues.

Known distribution: Known from the type locality in Yunnan Province, China.

Habitat: Scattered on the ground in cold temperate montane forests dominated by *Abies georgei* and *Quercus semecarpifolia.*

Commentary: In the phylogenetic tree (Figure 1), *Inosperma sphaerobulbosum* is nested in Maculatum Clade, and together with *I. quietiodor* Matheny and Esteve-Rav., *I. rimosoides* (Peck) Matheny and Esteve-Rav., *I.* cf. *reisneri* (Velen.) (Matheny and Esteve-Rav. 2019) and *I. shawarense* (A. Naseer and A. N. Khalid) Aïgnon and Naseer cluster into a branch with good support (MLB = 100%, BPP = 1.0). The common features of this branch are small to medium-sized basidiomata, fibrillose, and rimose pileus with umbonate, abruptly, or slightly swollen to bulbous stipe base, smooth, ellipsoid to phaseoliform spores (Kuyper, 1986; Naseer et al., 2017). *Inosperma cookei* shares a similar habitat (mostly under frondose trees, associated with *Fagus*, *Quercus*, *Castanea*, and *Corylus*.) and shape with *I. sphaerobulbosum*, but differs in its whitish to pale yellowish stipe not concolorous with pileus, honey-like smell and narrower, and shorter cheilocystidia (22–)28–42 × 11–18(–22) μm (Kuyper, 1986; Naseer et al., 2017).

#### *Inosperma longisporum* S. N. Li, Y. G. Fan and Z. H. Chen, sp. nov.

Figures 6, 7

**FIGURE 6:**
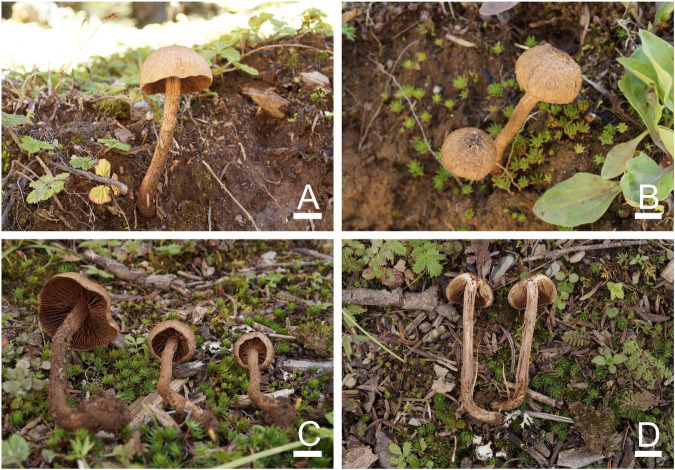
Basidiomata of *Inosperma longisporum*. **(A–D)** MHHNU32337, holotype. Scale bars = 10 mm.

**FIGURE 7:**
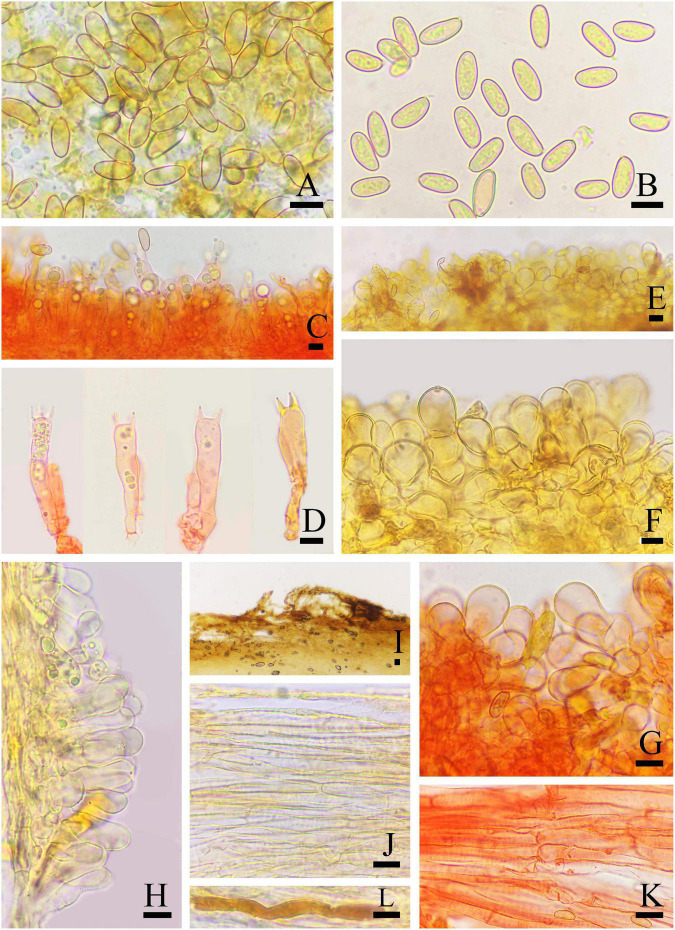
Microscopic features of *Inosperma longisporum* (MHHNU32337, holotype). **(A,B)** Basidiospores; **(C,D)** basidia with probasidium; **(E)** gill edge; **(F,G)** cheilocystidia and paracystidia; **(H)** caulocystidia and cauloparacystidia; **(I,J)** pileipellis; **(K)** hymenial hyphae; and **(L)** oleiferous hyphae. Scale bars = 10 μm.

Mycobank: 845645

Etymology: *Longior* (Latin), longer; *spora* (Latin), spore, referring to the longer spores.

Diagnostic features: Fruit bodies small to medium. Pileus 17–36 mm in diameter, subsphaeroidal to hemispherical, oak brown to coffee brown, with clumpy scales in the center, wooly fibrillose to coarsely fibrillose toward the margin. Lamellae oak brown to mustard brown, adnexed. Stipe 50–85 × 4–6 mm, cylindrical, equal or slightly enlarged at the base. Basidiospores (9.0) 12.0–17.0 μm × (5.0) 5.5–7.0 (8.0) μm, smooth, rice shape to cylindrical. Cheilocystidia 19–36 × 12–18 μm, squat cylindrical, broadly clavate to bulbous, at times septate, thin-walled to slightly thick-walled. Fresh odor like green corn. Differs from *I. mucidiolens* (Grund and D. E. Stuntz) Matheny and Esteve-Rav. by its oak brown to coffee brown pileus with clumpy scales in the center, oak brown to mustard brown lamellae that do not have a green color and squatter cheilocystidia.

Holotype: CHINA. Yunnan Province: Lijiang City, Yulong Naxi Autonomous County, Taian Township, N: 100°09’04.6”, E: 26°78’51.7”, alt. 2991 m, on the ground in subtropical montane forest dominated by *Abies georgei*, 23 August 2020, Z. H. Chen, S. N. Li, and P. Long, MHHNU32237 (GenBank accession no. ITS: OP135509; nrLSU: OP135495; *rpb2*: OP161560).

Basidiomata: Small to medium-sized. Pileus 1.7–3.6 mm wide, initially subsphaeroidal, becoming hemispherical to convex, at times obtusely conical, becoming plano-convex with age, not umbonate; margin initially inrolled, later decurved; the center covered with clumpy scales, wooly fibrillose to coarsely fibrillose toward the margin; veilpellis absent; oak brown (5D6) to coffee brown (5F8). Lamellae adnexed, crowded (ca. 40–70), up to 3 mm wide; oak brown (5D6), becoming mustard brown (5E6) with age, edge concolorous. Stipe 50–85 × 4–6 mm, cylindrical, equal or slightly enlarged at the base; solid; few furfuraceous scales at apex, with some loose fibrils toward the base; concolorous with pileus, sometimes base slightly lighter in color; surface dry. Context: milk white (1A2) pileus and stipe, stipe brunnescent upon exposure. Odor fresh like green corn in young specimens but spermatic in older material.

Basidiospores: (9.0) 12.0–17.0 μm (av. 13.3 μm, SD 2.0 μm) × (5.0) 5.5–7.0 (8.0) μm (av. 6.0 μm, SD 0.7 μm), *Q* = 1.80–2.58 (2.62), *Q*_*m*_ = 2.25 ± 0.23 (*n* = 80 of 4 coll.), smooth, rice shape to cylindrical (less often oblong-amygdaliform), yellowish green in 5% KOH. Basidia 35–50 × 9–10 μm, clavate to broadly clavate, 4-sterigmate, hyaline or contain some oily inclusions but necropigmented with age. Pleurocystidia absent. Cheilocystidia 19–36 × 12–18 μm, abundant and crowded, squat cylindrical, broadly clavate to bulbous, at times septate, thin-walled to slightly thick-walled, hyaline, sometimes the space is filled with brown mucilaginous material. Hymenophoral trama regularly to subregularly arranged, pale yellowish brown in 5% KOH, composed of thin walls with lightly rough, cylindrical to inflated hyphae 4–23 μm wide. Caulocystidia 15–35 × 5–12 μm, present at stipe apex, in clusters, broadly clavate to broadly cylindrical, with rounded to subcapitate apices, occasionally septate, hyaline. Pileipellis a cutis of coarsely encrusted cylindrical hyphae mostly 4–12 μm wide, regular, and pale yellowish brown in 5% KOH; pileal trama composed of smooth or weakly encrusted, expanded cylindrical hyphae mostly 4–20 μm wide, regular to subregular, pale yellow in 5% KOH. Oleiferous hyphae present in pileus and stipe trama, 3–7 μm in diameter. Clamp connections present and common in all tissues.

Known distribution: China (Yunnan and Sichuan Provinces).

Habitat: Scattered on the ground in subtropical montane forest dominated by *Abies georgei.*

Other examined specimens: CHINA. Sichuan: Province: Ganzi Tibetan Autonomous Prefecture, Kangding County, Gongga Township, Zimeiyakou, 8 August 2021, Z. H. Chen and P. Long, MHHNU33070.

Commentary: *Inosperma longisporum* formed a well-supported distinct lineage (MLB = 100%, BPP = 1.0) in the Cervicolores clade (Figure 1). In gross morphology, *I. longisporum* is similar to *I. mucidiolens* in shape and size of the basidiomata (pileus 10–40 mm and stipe 30–90 × 2–8 mm in *I. mucidiolens*), green corn odor and habitat under conifers. However, the lamellae and the stipe base of *I. mucidiolens* have a green color, the basidiospores (10–12 × 5–6 μm) are smaller and the cheilocystidia are longer [33–50(–55) × 10–20 μm] (Grund and Stuntz, 1970; Pradeep et al., 2016).

#### *Inosperma squamulosobrunneum* S. N. Li, Y. G. Fan, and Z. H. Chen, sp. nov.

Figures 8, 9

**FIGURE 8:**
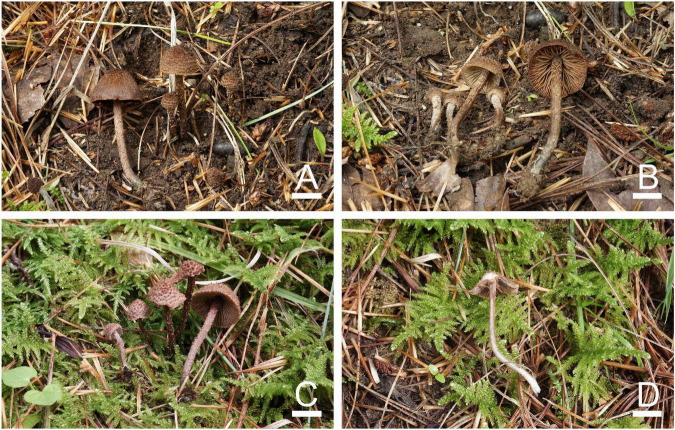
Basidiomata of *Inosperma squamulosobrunneum*. **(A,B)** MHHNU32359, holotype and **(C,D)** MHHNU32351. Scale bars = 10 mm.

**FIGURE 9:**
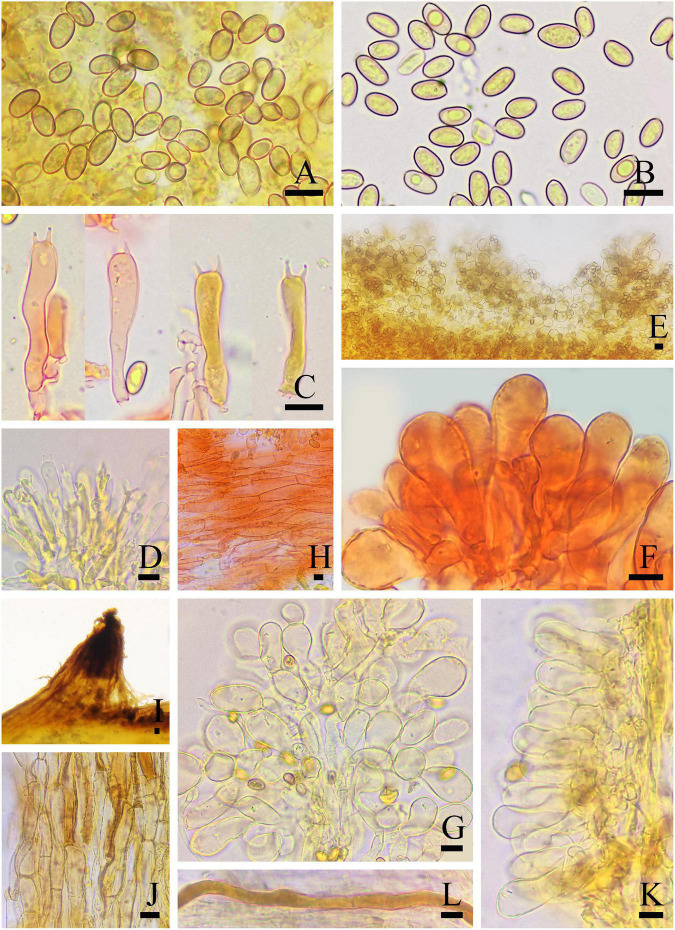
Microscopic features of *Inosperma squamulosobrunneum* (MHHNU32359, holotype). **(A,B)** Basidiospores; **(C,D)** basidia with probasidium; **(E)** gill edge; **(F,G)** cheilocystidia; **(H)** hymenial hyphae; **(I,J)** pileipellis; **(K)** caulocystidia; and **(L)** oleiferous hyphae. Scale bars = 10 μm.

Mycobank: 845646

Etymology: *Squamulosus* (*Latin*), squamulos*e*; *brunnes* (*Latin*), brown, referring to its pileus.

Diagnostic features: Fruit bodies small. Pileus 5–21 mm in diameter, oak brown to brown, with erect conical fibrillose squarrose around the center, upswept subsquarrose to squamulose toward the margin. Lamellae adnexed to ascending-adnate, yellowish white to clay brown. Stipe 24–50 × 1.5–3 mm, equal to a slightly swollen base. Basidiospores (7.5) 8.0–11 μm × (4.5) 5–7.0 μm, smooth, oblong-obovoid to oblong-ellipsoid. Cheilocystidia 22–48 × 11–19 μm, densely arranged like clusters of grapes. Odor fresh like corn leaf or mowed grass. Differs from *I. calamistratum* by its oak brown to brown pileus with erect conical fibrillose squarrose, smaller basidiomata, shorter spores, and fresh odor of corn leaf or mowed grass.

Holotype: CHINA. Yunnan Province: Dali Bai Autonomous Prefecture, Cangshan Mountain, *N*: 100°14’65.5”, E: 25°65’82.0”, alt. 2235 m, on the ground in subtropical montane forest dominated by *Pinus*, 23 August 2020, Z. H. Chen, S. N. Li and P. Long, MHHNU32359 (GenBank accession no. ITS: OP135499; nrLSU: OP134000; *rpb2*: OP161562).

Basidiomata: Small-sized. Pileus 5–21 mm, globular or conical when young, then hemispheric to conico-campanulate; with erect conical squamules around the center, outward more upswept fibrils subsquarrose to squamulose; appendiculate veil remnants at the pilells margin when young; oak brown (5D6) to brown (6D6), somewhat darker around the center. Lamellae adnexed to ascending-adnate, subcrowded (ca. 30–50), up to 5 mm broad, yellowish white (5A2) to clay brown (5D5), with distinct pallid-fimbriate edges. Stipe 24–50 × 1.5–3 mm, equal with a slightly swollen base, clay brown (5D5) to brown (6D6), discolor to grayish green (25C6) or dark grayish green (26D3) at the base, at apex minutely pruinose subflocculose, coarsely squamulose to squarrose in lower half, solid. Context milk white (1A2) in pileus and stipe, stipe context brunnescent after being cut. Smell strong, pleasant, like corn leaf or freshly mowed grass.

Basidiospores: (7.5) 8.0–11 μm (av. 9.2 μm, SD 1.0 μm) × (4.5) 5–7.0 μm (av. 5.4 μm, SD 0.7 μm), *Q* = (1.35) 1.45–2.00 (2.22), *Q*_*m*_ = 1.71 ± 0.15 (*n* = 80 of 4 coll.), smooth, oblong-obovoid to oblong-ellipsoid, yellowish brown in 5% KOH. Basidia 31–41 × 7–13 μm, narrowly clavate, slender, hyaline or contain some oily inclusions, becoming necropigmented, 4-sterigmate. Pleurocystidia absent. Cheilocystidia 22–48 × 11–19 μm, abundant and densely arranged like clusters of grapes, broadly clavate, pyriform or sphaeropedunculate, thin-walled, hyaline, most septate. Hymenophoral trama subregularly arranged, composed of thin-walled, cylindrical to inflated hyphae 2–24 μm wide. Caulocystidia present at the apex of stipe, 20–55 × 8–13 μm, in clusters, broadly clavate or pyriform. Pileipellis is a trichoderm of coarsely encrusted, sometimes smooth, slightly expanded to inflated cylindrical hyphae mostly 6–29 μm wide, regular to subregular, many cells filled with dark tawny resinoid content and collapsed, yellowish brown in 5% KOH. Oleiferous hyphae present in pileal and stipe trama, 4–10 μm in diameter. Clamp connections are seen on all hyphae.

Known distribution: China, Yunnan Province (Dali Bai Autonomous Prefecture and Chuxiong Yi Autonomous Prefecture).

Habitat: Gregarious or scattered in small clusters on the ground in subtropical montane forest dominated by *Pinus*.

Other examined specimens: CHINA. Yunnan Province: Dali Bai Autonomous Prefecture, Cangshan Mountain, 23 August 2020, Z. H. Chen, S. N. Li, and P. Long, MHHNU32351; Chuxiong Yi Autonomous Prefecture, Zixi Mountain, 21 August 2020, Z. H. Chen, S. N. Li, and P. Long, MHHNU32162; Baoshan City, Longling Country, Zhen’an Town, 11 July 2018, Y. G. Fan and W. J. Yu, FYG2869 (3608).

Commentary: Two other species, *I. calamistratum* and *I. cervicolor* (Pers.) Matheny and Esteve-Rav. also have conico-campanulate pileus with squamules around center, subsquarrose to squamulose toward the margin and coarsely squamulose to squarrose stipe equal or slightly swollen to the base, and symbiosis with *Pinus*. However, *I. calamistratum* differs in its pileus, which has dark brown squamules or recurvate scales at the center, contrasting with grayish brown background, strong disagreeable smell, larger basidiomata (pileus 10–38 mm, stipe 25–92 × 2–6 mm), and longer spores (on average 10.5–12.3 × 5.3–5.9 μm) (Kuyper, 1986). *Inosperma cervicolor* differs in its larger fruit bodies (pileus 6–40 mm, stipe 23–106 × 2–6 mm) and spores (on average 10.9–13.8 × 6.7–7.8 μm) and its stipe base does not discolor to grayish green or smell strongly, disagreeable, like old wine casks (Kuyper, 1986).

#### *Inosperma squamulosohinnuleum* S. N. Li, Y. G. Fan, and Z. H. Chen, sp. nov.

Figures 10, 11

**FIGURE 10:**
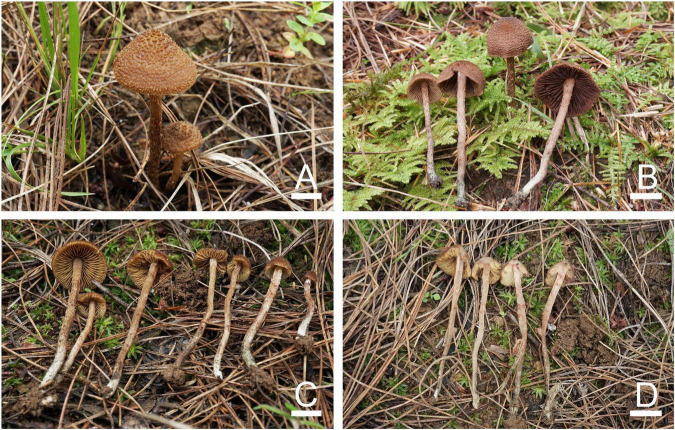
Basidiomata of *Inosperma squamulosohinnuleum*. **(A,C,D**) MHHNU32195, holotype and **(B)** MHHNU32362. Scale bars = 10 mm.

**FIGURE 11:**
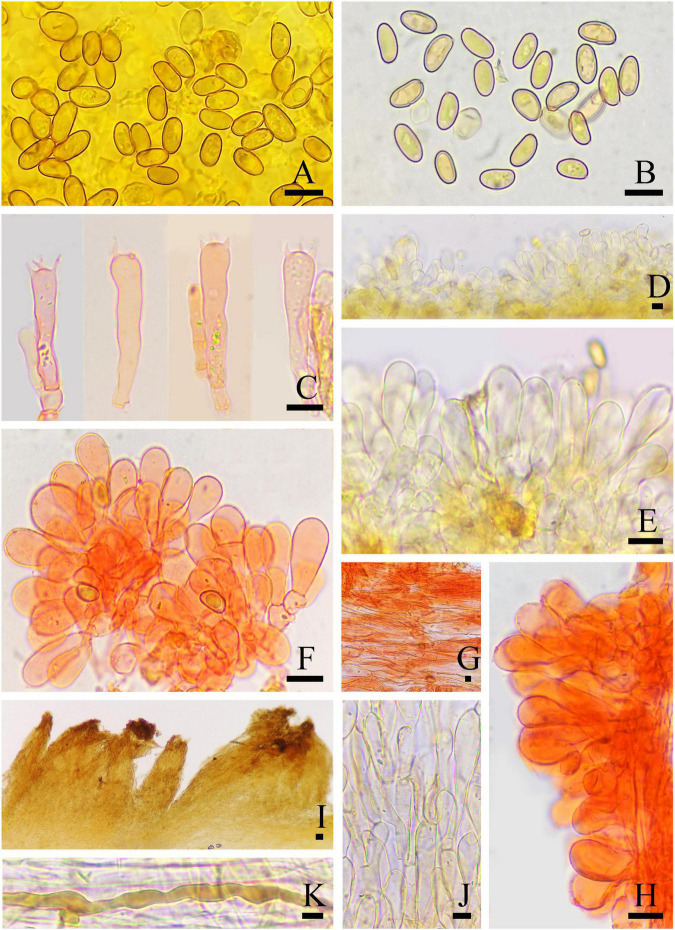
Microscopic features of *Inosperma squamulosohinnuleum* (MHHNU32195, holotype). **(A,B)** Basidiospores; **(C)** basidia with probasidium; **(D)** gill edge; **(E,F)** cheilocystidia; **(G)** hymenial hyphae; **(H)** caulocystidia; **(I,J)** pileipellis; and **(K)** oleiferous hyphae. Scale bars = 10 μm.

Mycobank: 845647

Etymology: *Squamulosus* (Latin), squamulose; *hinnuleus* (Latin), fawn, referring to its pileus.

Diagnostic features: Fruit bodies small to medium. Pileus 10–30 mm in diameter, reddish brown to caramel brown, with small, granular, erect squamulose at the disk, with numerous smaller furfuraceous scales toward the margin. Lamellae adnexed, pale yellowish white to brown. Stipe 35–70 × 2.5–4 mm, cylindrical, equal. Basidiospores (7.5) 8.0–11.5 μm × (4.0) 5.0–7.0 (7.5) μm, smooth, oblong-phaseoliform to oblong-ellipsoid, sometimes amygdaliform. Cheilocystidia 21–31 × 8–13 μm, in clusters, clavate to broadly clavate. Odor strong, pleasant, reminiscent of freshly mowed grass. Different from *I. squamulosobrunneum* in its denser and finer squamulose in the pileus, smaller, more uniform cheilocystidia, and smooth pileipellis.

Holotype: CHINA. Yunnan Province: Chuxiong Yi Autonomous Prefecture, Zixi Mountain, N: 100°16’26.7”, E: 25°64’24.0”, alt. 2354 m, on the ground in subtropical montane forest dominated by *Pinus*, 21 August 2020, Z. H. Chen, S. N. Li, and P. Long, MHHNU32195 (GenBank accession no. ITS: OP135500; nrLSU: OP134002; *rpb2*: OP161558).

Basidiomata: Small to medium sized. Pileus 10–30 mm in diameter, at first broadly and obtusely conical with decurved margin, becoming distinctly campanulate with age; with small, granular, erect squamulose at the disk, with numerous smaller furfuraceous scales toward the margin, veil remnants absent; reddish brown (5C5) to caramel brown (6C5), somewhat darker around the center. Lamellae adnexed, subcrowded (ca. 30–50), up to 3 mm wide, at first pale yellowish white (3A2), becoming brown (6E4), entire edges concolorous. Stipe 35–70 × 2.5–4 mm, cylindrical, equal, covered with concolorous, loose fibrils and scattered small scales, apex not pruinose, grayish orange (5B3) above, olive gray (16B3) at base; cortina present in young specimens, not persisting. Context pale yellowish white (2A2) to putty white in pileus, stipe context milk white (1A2) with pale olive gray (28B3) base and soon rufescent after exposure to air. Odor strong, pleasant, like freshly mowed grass.

Basidiospores: (7.5) 8.0–11.5 μm (av. 9.9 μm, SD 1.3 μm) × (4.0) 5.0–7.0 (7.5) μm (av. 5.6 μm, SD 0.8 μm), *Q* = (1.33) 1.53–2.18 (2.40), *Q*_*m*_ = 1.76 ± 0.22 (*n* = 80 of 4 coll.), smooth, oblong-phaseoliform to oblong-ellipsoid, sometimes amygdaliform, pale yellowish brown in 5% KOH. Basidia 32–46 × 8–10 μm, narrowly clavate, slender, hyaline or contain some oily inclusions, becoming necropigmented, 4-sterigmate. Pleurocystidia absent. Cheilocystidia 21–31 × 8–13 μm, abundant, in clusters, clavate to broadly clavate, thin-walled, hyaline. Hymenophoral trama: regularly to subregularly arranged, composed of thin-walled, cylindrical to inflated hyphae 3–32 μm wide. Caulocystidia present at the very apex of stipe, 22–60 × 7–24 μm, in clusters, similar to cheilocystidia. Pileipellis is a trichoderm of smooth, occasionally coarse, inflated cylindrical hyphae, 3–15 μm wide, regular to subregular, pale yellowish brown in 5% KOH. Oleiferous hyphae present in pileus and stipe trama, 3–10 μm in diameter. Clamp connections are seen on all hyphae.

Known distribution: China, Yunnan Province (Dali Bai Autonomous Prefecture and Chuxiong Yi Autonomous Prefecture).

Habitat: Scattered or gregarious in small clusters on the ground in subtropical montane forest dominated by *Pinus*.

Other examined specimens: CHINA. Yunnan Province: Dali Bai Autonomous Prefecture, Cangshan Mountain, 23 August 2020, Z. H. Chen, S. N. Li, and P. long, MHHNU32362; Pu’er City, Zhenyuan Country, Mengda Town, 22 September 2015, Y. G. Fan and B. Wang, on rich soil under mixed forests with Pinus and Quercus, FYG2015388 (FCAS3606), FYG2015399 (FCAS3607).

Commentary: *Inosperma squamulosohinnuleum* is similar to *I. squamulosobrunneum* in concrete morphological characters, odor, and spore size, but they can be distinguished with careful observation. *Inosperma squamulosobrunneum* has coarser, longer, granular and sparsely arranged scales in the pileus, coarsely encrusted pileipellis and smaller, more morphologically diverse cheilocystidia that are densely arranged like clusters of grapes. They are also far apart and located on different branches in the phylogenetic tree (Figure 1). There are several other species in the *Cervicolores* clade with a grayish-green stipe base, but all of them can be distinguished morphologically from *I. squamulosohinnuleum. Inosperma calamistratum* differs in its distinctive squarrose to recurved-scaly pileus, larger basidiomata and longer spores (Kuyper, 1986). *Inosperma calamistratoides* (E. Horak) Matheny and Esteve-Rav. differs in its dark reddish brown pileus with recurved-squamulose in the disk, and odor of *Pelargonium* (Matheny and Bougher, 2017). *Inosperma cyanotrichia* (Matheny, Bougher and G. M. Gates) Matheny and Esteve-Rav. and *I. viridipes* (Matheny, Bougher and G. M. Gates) Matheny and Esteve-Rav. are reported under *Eucalyptus* in Australia, and both have a greenish base of the stipe, but the surface of their pileus and stipe is fibrillose (not distinct scales) (Matheny and Bougher, 2017).

### Taxonomic key of *Inosperma* species in China

Phylogenetically, the genus *Inosperma* has six major lineages, namely, the *Cervicolores* clade, Maculatum clade, *I. misakaense* lineage, *I. africanum* lineage, Old World tropical clade 1 and Old World tropical clade 2, of which the *Cervicolores* clade, Maculatum clade and Old World tropical clade 2 have distributions in China. Among the 17 *Inosperma* species reported in China, five are distributed in the *Cervicolores* clade, seven in the Maculatum clade, and four in the Old World tropical clade 2.

The *Cervicolores* clade includes species characterized by tomentose, coarsely fibrillose to squarrose or squamulose pileus, ellipsoid to phaseoliform spores, and absent metuloid pleurocystidia but with densely packed, simple, cylindrical, clavate to pyriform hymenial cheilocystidia that make the gill edge look distinctly white in mature basidiomata. Other characteristics that may occur are a distinctly bulbous stem base, reddening flesh, yellow to olivaceous tinges on lamellae and specific odors (Kuyper, 1986; Larsson et al., 2009; Kropp et al., 2013; Latha and Manimohan, 2017).

The Maculatum clade is characterized by radially fibrillose to rimulose or rimose pileus, a smooth stipe (some with a distinctly bulbous base), context that changes color upon exposure (in several species), phaseoliform spores, thin-walled, often clavate to pyriform cheilocystidia, and distinctive odors (mostly non-spermatic) (Larsson et al., 2009; Kropp et al., 2013; Pradeep et al., 2016).

The members of Old World tropical clade 2 usually have medium-sized basidiomata, a gregarious habit, appressed-scaly or fibrillose-rimose pileus, rather crowded lamellae, longitudinally striate stipe, non-changing context, subglobose to elliptic basidiospores, and a lack of distinctive odors (Pradeep et al., 2016; Latha and Manimohan, 2017; Deng et al., 2021a,b, 2022).

Based on the above, we created a taxonomic key of the 17 *Inosperma* species reported in China as follows:

(1) Pileus tomentose, coarsely fibrillose, squarrose, squamulose; basidia necropigmented and slender…………. ……………………………………………………….. 2 (*Cervicolores* clade)
(1’) Pileus rimulose or rimose; basidia hyaline and not slender…………………………………………………………………………… 6
(2) Pileus covered with clumpy scales around disk, coarsely fibrillose toward margin, oak brown to coffee brown; odor fresh like green corn………………………………….. *I. longisporum*
(2’) Pileus squarrose or squamulose all over…………………………. 3
(3) Stipe never with blue–green tinges ……………….. *I. cervicolor*
(3’) Stipe in lower half with blue–green tinges ……………………… 4
(4) Pileus with dark brown squamules or recurvate scales at center, contrasting with grayish brown background; strong, disagreeable smell………………………………………………………. *I. calamistratum*
(4’) Pileus with even color surface, somewhat slightly darker around center………………………………………………………………… 5
(5) Pileus with erect fibrils conical squarrose around center; cheilocystidia densely arranged like clusters of grapes… …………………………………………………… *I. squamulosobrunneum*
(5’) Pileus with small, granular, erect squamulose at disk; cheilocystidia in clusters…………………………………………….. ………………………………………………….. *I. squamulosohinnuleum*
(6) Gregarious habit; pileus plano-convex with an obviously darker umbo, margin usually strongly rimose with age……………………………………. 7 (Old World tropical clade 2)
(6’) Single to scattered habit; pileus with even color surface, somewhat slightly darker around center,…. ……………………………………………………… 10 (Maculatum clade)
(7) Basidiospores subglobose to globose (*Q* = 1.00–1.27)….. ………………………………………………………… *I. subsphaerosporum*
(7’) Basidiospores ellipsoid, oblong-ellipsoid or ovoid (*Q*_*m*_ > 1.43) …………………………………………………………………. 8
(8) Pileus with thick, persistent, and zonate velar veil remnants……………………………………………… *I. zonativeliferum*
(8’) Veil remnants indistinct…………………………………………………. 9
(9) Basidiospores ellipsoid to enlongate-ellipsoid (*Q* = 1.42–1.86); cheilocystidia clavate, broadly clavate to enlongate-clavate ………………………………………………………… *I. muscarium*
(9’) Basidiospores mostly ellipsoid to ovoid (*Q* = 1.28–1.64); Cheilocystidia obovoid to balloon-shaped……………………………………………. *I. hainanense*
(10) Pileus with a nipple-like umbo; lamellae snow-white even in mature basidiomata………………………………….. *I. nivalellum*
(10’) Pileus with a subacute or obtuse umbo; lamellae not snow-white when mature ………………………………………………………. 11
(11) Basidiocarps change color with age or from damage… …………………………………………………………………………………….. 12
(11’) Basidiocarps do not change color with age or from damage… …………………………………………………………………….. 13
(12) Basidiocarps white to pale ochraceous, becoming orange to brick red with age or damage………………………… *I. erubescens*
(12’) Basidiocarps vinaceous, pinkish red and change color to black with age or after touching…………………………………….. ………………………………………………………………….. *I. adaequatum* (Britzelm.) Matheny and Esteve-Rav.
(13) Stipe base distinctly bulbous ……………… *I. sphaerobulbosum*
(13’) Stipe even or with a swollen base, but not marginate….. …………………………………………………………………………………….. 14
(14) Pileus orange to brownish yellow……………… *I. lanatodiscum*
(14’) Pileus not orange to brownish yellow……………………………. 15
(15) Stipe smooth……………………………………………. *I. changbaiense* (T. Bau and Y. G. Fan) Matheny and Esteve-Rav.
(15’) Stipe not smooth, minutely subflocculose apically or longitudinally striated on surface…………………………………. 16
(16) Basidiospores phaseoliform, rarely ellipsoid (*Q* = 1.61–2.08)…………………………………………………….. *I. neobrunnescens* (Grund and D. E. Stuntz) Matheny and Esteve-Rav.
(16’) Basidiospores ellipsoid to oblong-ellipsoid, not (or hardly) phaseoliform (*Q* = 1.60–1.88)……………………… *I. quietiodor.*

### Toxin detection

**FIGURE 12:**
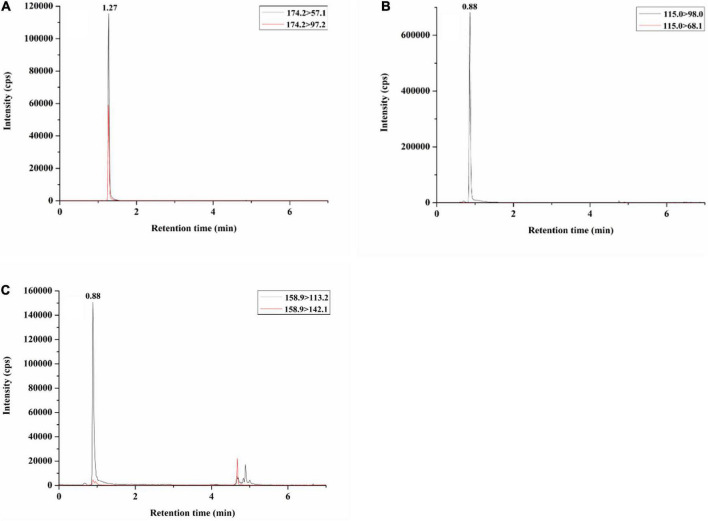
Total ion current (TIC) chromatogram of neurotoxins in positive mushroom samples: **(A)** muscarine (MHHNU 31689); **(B)** muscimol (MHHNU 32359); and **(C)** ibotenic acid (MHHNU 33070).

Through UPLC-MS/MS detection, we found that mushroom neurotoxins were absent in *Inosperma sphaerobulbosum*; *I. nivalellum* was muscarine positive; *I. longisporum* and *I. squamulosohinnuleum* contained both ibotenic acid and muscimol; *I. squamulosobrunneum* only contained muscimol; and psilocybin was not detected in these five new species (Table 2 and Supplementary Data Sheet 2, UPLC-MSMS Original Data). Representative chromatograms of muscarine, ibotenic acid, and muscimol are shown in Figure 12.

**TABLE 2:** Relative mushroom neurotoxins concentrations measured by UPLC-MS/MS.

Species	Muscarine	Psilocybin	Ibotenic acid	Muscimol
*Inosperma longisporum* MHHNU32337	–	–	–	–
*Inosperma longisporum* MHHNU33070	–	–	3.92 ± 0.73	43.97 ± 8.18
*Inosperma nivalellum* MHHNU31689	969.11 ± 180.25	–	–	–
*Inosperma sphaerobulbosum* MHHNU32266	–	–	–	–
*Inosperma squamulosobrunneum* MHHNU32162	–	–	–	95.3 0 ± 18.72
*Inosperma squamulosobrunneum* MHHNU32351	–	–	–	167.16 ± 31.09
*Inosperma squamulosobrunneum* MHHNU32359	–	–	–	341.22 ± 63.47
*Inosperma squamulosohinnuleum* MHHNU32195	–	–	–	–
*Inosperma squamulosohinnuleum* MHHNU32362	–	–	1.89 ± 0.35	18.23 ± 3.39

Unit: mg/kg.

## Discussion

### Toxicity in *Inosperma*

Muscarine is found in clinically significant amounts in the basidiomata of several distantly related groups of mushroom-forming fungi (*Clitocybe sensu lato*, *Mycena*, *Omphalotus*, Inocybaceae) and is particularly widespread in Inocybaceae (Benjamin, 1995; Chen et al., 2016). A variety of methods have been used to detect muscarine in recent years (Fahrig, 1920; Eugster, 1957; Brown et al., 1962; Robbers et al., 1964; Kosentka et al., 2013; Yoshioka, 2014; Xu et al., 2020), and its detected content in Inocybaceae ranges from ca. 0.06–16000 mg/kg (Kosentka et al., 2013; Deng et al., 2021a). Eight *Inosperma* species have been reported to contain muscarine, including *I. carnosibulbosum*, *I. erubescens*, *I. hainanense*, *I. maculatum*, *I. muscarium*, *I. vinaceobrunneum*, *I. virosum*, and *I. zonativeliferum* (Kosentka et al., 2013; Bijeesh et al., 2020; Latha et al., 2020; Deng et al., 2021a,^2022^). Among these muscarine-positive species, the muscarine content has been reported for the following species: *I. hainanense*, 11870 ± 3020 mg/kg, *I. muscarium*, 16030 ± 1230 mg/kg (Deng et al., 2021a), *I. virosum*, 270 or 300 mg/kg (Sailatha et al., 2014; Latha et al., 2020), and *I. zonativeliferum*, 2080 ± 50 mg/kg. In our study, the muscarine content in *I. nivalellum* was 969.11 ± 180.25 mg/kg, which is in the range of previous reports. For humans, the lethal dose of muscarine is not precisely known, with estimates ranging from 40 to 495 mg (Pauli and Foot, 2005), equivalent to 0.4–5 kg of fresh *I. nivalellum* fruit bodies. However, *I. nivalellum* has large fruiting bodies and is somewhat similar to the edible fungus *Termitomyces microcarpus* (Berk. and Broome) R. Heim, with a scattered habitat, and people may collect a large number of them at once, thus increasing the risk of ingesting them and causing poisoning or death.

Psilocybin, the main toxin of the *Psilocybe* genus, was first demonstrated in *Inocybe* in the 1980s (Guzmán et al., 1998). A literature review of muscarine and psilocybin reports showed that the following species lack muscarine but possess psilocybin: *Inocybe aeruginascens* Babos, *I. coelestium* Kuyper, *I. corydaline* Quél., *I. haemacta* (Berk. and Cooke) Sacc., and *I. tricolor* Kühner (Besl and Mack, 1985; Stijve et al., 1985; Kosentka et al., 2013). To date, except for the genus *Inocybe*, psilocybin has not been detected from any other genera in the family Inocybaceae. We determined the psilocybin content in the five new species of *Inosperma*, and the results show that all of the samples were negative, which is consistent with the results of previous studies.

In this study, the presence of ibotenic acid and muscimol in *Inosperma* species was identified for the first time, ranging from 1.89 ± 0.35 mg/kg to 3.92 ± 0.73 mg/kg and 18.23 ± 8.18 mg/kg to 341.22 ± 63.47 mg/kg, respectively (in the caps, dry weight). In earlier investigations, the contents of ibotenic acid and muscimol in the caps (wet weight) from *Amanita muscaria* were determined to be 519 mg/kg and 253 mg/kg (Tsunoda et al., 1993), 990 and 380 mg/kg (Gennaro et al., 1997), and 170 and 0 mg/kg, respectively (StØrmer et al., 2004). A recent study found that *Paraisaria heteropoda* also contains ibotenic acid, at 0.1 mg/kg, but muscimol was not found (Kobayasi, 1952). The concentration and distribution of toxins in mushrooms are variable and depend on several factors, mainly on their origin, growing environment, time of collection, and storage conditions (StØrmer et al., 2004; Ginterová et al., 2014); for example, the concentration of ibotenic acid in hallucinogenic mushrooms decreases over time due to its transformation into muscimol (a more pharmacologically active toxin) during mushroom drying (Ginterová et al., 2014), which may explain the discrepancy. The psychoactive dose of ibotenic acid is approximately 30–90 mg, and that of muscimol is approximately 6–10 mg (Eugster, 1967; Waser, 1967). Consequently, *I. squamulosobrunneum* is considered to be a highly dangerous *Inosperma* species when mistakenly ingested by humans. *Inosperma longisporum* and *I. squamulosohinnuleum* contain lower levels of ibotenic acid and muscimol, but eating large amounts of them may also cause poisoning.

### Relationship of phylogenetic and toxins in *Inosperma*

Few studies have examined the distribution of toxins within the systematic framework of Inocybaceae, let alone the genus *Inosperma*, to determine their evolutionary significance (Brown et al., 1962; Catalfomo and Eugster, 1970; Kosentka et al., 2013). Previously, it was hypothesized that the presence of muscarine had little taxonomic correlation (Brown et al., 1962). However, Stijve et al. (1985) suggested that the presence of muscarine may not be entirely random taxonomically. Unfortunately, these studies were hindered by a lack of robust phylogenetic hypotheses against which to map the distribution of muscarine-containing species. Kosentka et al. (2013) evaluated the evolution of muscarine and psilocybin in Inocybaceae. They detected that muscarine is not ancestral for the family as a whole, but it is a shared derived trait for an inclusive clade containing three of the seven major lineages of Inocybaceae (the *Inocybe*, *Nothocybe*, and *Pseudosperma* clades) and predicted that the species of the *Inosperma* clade lack muscarine. As an increasing number of muscarine-containing species of *Inosperma* have been reported in recent years (Bijeesh et al., 2020; Latha et al., 2020; Deng et al., 2021a,^2022^), we believe that it is an important muscarine-containing genus in the family Inocybaceae.

In the framework of *Inosperma*, we found that muscarine is mainly distributed in the Maculatum clade and Old World tropical clade 2. *Inosperma nivalellum* forms separate lineages in the Maculatum clade base (MLB = 94%, BPP < 0.90), but morphologically, *I. nivalellum* have radially fibrillose or rimulose pileus, a smooth stipe, and oblong-elliptic or oblong-reniform spores, which is consistent with the characteristics of the Maculatum clade. Regarding the toxicity, *I. nivalellum* contains muscarine, and the Maculatum clade is one of the main muscarine-containing clades of *Inosperma*. In conclusion, *I. nivalellum* should belong to the Maculatum clade. Ibotenic acid and muscimol were first discovered in *Inosperma* species and are mainly distributed in the *Cervicolores* clade. Psilocybin has not been detected in *Inosperma*.

## Data availability statement

The datasets presented in this study can be found in online repositories. The names of the repository/repositories and accession number(s) can be found in the article/Supplementary material.

## Author contributions

Z-HC: conceptualization. FX, Y-GF, and Z-HC: funding acquisition. S-NL and FX: investigation. FX and Y-GF: methodology. S-NL, PL, PZ, Y-GF, and Z-HC: resources. FL: visualization. S-NL: writing–original draft. Y-GF: writing–review and editing. All authors read and agreed to the published version of the manuscript.

## Footnotes

1
https://www.ncbi.nlm.nih.gov/genbank

2
http://iqtree.cibiv.univie.ac.at/

## Supplementary material

Supplementary Figure 1 Phylogenetic relationship and placement of *Inosperma* inferred from the combined dataset (ITS, nrLSU, and rpb2) using ML phylogenetic methods.

Supplementary Figure 2 Phylogenetic relationship and placement of *Inosperma* inferred from the combined dataset (ITS, nrLSU, and rpb2) using BI phylogenetic methods.

Supplementary Table 1 Taxon sampling information and DNA sequences used for phylogenetic analyses.

Supplementary Data Sheet 1 Combined sequences (ITS, nrLSU, and rpb2) alignment dataset.

Supplementary Data Sheet 2 UPLC-MSMS Original Data.
