# Supplementary figures and images for: Five new species of Inosperma from China: Morphological characteristics, phylogenetic analyses, and toxin detection
Source: Front Microbiol. 2022 Oct 31;13:1021583. doi: 10.3389/fmicb.2022.1021583 (PMC9659589; doi:10.3389/fmicb.2022.1021583)

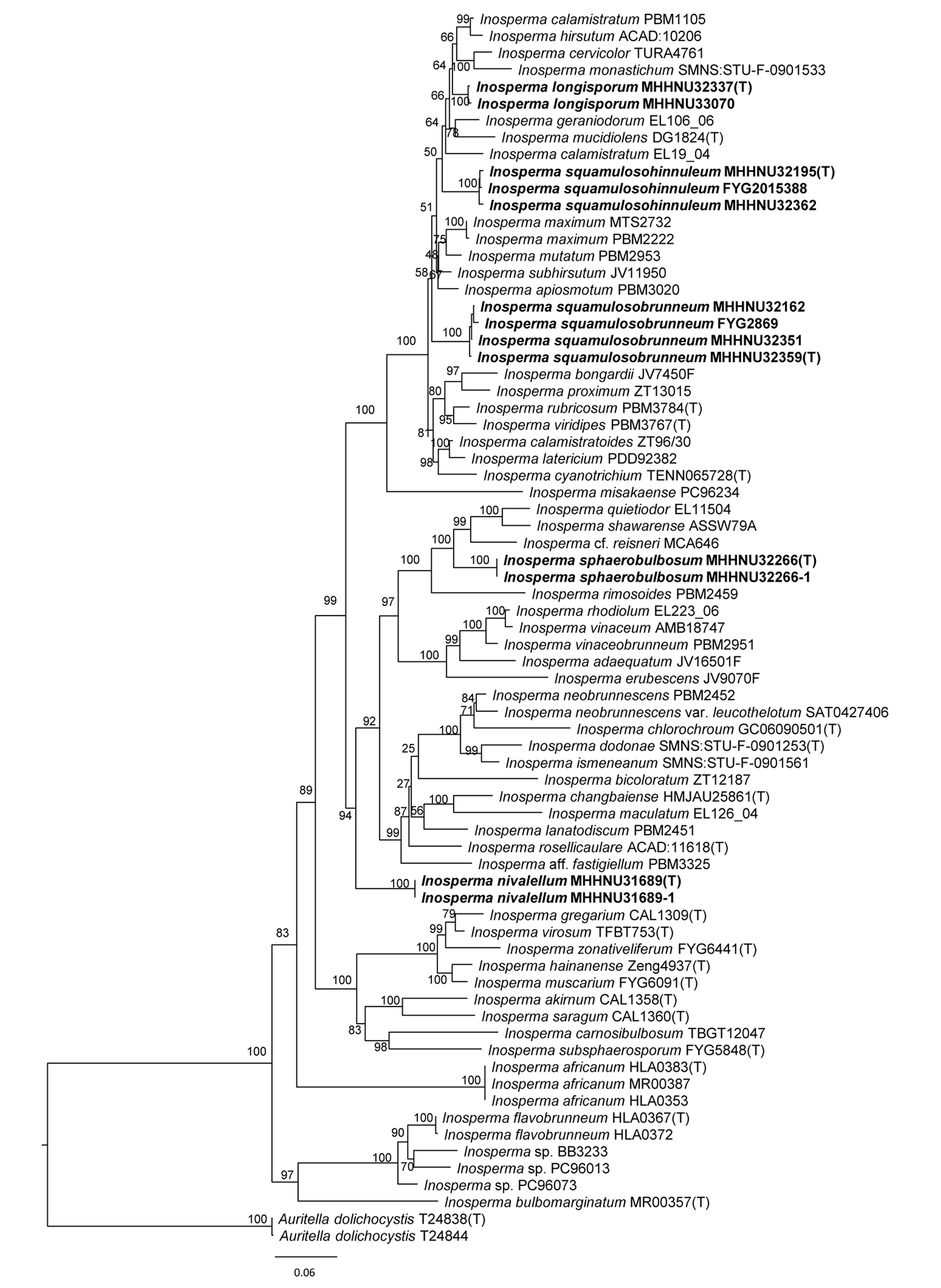

Supplement: Supplementary Figure 1 — Phylogenetic relationship and placement of Inosperma inferred from the combined dataset (ITS, nrLSU, and rpb2) using ML phylogenetic methods. [file Image_1.TIF]

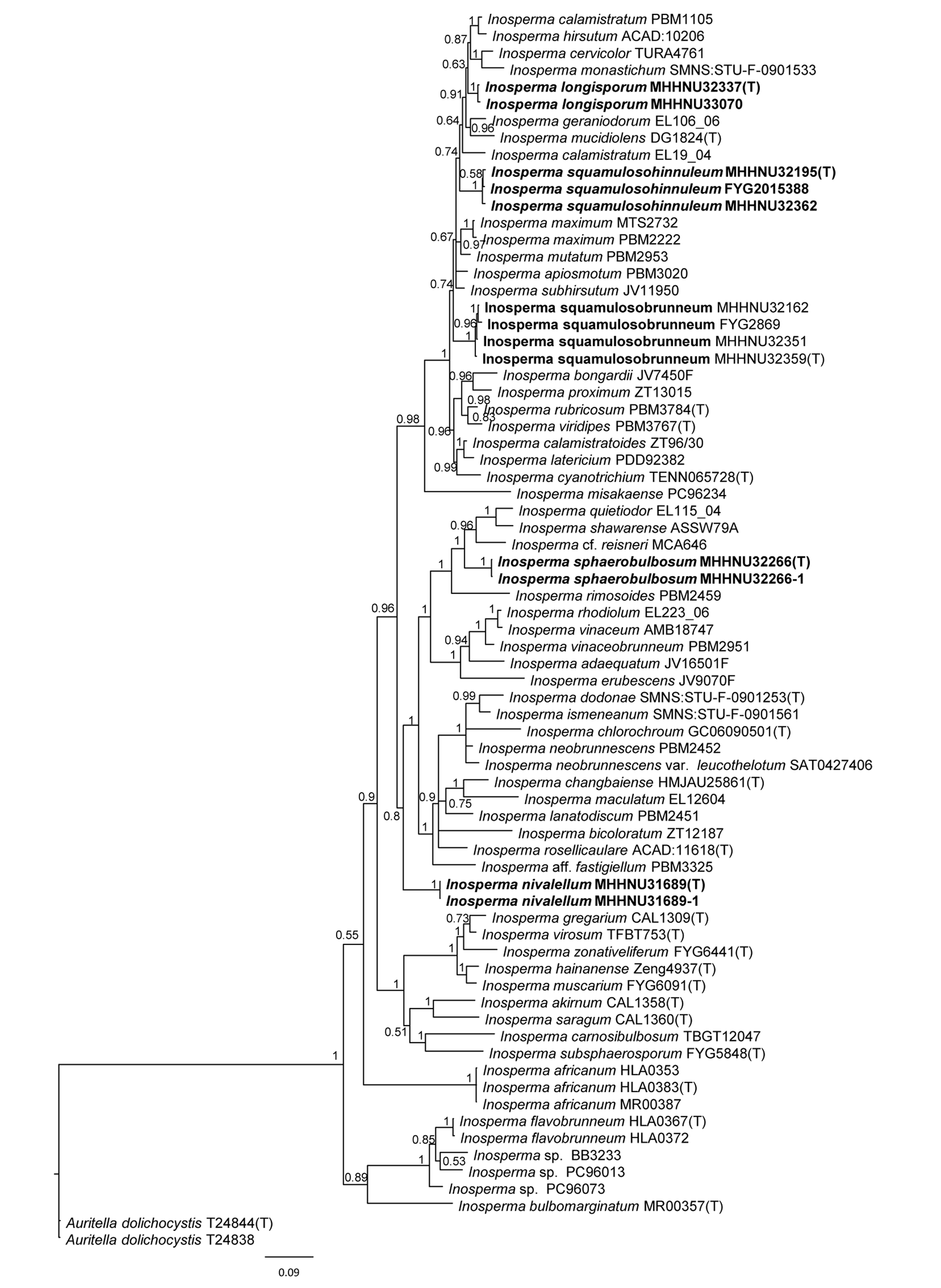

Supplement: Supplementary Figure 2 — Phylogenetic relationship and placement of Inosperma inferred from the combined dataset (ITS, nrLSU, and rpb2) using BI phylogenetic methods. [file Image_2.TIF]
