# Supplementary material for: Five new species of Inosperma from China: Morphological characteristics, phylogenetic analyses, and toxin detection
Source: Front Microbiol. 2022 Oct 31;13:1021583. doi: 10.3389/fmicb.2022.1021583 (PMC9659589; doi:10.3389/fmicb.2022.1021583)
Supplement: Supplementary Table 1 — Taxon sampling information and DNA sequences used for phylogenetic analyses. [file Table_1.DOCX]

Supplementary Material

**Table S1.** Taxon sampling information and DNA sequences used for phylogenetic analyses

| **Species** | **Voucher** | **Locality** | **ITS** | **nrLSU** | ***rpb2*** | **References** |
| --- | --- | --- | --- | --- | --- | --- |
| ***INOSPERMA*** |  |  |  |  |  |  |
| *Inosperma adaequatum* | JV16501F | Finland | N/A | AY380364 | AY333771 | Matheny et al. (2019) |
| *Inosperma africanum* | MR00387 | Togo | MN096189 | MN097881 | MT770739 | Aïgnon et al. (2021) |
| *Inosperma africanum* | HLA0383 (T) | Benin | MT534298 | MT560733 | N/A | Aïgnon et al. (2021) |
| *Inosperma africanum* | HLA0353 | Benin | MT534299 | N/A | N/A | Aïgnon et al. (2021) |
| *Inosperma akirnum* | CAL1358(T) | India | KY440085 | NG_057279 | KY553236 | Matheny et al. (2019) |
| *Inosperma apiosmotum* | PBM3020 | USA | JQ801385 | JN975021 | JQ846463 | Matheny et al. (2019) |
| *Inosperma bicoloratum* | ZT12187 | Malaysia | GQ892984 | GQ892938 | JQ846464 | Pradeep et al. (2016) |
| *Inosperma bongardii* | JV7450F | Finland | N/A | EU555448 | N/A | Pradeep et al. (2016) |
| *Inosperma bulbomarginatum* | MR00357 (T) | Benin | MN096190 | MN097882 | MN200775 | Aïgnon et al. (2021) |
| *Inosperma calamistratum* | EL19_04 | Sweden | AM882938 | AM882938 | N/A | Pradeep et al. (2016) |
| *Inosperma calamistratum* | PBM1105 | USA | JQ801386 | JQ815409 | JQ846466 | Matheny et al. (2019) |
| *Inosperma calamistratoides* | ZT96/30 | New Zealand | JQ801392 | JQ815413 | AY333765 | Matheny and Bougher (2017) |
| *Inosperma carnosibulbosum* | TBGT12047 | India | KT329448 | KT329454 | KT329443 | Pradeep et al. (2016) |
| *Inosperma cervicolor* | TURA4761 | Finland | JQ801395 | JQ815417 | JQ846474 | Pradeep et al. (2016) |
| *Inosperma changbaiense* | HMJAU25861(T) | China | NR_160610 | NG_066407 | MT086755 | Fan and Bau (2018) |
| *Inosperma chlorochroum* | GC06090501(T) | France | MK508903 | N/A | N/A | TYPE material |
| *Inosperma cyanotrichium* | TENN065728(T) | Australia | NR_153127 | JN975033 | JQ846476 | Pradeep et al. (2016) |
| *Inosperma dodonae* | STU:SMNS-STU-F-0901253(T) | Netherlands | NR_173967 | N/A | N/A | Bandini et al. (2021) |
| *Inosperma erubescens* | JV9070F | Finland | N/A | EU569846 | N/A | Pradeep et al. (2016) |
| *Inosperma aff. fastigiellum* | PBM3325 | USA | JQ801399 | JQ815419 | JQ846477 | Pradeep et al. (2016) |
| *Inosperma flavobrunneum* | HLA0372 | Benin | MT534290 | MT536756 | N/A | Aïgnon et al. (2021) |
| *Inosperma flavobrunneum* | HLA0367 (T) | Benin | MN096199 | N/A | N/A | Aïgnon et al. (2021) |
| *Inosperma geraniodorum* | EL106_06 | Sweden | FN550945 | FN550945 | N/A | Pradeep et al. (2016) |
| *Inosperma gregarium* | CAL1309(T) | India | NR_153174 | KX852306 | KX852307 | Latha and Manimohan (2017) |
| *Inosperma hainanense* | Zeng4937 (T) | China | MZ374070 | MZ374761 | MZ388104 | Deng et al. (2021a) |
| *Inosperma hirsutum* | ACAD:10206 | Canada | MH024863 | MH539758 | N/A | Unpublished |
| *Inosperma ismeneanum* | STU:SMNS-STU-F-0901561 | Germany | MW647625 | N/A | N/A | Bandini et al. (2021) |
| *Inosperma lanatodiscum* | PBM2451 | USA | JQ408759 | JQ319690 | JQ846483 | Pradeep et al. (2016) |
| *Inosperma latericium* | PDD92382 | New Zealand | GU233367 | GU233413 | N/A | Pradeep et al. (2016) |
| ***Inosperma longisporum*** | **MHNNU32337(T)** | **China** | **OP135509** | **OP135495** | **OP161560** | **This study** |
| ***Inosperma longisporum*** | **MHNNU33070** | **China** | **OP135504** | **OP133999** | **OP161564** | **This study** |
| *Inosperma maculatum* | EL126_04 | Sweden | AM882964 | AM882964 | N/A | Pradeep et al. (2016) |
| *Inosperma maximum* | PBM2222 | USA | N/A | EU569854 | N/A | Pradeep et al. (2016) |
| *Inosperma maximum* | MTS2732 | USA | JQ801400 | JQ815420 | N/A | Unpublished |
| *Inosperma misakaense* | PC96234 | Zambia | JQ801409 | EU569874 | EU569873 | Pradeep et al. (2016) |
| *Inosperma monastichum* | STU:SMNS-STU-F-0901533 | Germany | MW647631 | N/A | N/A | Bandini et al. (2021) |
| *Inosperma mucidiolens* | DG1824(T) | Canada | HQ201339 | HQ201340 | N/A | Pradeep et al. (2016) |
| *Inosperma muscarium* | FYG6091 (T) | China | MZ373982 | MZ373991 | MZ388093 | Deng et al. (2021a) |
| *Inosperma mutatum* | PBM2953 | USA | JQ801410 | JQ994476 | JQ846488 | Matheny et al. (2019) |
| *Inosperma neobrunnescens* | PBM2452 | USA | N/A | EU569868 | N/A | Pradeep et al. (2016) |
| *Inosperma neobrunnescens var. leucothelotum* | SAT0427406 | USA | JQ801411 | JN975025 | N/A | Pradeep et al. (2016) |
| ***Inosperma nivalellum*** | **MHNNU31689(T)** | **China** | **OP135502** | **OP134006** | **OP161556** | **This study** |
| ***Inosperma nivalellum*** | **MHNNU31689-1** | **China** | **OP389161** | **OP389202** | **OP407841** | **This study** |
| *Inosperma proximum* | ZT13015 | Thailand | EU600839 | EU600840 | N/A | Matheny et al. (2019) |
| *Inosperma quietiodor* | EL115_04 | Sweden | AM882960 | AM882960 | N/A | Pradeep et al. (2016) |
| *Inosperma cf. reisneri* | MCA646 | Japan | N/A | EU555463 | N/A | Pradeep et al. (2016) |
| *Inosperma rhodiolum* | EL223_06 | France | FJ904175 | FJ904175 | N/A | Pradeep et al. (2016) |
| *Inosperma rimosoides* | PBM2459 | USA | DQ404391 | AY702014 | DQ385884 | Pradeep et al. (2016) |
| *Inosperma rosellicaulare* | ACAD:11618(T) | Canada | MT237482 | N/A | N/A | TYPE material |
| *Inosperma rubricosum* | PBM3784(T) | Australia | NR_152369 | NG_057260 | KM406230 | Pradeep et al. (2016) |
| *Inosperma saragum* | CAL1360(T) | India | KY440103 | NG_057285 | N/A | Latha and Manimohan (2017) |
| *Inosperma shawarense* | ASSW79A | Pakistan | KY616964 | KY616966 | N/A | Naseer et al. (2017) |
| *Inosperma sp.* | BB3233 | Zambia | JQ801415 | EU600885 | N/A | Pradeep et al. (2016) |
| *Inosperma sp.* | PC96013 | Zambia | JQ801383 | EU600883 | EU600882 | Pradeep et al. (2016) |
| *Inosperma sp.* | PC96073 | Zambia | JQ801417 | EU600870 | EU600869 | Pradeep et al. (2016) |
| ***Inosperma sphaerobulbosum*** | **MHNNU32266(T)** | **China** | **OP135501** | **OP134001** | **OP161559** | **This study** |
| ***Inosperma sphaerobulbosum*** | **MHNNU32266-1** | **China** | **OP389166** | **OP389205** | **OP407842** | **This study** |
| ***Inosperma squamulosobrunneum*** | **FYG2869** | **China** | **OP389200** | **OP389211** | **OP407843** | **This study** |
| ***Inosperma squamulosobrunneum*** | **MHNNU32162** | **China** | **OP135508** | **OP134005** | **OP161557** | **This study** |
| ***Inosperma squamulosobrunneum*** | **MHNNU32351** | **China** | **OP135507** | **OP134003** | **OP161561** | **This study** |
| ***Inosperma squamulosobrunneum*** | **MHNNU32359(T)** | **China** | **OP135499** | **OP134000** | **OP161562** | **This study** |
| ***Inosperma squamulosohinnuleum*** | **FYG2015388** | **China** | **OP389199** | **OP389209** | **OP407844** | **This study** |
| ***Inosperma squamulosohinnuleum*** | **MHNNU32195(T)** | **China** | **OP135500** | **OP134002** | **OP161558** | **This study** |
| ***Inosperma squamulosohinnuleum*** | **MHNNU32362** | **China** | **OP135503** | **OP134004** | **OP161563** | **This study** |
| *Inosperma subhirsutum* | JV11950 | Latvia | N/A | EU555452 | AY333763 | Pradeep et al. (2016) |
| *Inosperma subsphaerosproum* | FYG5848 (T) | China | MW403825 | MW397171 | MW404237 | Deng et al. (2021b) |
| *Inosperma vinaceobrunneum* | PBM2951 | USA | N/A | HQ201353 | JQ846478 | Pradeep et al. (2016) |
| *Inosperma vinaceum* | AMB18747 | Italy | MW561108 | MW561120 | N/A | Cervini et al. (2021) |
| *Inosperma viridipes* | PBM3767(T) | Australia | NR_153168 | KP171094 | KM656138 | TYPE material |
| *Inosperma virosum* | TBGT753(T) | India | KT329452 | KT329458 | KT329446 | Pradeep et al. (2016)) |
| *Inosperma zonativeliferum* | FYG6441 | China | OL850878 | OM845772 | ON075044 | Deng et al. (2022) |
| *OUTGROUPS* |  |  |  |  |  |  |
| *Auritella dolichocystis* | Trappe24838(T) | Australia | N/A | NG_075155 | AY635767 | TYPE material |
| *Auritella dolichocystis* | Trappe24844 | Australia | N/A | AY380371 | N/A | Matheny et al. (2019) |

The new sequences generated in this study are shown in bold

*N/A* Not available

*T* type specimen

**References**

Aïgnon, H.L., Jabeen, S., Naseer, A., Yorou, N.S., and Ryberg, M. (2021). Three new species of *Inosperma* (Agaricales, Inocybaceae) from Tropical Africa. *MycoKeys* 77**,** 97-116. doi: 10.3897/mycokeys.77.60084

Bandini, D., Oertel, B., and Eberhardt, U. (2021). Even more fibre-caps (2): Thirteen new species of the family Inocybaceae. *Mycologia Bavarica* 21**,** 27-98.

Cervini, M., Carbone, M., and Bizio, E. (2021). *Inosperma vinaceum*, una nuova specie distinta da *I. rhodiolum* e *I. adaequatum*. *Rivista di Micologia* 63**,** 215-241.

Deng, L.S., Kang, R., Zeng, N.K., Yu, W.J., Chang, Z., Xu, F., Deng, W.Q., Qi, L.L., Zhou, Y.L., and Fan, Y.G. (2021a). Two new *Inosperma* (Inocybaceae) species with unexpected muscarine contents from tropical China. *MycoKeys* 85**,** 87-108. doi: 10.3897/mycokeys.85.71957

Deng, L.S., Yu, W.J., Zeng, N.K., Liu, L.J., Liu, L.Y., and Fan, Y.G. (2021b). *Inosperma subsphaerosporum* (Inocybaceae), a new species from Hainan, tropical China. *Phytotaxa* 502**,** 169-178. doi: 10.11646/phytotaxa.502.2.5

Deng, L.S., Yu, W.J., Zeng, N.K., Zhang, Y.Z., Wu, X.P., Li, H.J., Xu, F., and Fan, Y.G. (2022). A New Muscarine-Containing *Inosperma* (Inocybaceae, Agaricales) Species Discovered From One Poisoning Incident Occurring in Tropical China. *Frontiers in Microbiology* 13, 923435. doi:10.3389/fmicb.2022.923435

Fan, Y.G., and Bau, T. (2018). Three new species of *Inocybe* sect. *Rimosae* from China. *Mycosystema* 37**,** 693-702.

Kosentka, P., Sprague, S.L., Ryberg, M., Gartz, J., May, A.L., Campagna, S.R., and Matheny, P.B. (2013). Evolution of the toxins muscarine and psilocybin in a family of mushroom-forming fungi. *Plos One* 8**,** e64646. doi: 10.1371/journal.pone.0064646

Latha, K., and Manimohan, P. (2017). *Inocybes of Kerala.* Calicut, India: SporePrint Books.

Matheny, P.B. (2005). Improving phylogenetic inference of mushrooms with RPB1 and RPB2 nucleotide sequences (*Inocybe*; Agaricales). *Molecular Phylogenetics and Evolution* 35**,** 1-20. doi: 10.1016/j.ympev.2004.11.014

Matheny, P.B., and Bougher, N.L. (2017). *Fungi of Australia: Inocybaceae.* Canberra, Australian: Australian Biological Resources Study.

Matheny, P.B., Hobbs, A.M., and Fernando, E.R. (2019). Genera of Inocybaceae: New skin for the old ceremony. *Mycologia* 112**,** 1-38. doi: 10.1080/00275514.2019.1668906

Naseer, A., Khalid, A.N., and Smith, M.E. (2017). *Inocybe shawarensis* sp. nov. in the Inosperma clade from Pakistan. *Mycotaxon* 132**,** 909-918. doi: 10.5248/132.909

Pradeep, C.K., Vrinda, K.B., and Varghese, S.P. (2016). New and noteworthy species of Inocybe (Agaricales) from tropical India. *Mycological Progress* 15**,** 1-25. doi: 10.1007/s11557-016-1174-z
