# Supplementary material for: Five new species of Inosperma from China: Morphological characteristics, phylogenetic analyses, and toxin detection
Source: Front Microbiol. 2022 Oct 31;13:1021583. doi: 10.3389/fmicb.2022.1021583 (PMC9659589; doi:10.3389/fmicb.2022.1021583)

#### **Analyte:** muscarine **(**174.200/57.100 Da**)**

| Data File | DataHunanshifandaxue 20220111.wiff | Result Table | Hunanshifandaxue 20220819-3.rdb |
| --- | --- | --- | --- |
| Acquisition Date | 1/11/2022 3:51:53 PM | Algorithm Used | MQL |
| Acquisition Method | 20180927.dam | Instrument Name | 4000 Q TRAP |
| Project | Muscarine and muscimol |  |  |

| Sample Name | Sample Type | Area (cps) | RT (min) | Target [Conc]. (ng/mL) | Calculated Conc. (ng/mL) |
| --- | --- | --- | --- | --- | --- |
| Std 5 ng_mL | Standard | 1.680e+04 | 1.27 | 5.00 | 4.83 |
| Std 10 ng_mL | Standard | 3.660e+04 | 1.29 | 10.0 | 10.5 |
| Std 20 ng_mL | Standard | 6.770e+04 | 1.28 | 20.0 | 19.4 |
| Std 50 ng_mL | Standard | 1.790e+05 | 1.25 | 50.0 | 51.3 |
| Std 100 ng_mL | Standard | 3.470e+05 | 1.28 | 100. | 99.0 |
| Solvent blank | Unknown | 2.650e+01 | 1.28 | N/A | 0.0442 |
| MHHNU32337 | Unknown | 1.360e+01 | 1.19 | N/A | 0.0405 |
| MHHNU33070 | Unknown | 5.680e+01 | 1.27 | N/A | 0.0528 |
| MHHNU32266 | Unknown | 3.810e+01 | 1.48 | N/A | 0.0475 |
| MHHNU32362 | Unknown | 2.890e+01 | 1.25 | N/A | 0.0449 |
| MHHNU32359 | Unknown | 2.050e+02 | 1.26 | N/A | 0.0951 |
| MHHNU32195 | Unknown | 3.920e+02 | 1.26 | N/A | 0.149 |
| MHHNU32162 | Unknown | 9.790e+01 | 1.26 | N/A | 0.0646 |
| MHHNU32351 | Unknown | 2.310e+01 | 1.36 | N/A | 0.0432 |
| MHHNU31689 | Unknown | 1.300e+05 | 1.27 | N/A | 37.1 |

| \| Std 5 ng_mL \| \| \| \| --- \| --- \| --- \| \|  \| \| \| \| RT (Exp. RT): \| 1.27 (1.28) min \| \| \| Calculated Conc: \| 4.83 ng/mL \| \| \| Area: \| 1.68e+004 \|  \| \| Sample Type: \| (Standard) \| \| | 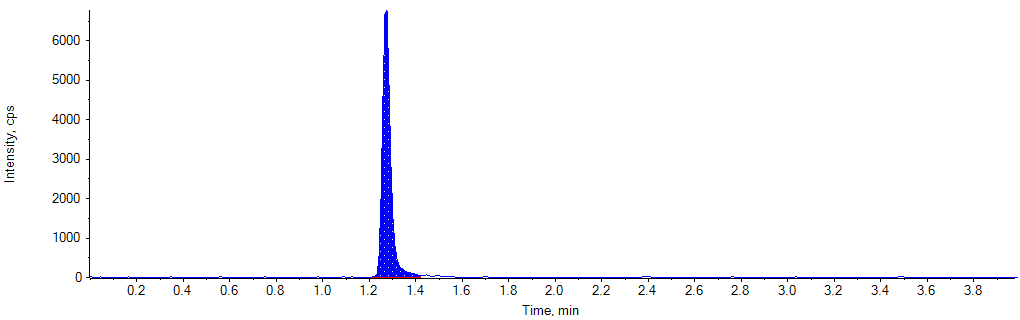 |
| --- | --- | --- | --- | --- | --- | --- | --- | --- | --- | --- | --- | --- | --- | --- | --- | --- | --- | --- | --- |

| \| Std 10 ng_mL \| \| \| \| --- \| --- \| --- \| \|  \| \| \| \| RT (Exp. RT): \| 1.29 (1.28) min \| \| \| Calculated Conc: \| 10.5 ng/mL \| \| \| Area: \| 3.66e+004 \|  \| \| Sample Type: \| (Standard) \| \| | 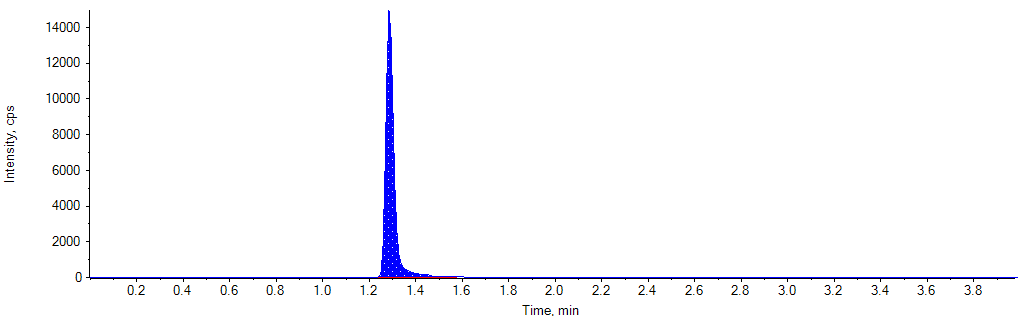 |
| --- | --- | --- | --- | --- | --- | --- | --- | --- | --- | --- | --- | --- | --- | --- | --- | --- | --- | --- | --- |

| \| Std 20 ng_mL \| \| \| \| --- \| --- \| --- \| \|  \| \| \| \| RT (Exp. RT): \| 1.28 (1.28) min \| \| \| Calculated Conc: \| 19.4 ng/mL \| \| \| Area: \| 6.77e+004 \|  \| \| Sample Type: \| (Standard) \| \| | 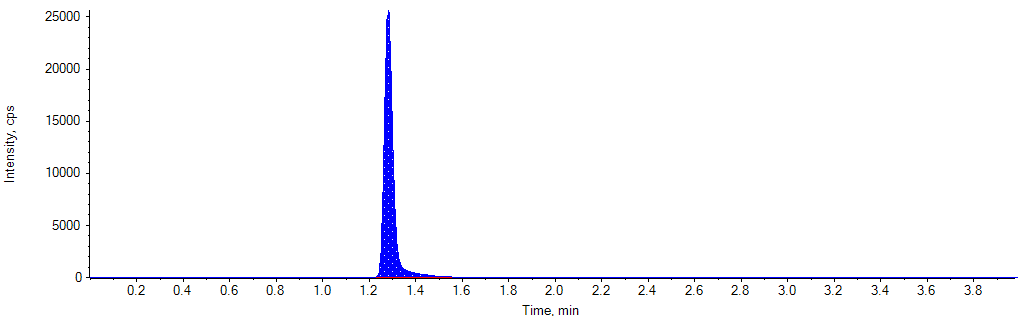 |
| --- | --- | --- | --- | --- | --- | --- | --- | --- | --- | --- | --- | --- | --- | --- | --- | --- | --- | --- | --- |

| \| Std 50 ng_mL \| \| \| \| --- \| --- \| --- \| \|  \| \| \| \| RT (Exp. RT): \| 1.25 (1.28) min \| \| \| Calculated Conc: \| 51.3 ng/mL \| \| \| Area: \| 1.79e+005 \|  \| \| Sample Type: \| (Standard) \| \| | 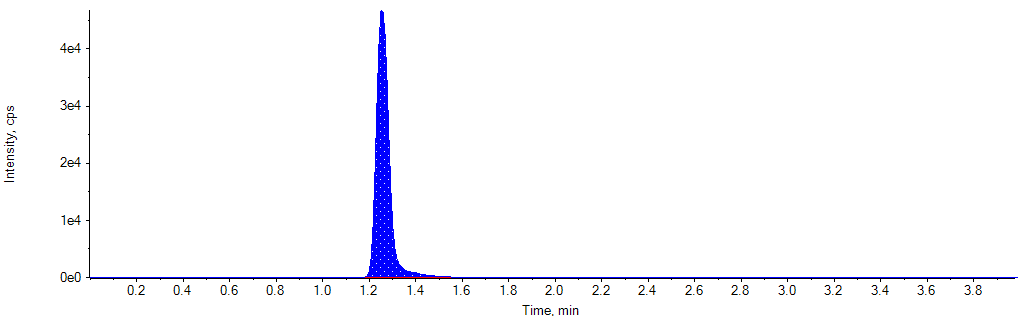 |
| --- | --- | --- | --- | --- | --- | --- | --- | --- | --- | --- | --- | --- | --- | --- | --- | --- | --- | --- | --- |

| \| Std 100 ng_mL \| \| \| \| --- \| --- \| --- \| \|  \| \| \| \| RT (Exp. RT): \| 1.28 (0.00) min \| \| \| Calculated Conc: \| 99.0 ng/mL \| \| \| Area: \| 3.47e+005 \|  \| \| Sample Type: \| (Standard) \| \| | 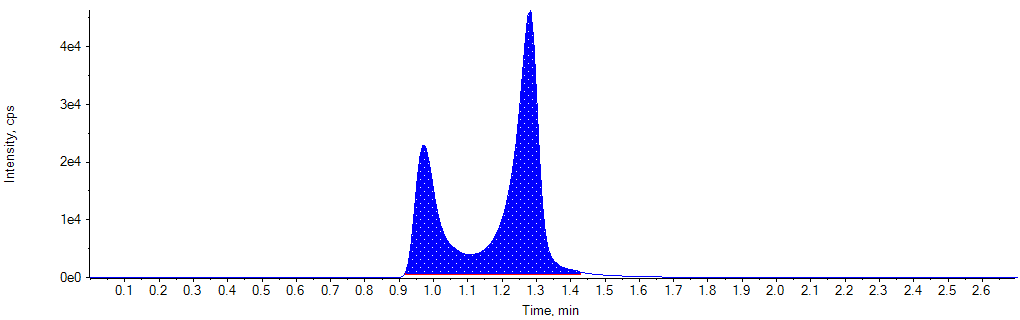 |
| --- | --- | --- | --- | --- | --- | --- | --- | --- | --- | --- | --- | --- | --- | --- | --- | --- | --- | --- | --- |

| \| Solvent blank \| \| \| \| --- \| --- \| --- \| \|  \| \| \| \| RT (Exp. RT): \| 1.28 (1.28) min \| \| \| Calculated Conc: \| 0.0442 ng/mL \| \| \| Area: \| 2.65e+001 \|  \| \| Sample Type: \| (Unknown) \| \| | 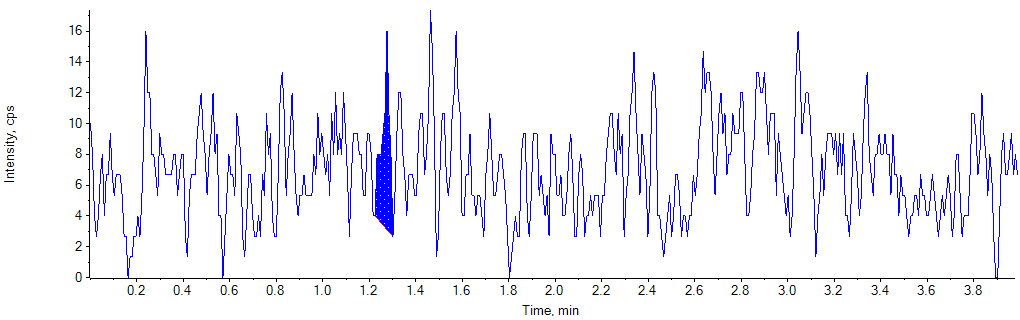 |
| --- | --- | --- | --- | --- | --- | --- | --- | --- | --- | --- | --- | --- | --- | --- | --- | --- | --- | --- | --- |

| \| MHHNU32337 \| \| \| \| --- \| --- \| --- \| \|  \| \| \| \| RT (Exp. RT): \| 1.19 (1.28) min \| \| \| Calculated Conc: \| 0.0405 ng/mL \| \| \| Area: \| 1.36e+001 \|  \| \| Sample Type: \| (Unknown) \| \| | 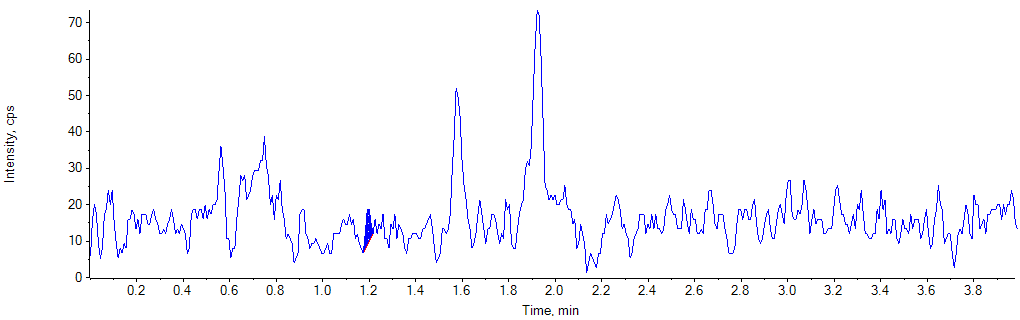 |
| --- | --- | --- | --- | --- | --- | --- | --- | --- | --- | --- | --- | --- | --- | --- | --- | --- | --- | --- | --- |

| \| MHHNU33070 \| \| \| \| --- \| --- \| --- \| \|  \| \| \| \| RT (Exp. RT): \| 1.27 (1.28) min \| \| \| Calculated Conc: \| 0.0528 ng/mL \| \| \| Area: \| 5.68e+001 \|  \| \| Sample Type: \| (Unknown) \| \| | 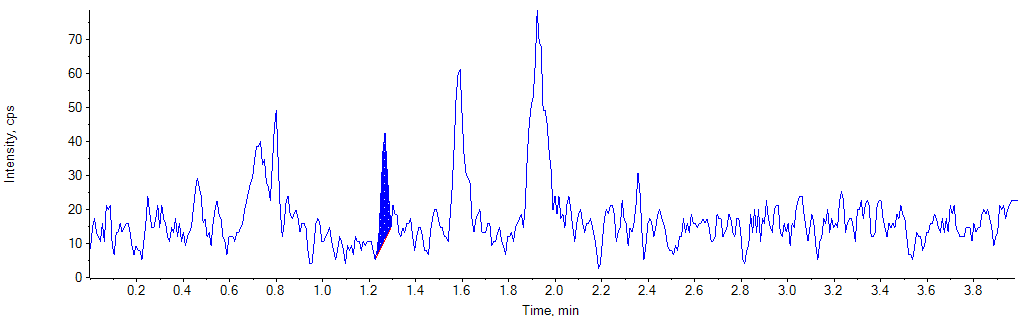 |
| --- | --- | --- | --- | --- | --- | --- | --- | --- | --- | --- | --- | --- | --- | --- | --- | --- | --- | --- | --- |

| \| MHHNU32266 \| \| \| \| --- \| --- \| --- \| \|  \| \| \| \| RT (Exp. RT): \| 1.48 (1.28) min \| \| \| Calculated Conc: \| 0.0475 ng/mL \| \| \| Area: \| 3.81e+001 \|  \| \| Sample Type: \| (Unknown) \| \| | 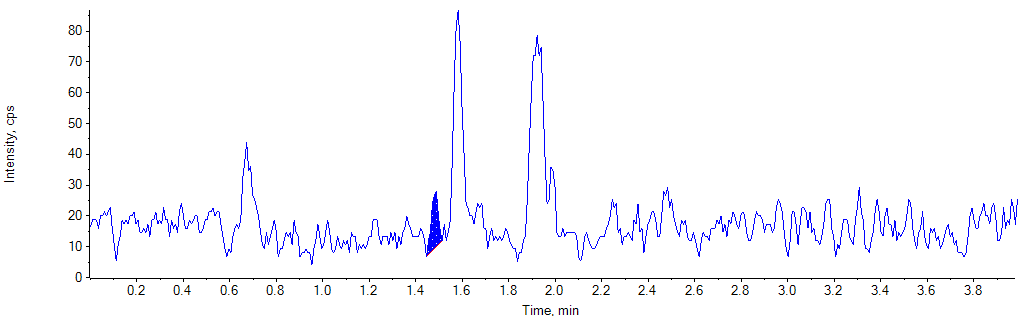 |
| --- | --- | --- | --- | --- | --- | --- | --- | --- | --- | --- | --- | --- | --- | --- | --- | --- | --- | --- | --- |

| \| MHHNU32362 \| \| \| \| --- \| --- \| --- \| \|  \| \| \| \| RT (Exp. RT): \| 1.25 (1.28) min \| \| \| Calculated Conc: \| 0.0449 ng/mL \| \| \| Area: \| 2.89e+001 \|  \| \| Sample Type: \| (Unknown) \| \| | 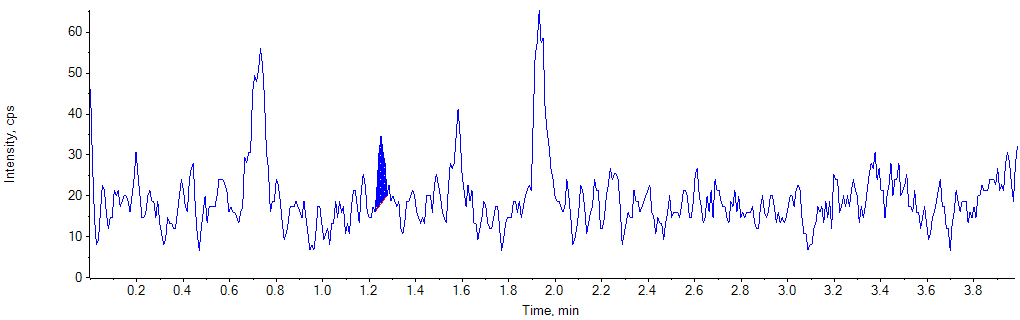 |
| --- | --- | --- | --- | --- | --- | --- | --- | --- | --- | --- | --- | --- | --- | --- | --- | --- | --- | --- | --- |

| \| MHHNU32359 \| \| \| \| --- \| --- \| --- \| \|  \| \| \| \| RT (Exp. RT): \| 1.26 (1.28) min \| \| \| Calculated Conc: \| 0.0951 ng/mL \| \| \| Area: \| 2.05e+002 \|  \| \| Sample Type: \| (Unknown) \| \| | 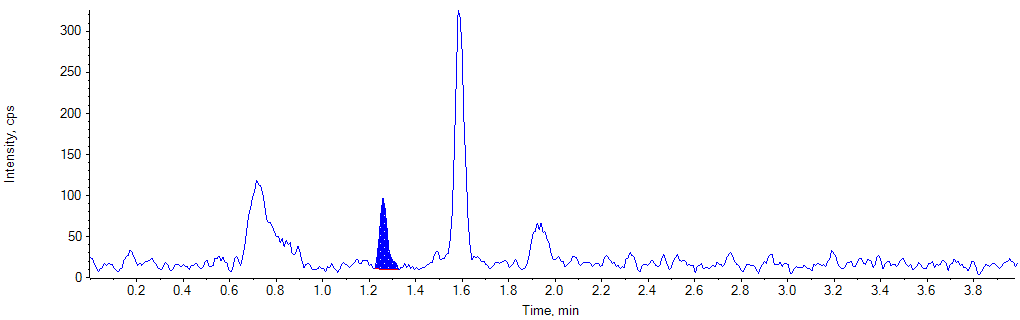 |
| --- | --- | --- | --- | --- | --- | --- | --- | --- | --- | --- | --- | --- | --- | --- | --- | --- | --- | --- | --- |

| \| MHHNU32195 \| \| \| \| --- \| --- \| --- \| \|  \| \| \| \| RT (Exp. RT): \| 1.26 (1.28) min \| \| \| Calculated Conc: \| 0.149 ng/mL \| \| \| Area: \| 3.92e+002 \|  \| \| Sample Type: \| (Unknown) \| \| | 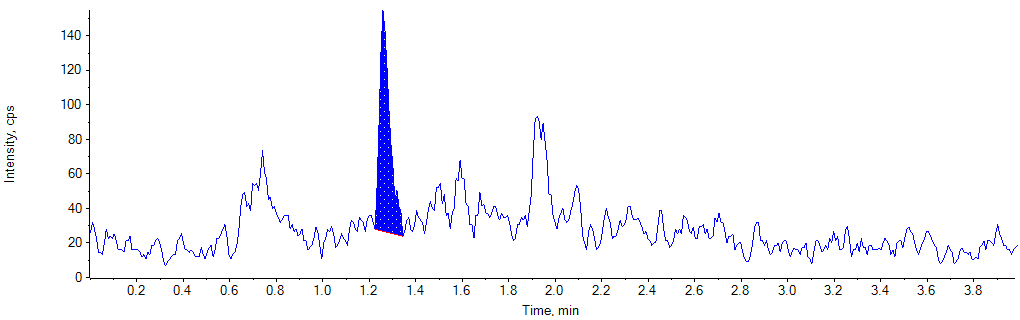 |
| --- | --- | --- | --- | --- | --- | --- | --- | --- | --- | --- | --- | --- | --- | --- | --- | --- | --- | --- | --- |

| \| MHHNU32162 \| \| \| \| --- \| --- \| --- \| \|  \| \| \| \| RT (Exp. RT): \| 1.26 (1.28) min \| \| \| Calculated Conc: \| 0.0646 ng/mL \| \| \| Area: \| 9.79e+001 \|  \| \| Sample Type: \| (Unknown) \| \| | 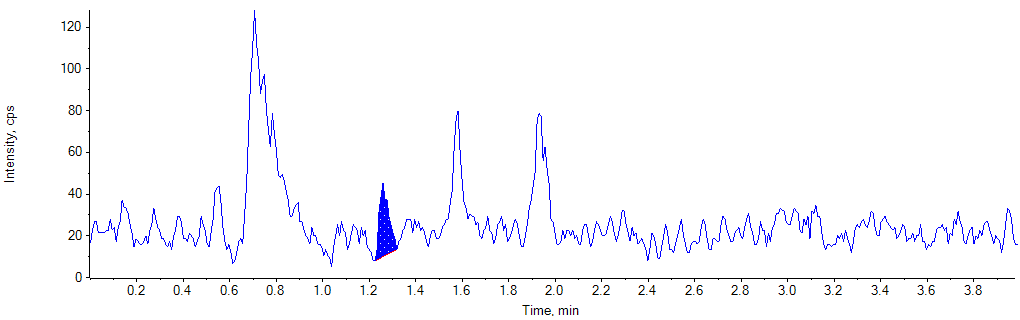 |
| --- | --- | --- | --- | --- | --- | --- | --- | --- | --- | --- | --- | --- | --- | --- | --- | --- | --- | --- | --- |

| \| MHHNU32351 \| \| \| \| --- \| --- \| --- \| \|  \| \| \| \| RT (Exp. RT): \| 1.36 (1.28) min \| \| \| Calculated Conc: \| 0.0432 ng/mL \| \| \| Area: \| 2.31e+001 \|  \| \| Sample Type: \| (Unknown) \| \| | 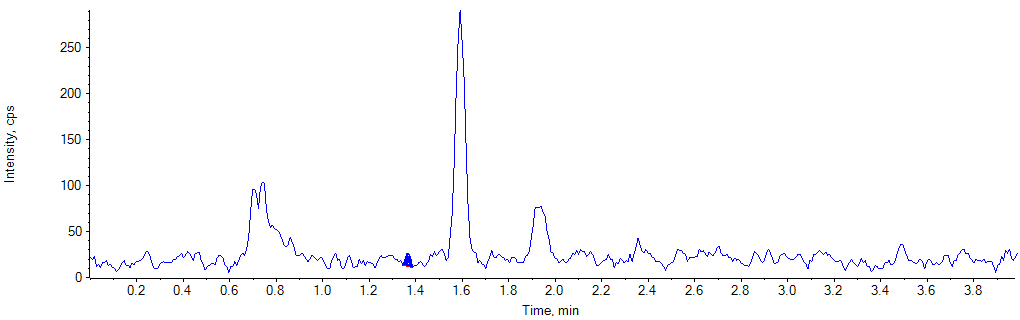 |
| --- | --- | --- | --- | --- | --- | --- | --- | --- | --- | --- | --- | --- | --- | --- | --- | --- | --- | --- | --- |

| \| MHHNU31689 \| \| \| \| --- \| --- \| --- \| \|  \| \| \| \| RT (Exp. RT): \| 1.27 (1.28) min \| \| \| Calculated Conc: \| 37.1 ng/mL \| \| \| Area: \| 1.30e+005 \|  \| \| Sample Type: \| (Unknown) \| \| | 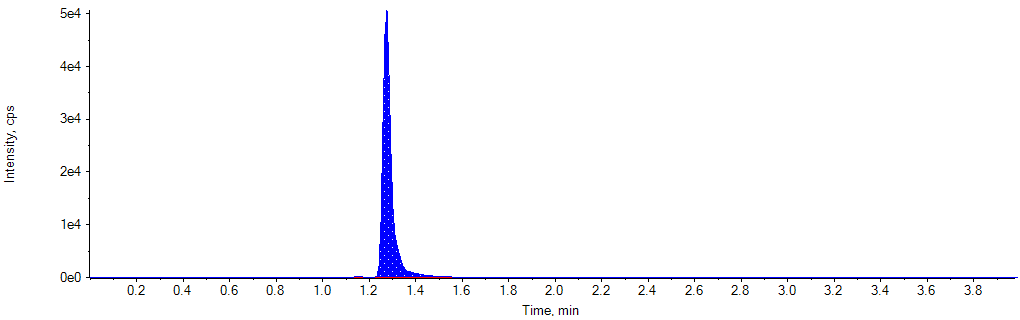 |
| --- | --- | --- | --- | --- | --- | --- | --- | --- | --- | --- | --- | --- | --- | --- | --- | --- | --- | --- | --- |

####

#### **Analyte Name:** muscarine

#### Internal Standard: *No data for IS Peak Name*

####

| Data File | DataHunanshifandaxue 20220111.wiff | Result Table | Hunanshifandaxue 20220819-3.rdb |
| --- | --- | --- | --- |
| Acquisition Date | 1/11/2022 3:51:53 PM | Algorithm Used | MQL |
| Acquisition Method | 20180927.dam | Instrument Name | 4000 Q TRAP |
| Project | Muscarine and muscimol |  |  |

#### Regression Equation: y = 3.5e+003 x + -128 (r = 0.9996)

| Expected Concentration | Number of Values | MeanCalculated Concentration | % Accuracy | Std. Deviation | %CV | |
| --- | --- | --- | --- | --- | --- | --- |
| 5 | 1 | 4.83 | 96.6 | NaN | | NaN |
| 10 | 1 | 10.50 | 105.0 | NaN | | NaN |
| 20 | 1 | 19.36 | 96.8 | NaN | | NaN |
| 50 | 1 | 51.29 | 102.6 | NaN | | NaN |
| 100 | 1 | 99.03 | 99.0 | NaN | | NaN |


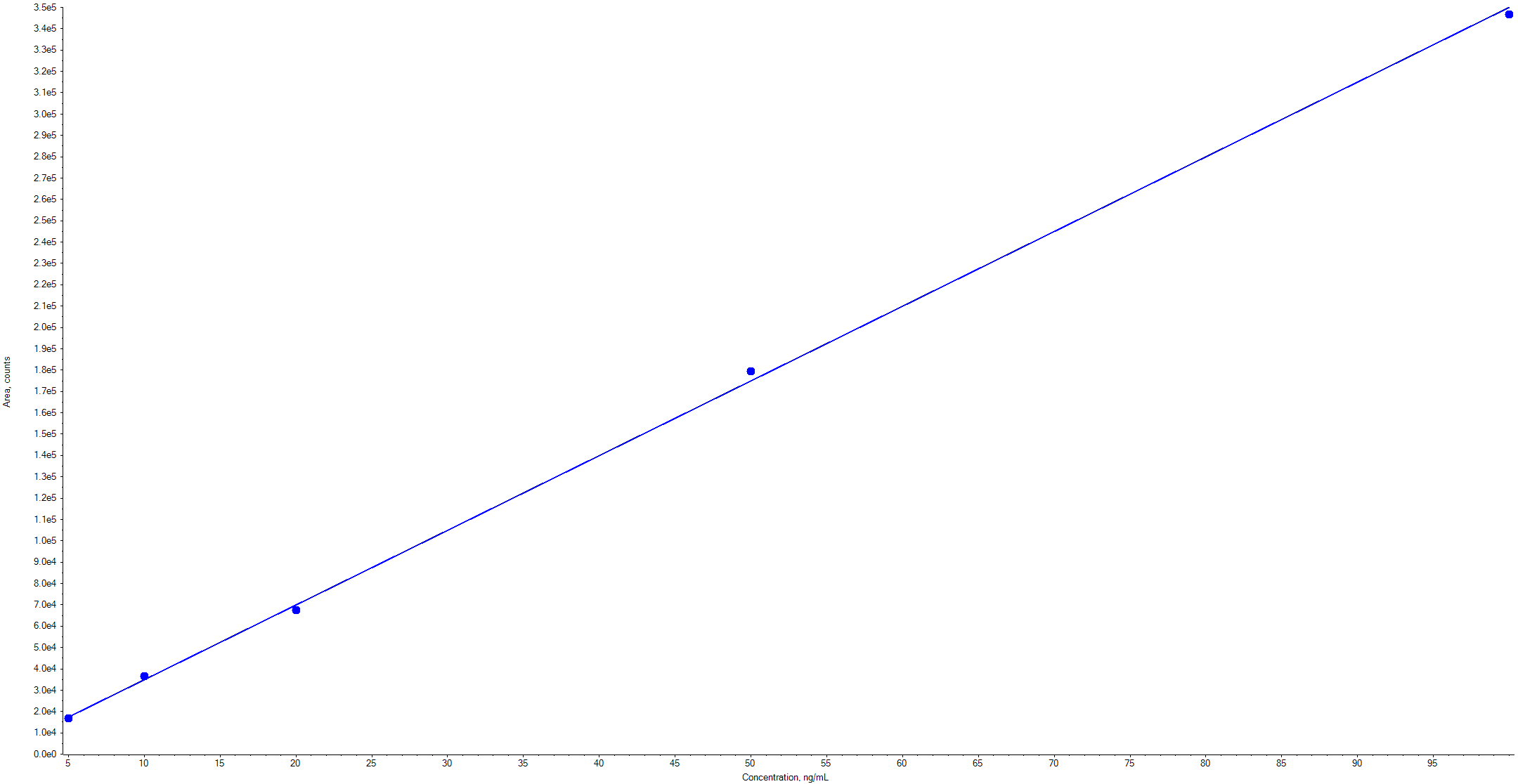


#### **Analyte:** Muscimol **(**115.100/98.000 Da**)**

| Data File | DataHunanshifandaxue 20210112.wiff | Result Table | Hunanshifandaxue 20220819-2.rdb |
| --- | --- | --- | --- |
| Acquisition Date | 1/14/2022 12:31:35 PM | Algorithm Used | MQL |
| Acquisition Method | 20180927-1.dam | Instrument Name | 4000 Q TRAP |
| Project | Muscarine and muscimol |  |  |

| Sample Name | Sample Type | Area (cps) | RT (min) | Target [Conc]. (ng/mL) | Calculated Conc. (ng/mL) |
| --- | --- | --- | --- | --- | --- |
| NH3 Std 1 ng_mL | Standard | 1.530e+04 | 0.840 | 1.00 | 0.815 |
| NH3 Std 2 ng_mL | Standard | 2.930e+04 | 0.840 | 2.00 | 2.01 |
| NH3 Std 5 ng_mL | Standard | 7.070e+04 | 0.838 | 5.00 | 5.55 |
| NH3 Std 10 ng_mL | Standard | 1.430e+05 | 0.834 | 10.0 | 11.7 |
| NH3 Std 20 ng_mL | Standard | 2.150e+05 | 0.836 | 20.0 | 17.9 |
| Solvent blank | Unknown | 1.230e+02 | 0.717 | N/A | N/A |
| MHHNU32337 | Unknown | 6.880e+03 | 0.870 | N/A | 0.100 |
| MHHNU33070 | Unknown | 2.090e+04 | 0.860 | N/A | 1.29 |
| MHHNU32266 | Unknown | 4.370e+03 | 0.908 | N/A | N/A |
| MHHNU31689 | Unknown | 2.590e+03 | 0.866 | N/A | N/A |
| MHHNU32362 | Unknown | 2.510e+04 | 0.853 | N/A | 1.65 |
| MHHNU32359 | Unknown | 1.370e+05 | 0.852 | N/A | 11.2 |
| MHHNU32195 | Unknown | 4.330e+03 | 0.849 | N/A | N/A |
| MHHNU32162 | Unknown | 1.080e+05 | 0.849 | N/A | 8.71 |
| MHHNU32351 | Unknown | 7.510e+04 | 0.852 | N/A | 5.93 |

| \| NH3 Std 1 ng_mL \| \| \| \| --- \| --- \| --- \| \|  \| \| \| \| RT (Exp. RT): \| 0.840 (0.836) min \| \| \| Calculated Conc: \| 0.815 ng/mL \| \| \| Area: \| 1.53e+004 \|  \| \| Sample Type: \| (Standard) \| \| | 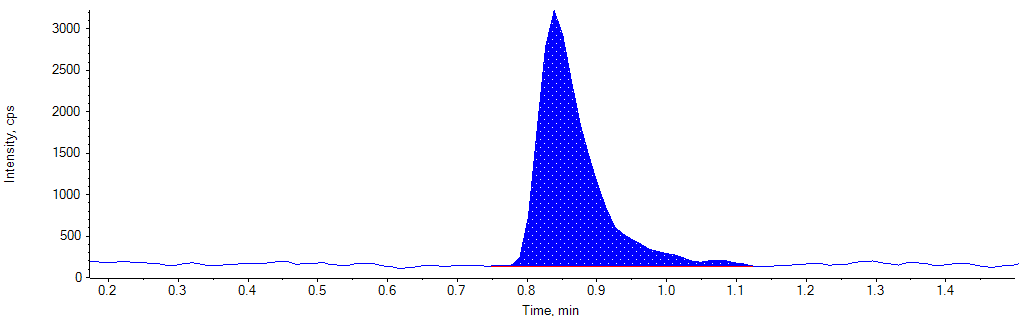 |
| --- | --- | --- | --- | --- | --- | --- | --- | --- | --- | --- | --- | --- | --- | --- | --- | --- | --- | --- | --- |

| \| NH3 Std 2 ng_mL \| \| \| \| --- \| --- \| --- \| \|  \| \| \| \| RT (Exp. RT): \| 0.840 (0.836) min \| \| \| Calculated Conc: \| 2.01 ng/mL \| \| \| Area: \| 2.93e+004 \|  \| \| Sample Type: \| (Standard) \| \| | 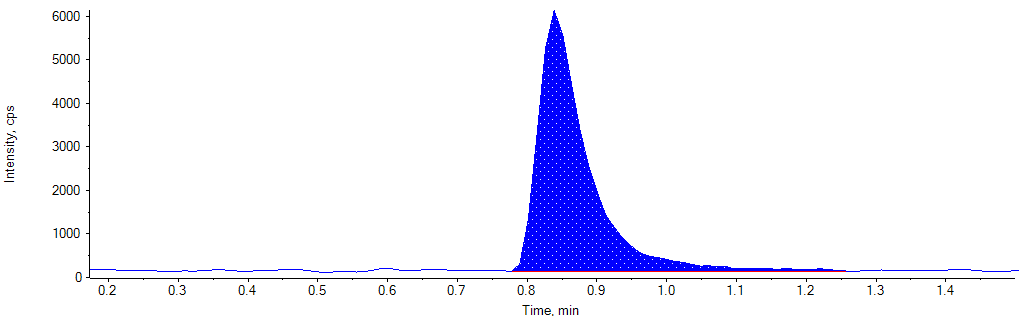 |
| --- | --- | --- | --- | --- | --- | --- | --- | --- | --- | --- | --- | --- | --- | --- | --- | --- | --- | --- | --- |

| \| NH3 Std 5 ng_mL \| \| \| \| --- \| --- \| --- \| \|  \| \| \| \| RT (Exp. RT): \| 0.838 (0.836) min \| \| \| Calculated Conc: \| 5.55 ng/mL \| \| \| Area: \| 7.07e+004 \|  \| \| Sample Type: \| (Standard) \| \| | 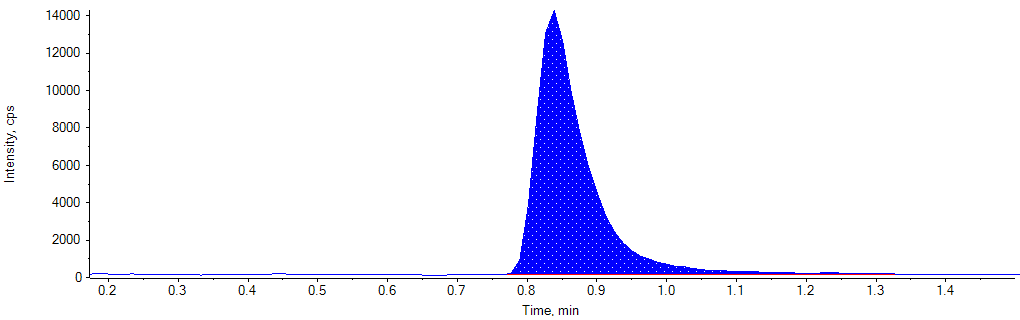 |
| --- | --- | --- | --- | --- | --- | --- | --- | --- | --- | --- | --- | --- | --- | --- | --- | --- | --- | --- | --- |

| \| NH3 Std 10 ng_mL \| \| \| \| --- \| --- \| --- \| \|  \| \| \| \| RT (Exp. RT): \| 0.834 (0.836) min \| \| \| Calculated Conc: \| 11.7 ng/mL \| \| \| Area: \| 1.43e+005 \|  \| \| Sample Type: \| (Standard) \| \| | 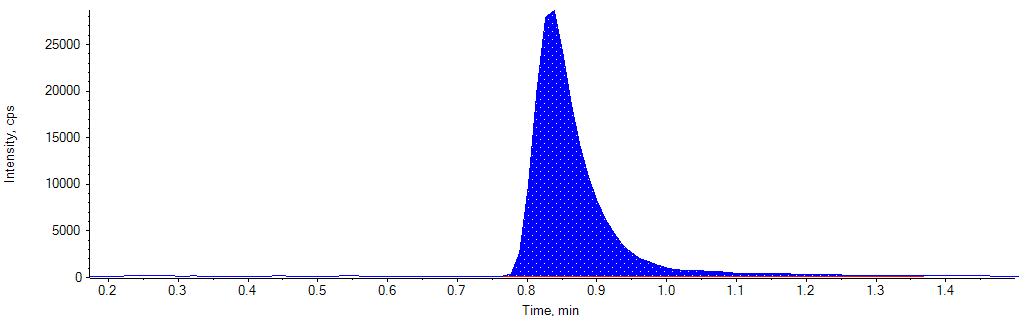 |
| --- | --- | --- | --- | --- | --- | --- | --- | --- | --- | --- | --- | --- | --- | --- | --- | --- | --- | --- | --- |

| \| NH3 Std 20 ng_mL \| \| \| \| --- \| --- \| --- \| \|  \| \| \| \| RT (Exp. RT): \| 0.836 (0.836) min \| \| \| Calculated Conc: \| 17.9 ng/mL \| \| \| Area: \| 2.15e+005 \|  \| \| Sample Type: \| (Standard) \| \| | 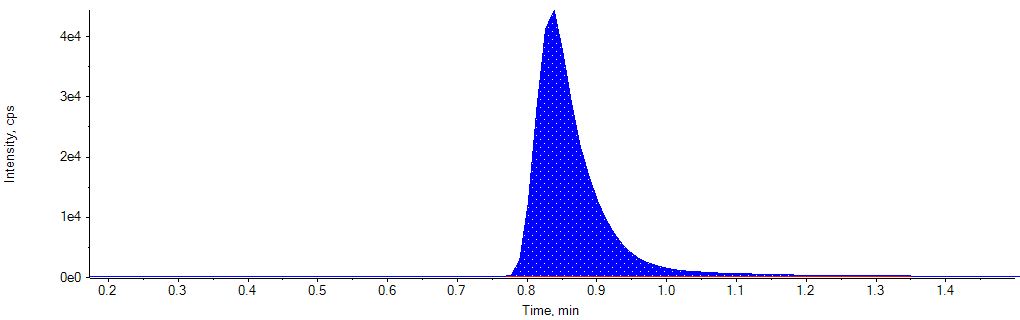 |
| --- | --- | --- | --- | --- | --- | --- | --- | --- | --- | --- | --- | --- | --- | --- | --- | --- | --- | --- | --- |

| \| Solvent blank \| \| \| \| --- \| --- \| --- \| \|  \| \| \| \| RT (Exp. RT): \| 0.717 (0.836) min \| \| \| Calculated Conc: \| N/A ng/mL \| \| \| Area: \| 1.23e+002 \|  \| \| Sample Type: \| (Unknown) \| \| | 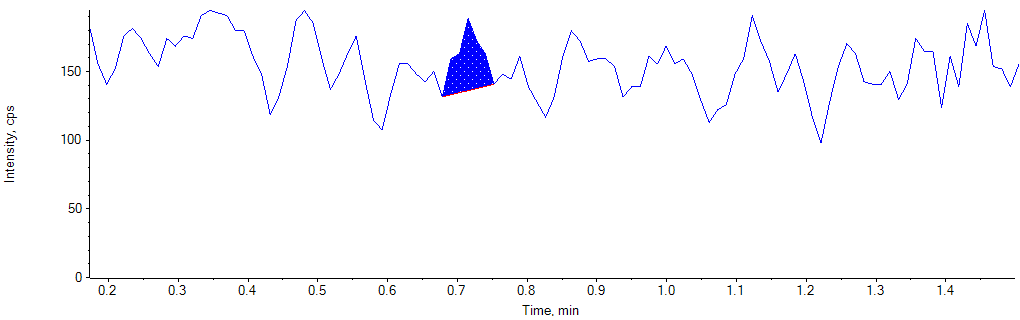 |
| --- | --- | --- | --- | --- | --- | --- | --- | --- | --- | --- | --- | --- | --- | --- | --- | --- | --- | --- | --- |

| \| MHHNU32337 \| \| \| \| --- \| --- \| --- \| \|  \| \| \| \| RT (Exp. RT): \| 0.870 (0.836) min \| \| \| Calculated Conc: \| 0.100 ng/mL \| \| \| Area: \| 6.88e+003 \|  \| \| Sample Type: \| (Unknown) \| \| | 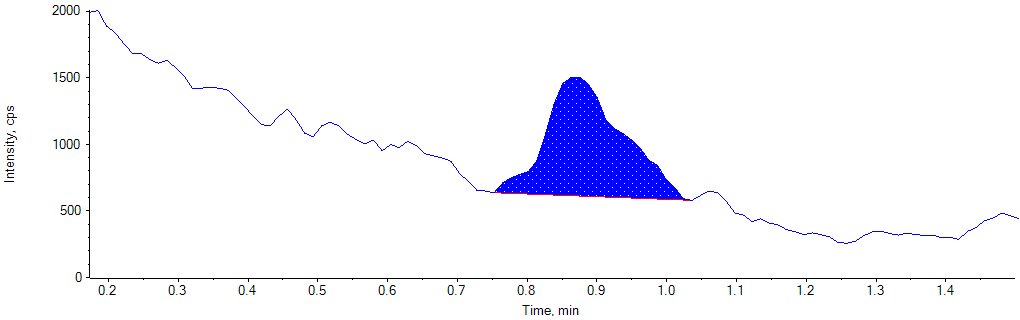 |
| --- | --- | --- | --- | --- | --- | --- | --- | --- | --- | --- | --- | --- | --- | --- | --- | --- | --- | --- | --- |

| \| MHHNU33070 \| \| \| \| --- \| --- \| --- \| \|  \| \| \| \| RT (Exp. RT): \| 0.860 (0.836) min \| \| \| Calculated Conc: \| 1.29 ng/mL \| \| \| Area: \| 2.09e+004 \|  \| \| Sample Type: \| (Unknown) \| \| | 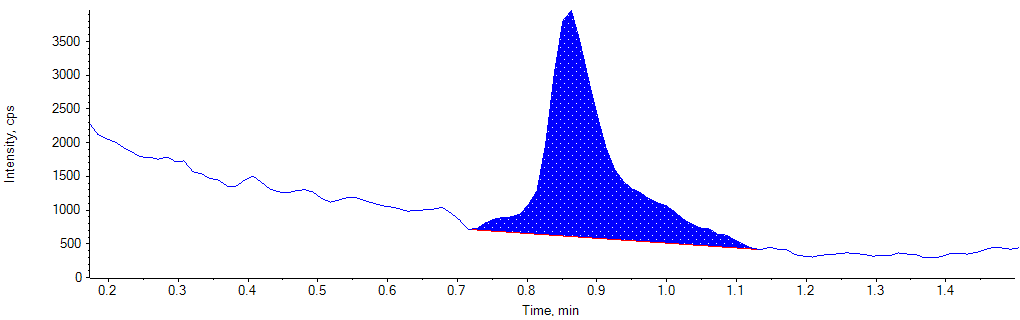 |
| --- | --- | --- | --- | --- | --- | --- | --- | --- | --- | --- | --- | --- | --- | --- | --- | --- | --- | --- | --- |

| \| MHHNU32266 \| \| \| \| --- \| --- \| --- \| \|  \| \| \| \| RT (Exp. RT): \| 0.908 (0.836) min \| \| \| Calculated Conc: \| N/A ng/mL \| \| \| Area: \| 4.37e+003 \|  \| \| Sample Type: \| (Unknown) \| \| | 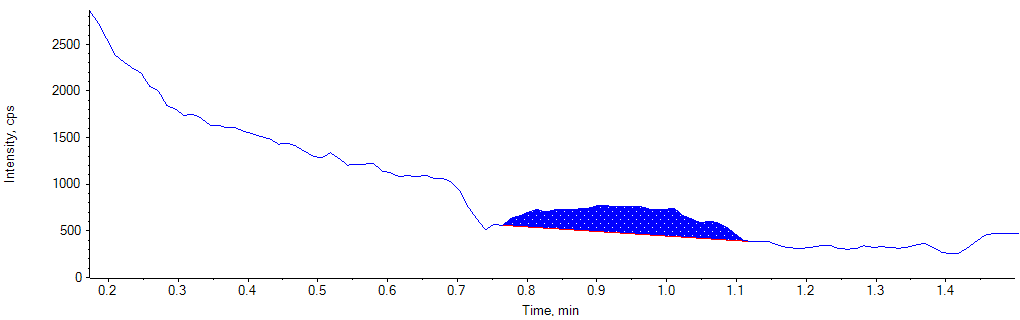 |
| --- | --- | --- | --- | --- | --- | --- | --- | --- | --- | --- | --- | --- | --- | --- | --- | --- | --- | --- | --- |

| \| MHHNU31689 \| \| \| \| --- \| --- \| --- \| \|  \| \| \| \| RT (Exp. RT): \| 0.866 (0.836) min \| \| \| Calculated Conc: \| N/A ng/mL \| \| \| Area: \| 2.59e+003 \|  \| \| Sample Type: \| (Unknown) \| \| | 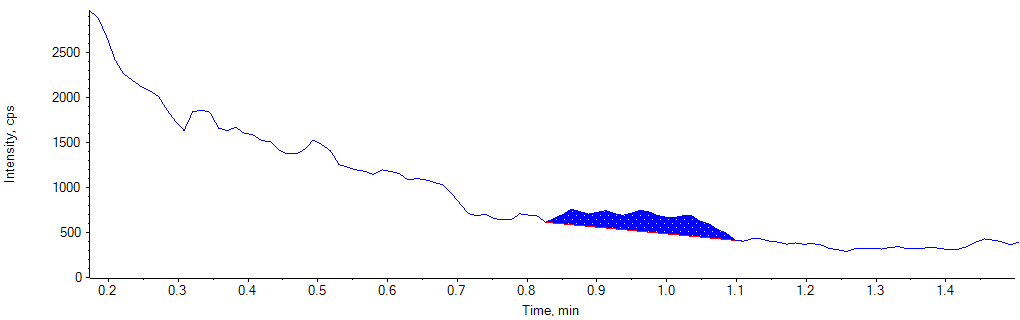 |
| --- | --- | --- | --- | --- | --- | --- | --- | --- | --- | --- | --- | --- | --- | --- | --- | --- | --- | --- | --- |

| \| MHHNU32362 \| \| \| \| --- \| --- \| --- \| \|  \| \| \| \| RT (Exp. RT): \| 0.853 (0.836) min \| \| \| Calculated Conc: \| 1.65 ng/mL \| \| \| Area: \| 2.51e+004 \|  \| \| Sample Type: \| (Unknown) \| \| | 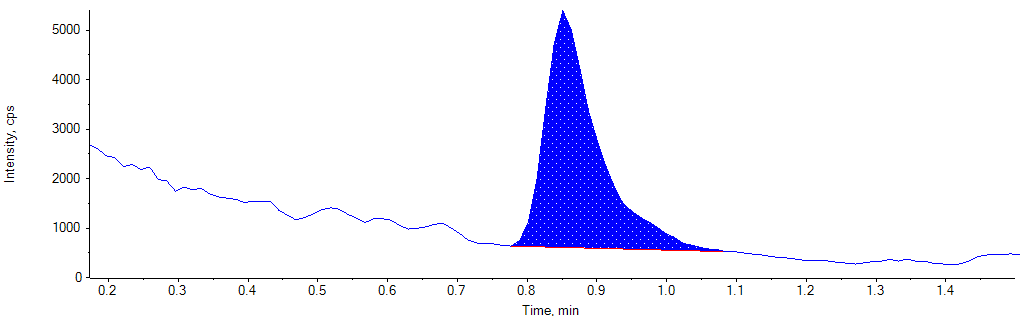 |
| --- | --- | --- | --- | --- | --- | --- | --- | --- | --- | --- | --- | --- | --- | --- | --- | --- | --- | --- | --- |

| \| MHHNU32359 \| \| \| \| --- \| --- \| --- \| \|  \| \| \| \| RT (Exp. RT): \| 0.852 (0.836) min \| \| \| Calculated Conc: \| 11.2 ng/mL \| \| \| Area: \| 1.37e+005 \|  \| \| Sample Type: \| (Unknown) \| \| | 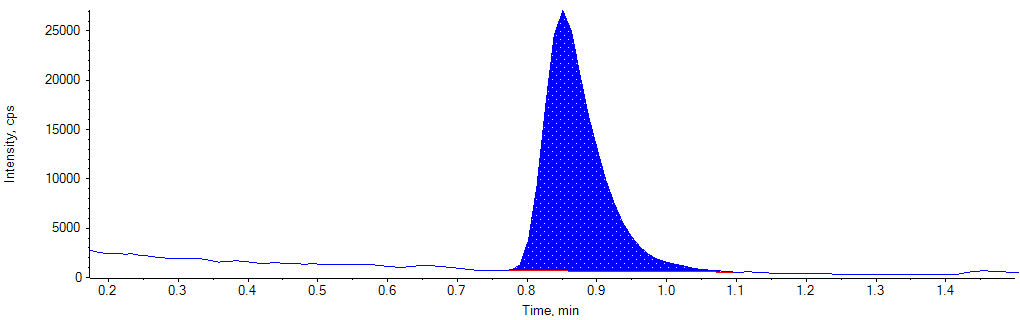 |
| --- | --- | --- | --- | --- | --- | --- | --- | --- | --- | --- | --- | --- | --- | --- | --- | --- | --- | --- | --- |

| \| MHHNU32195 \| \| \| \| --- \| --- \| --- \| \|  \| \| \| \| RT (Exp. RT): \| 0.849 (0.836) min \| \| \| Calculated Conc: \| N/A ng/mL \| \| \| Area: \| 4.33e+003 \|  \| \| Sample Type: \| (Unknown) \| \| | 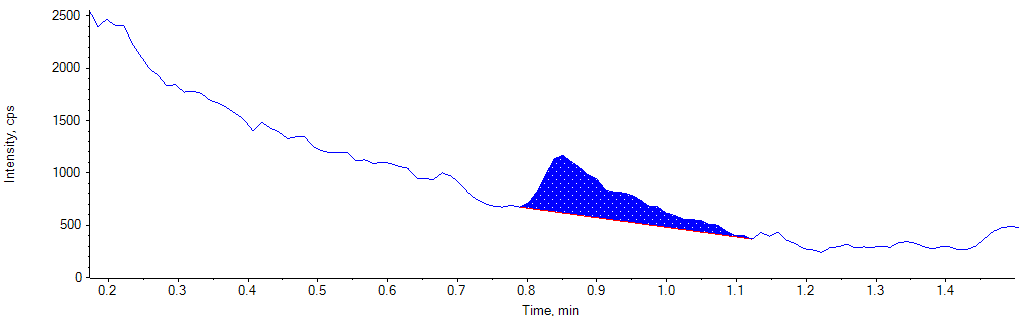 |
| --- | --- | --- | --- | --- | --- | --- | --- | --- | --- | --- | --- | --- | --- | --- | --- | --- | --- | --- | --- |

| \| MHHNU32162 \| \| \| \| --- \| --- \| --- \| \|  \| \| \| \| RT (Exp. RT): \| 0.849 (0.836) min \| \| \| Calculated Conc: \| 8.71 ng/mL \| \| \| Area: \| 1.08e+005 \|  \| \| Sample Type: \| (Unknown) \| \| | 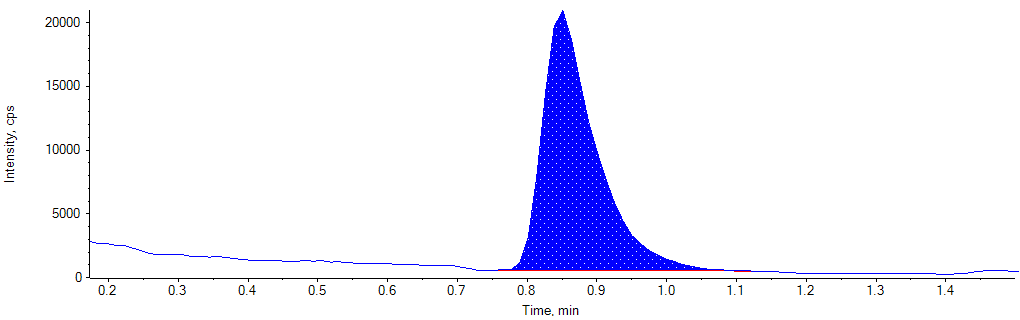 |
| --- | --- | --- | --- | --- | --- | --- | --- | --- | --- | --- | --- | --- | --- | --- | --- | --- | --- | --- | --- |

| \| MHHNU32351 \| \| \| \| --- \| --- \| --- \| \|  \| \| \| \| RT (Exp. RT): \| 0.852 (0.836) min \| \| \| Calculated Conc: \| 5.93 ng/mL \| \| \| Area: \| 7.51e+004 \|  \| \| Sample Type: \| (Unknown) \| \| | 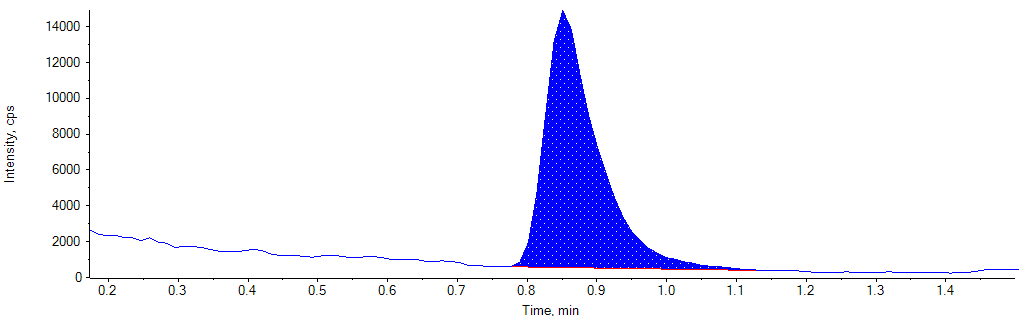 |
| --- | --- | --- | --- | --- | --- | --- | --- | --- | --- | --- | --- | --- | --- | --- | --- | --- | --- | --- | --- |

#### **Analyte Name:** Muscimol

#### Internal Standard: *No data for IS Peak Name*

####

| Data File | DataHunanshifandaxue 20210112.wiff | Result Table | Hunanshifandaxue 20220819-2.rdb |
| --- | --- | --- | --- |
| Acquisition Date | 1/14/2022 12:31:35 PM | Algorithm Used | MQL |
| Acquisition Method | 20180927-1.dam | Instrument Name | 4000 Q TRAP |
| Project | Muscarine and muscimol |  |  |

#### Regression Equation: y = 1.12e+004 x + 6.21e+003 (r = 0.9944)

| Expected Concentration | Number of Values | MeanCalculated Concentration | % Accuracy | Std. Deviation | %CV | |
| --- | --- | --- | --- | --- | --- | --- |
| 1 | 1 | 0.81 | 81.1 | NaN | | NaN |
| 2 | 1 | 2.07 | 103.5 | NaN | | NaN |
| 5 | 1 | 5.78 | 115.7 | NaN | | NaN |
| 10 | 1 | 10.61 | 106.1 | NaN | | NaN |
| 20 | 1 | 18.73 | 93.6 | NaN | | NaN |


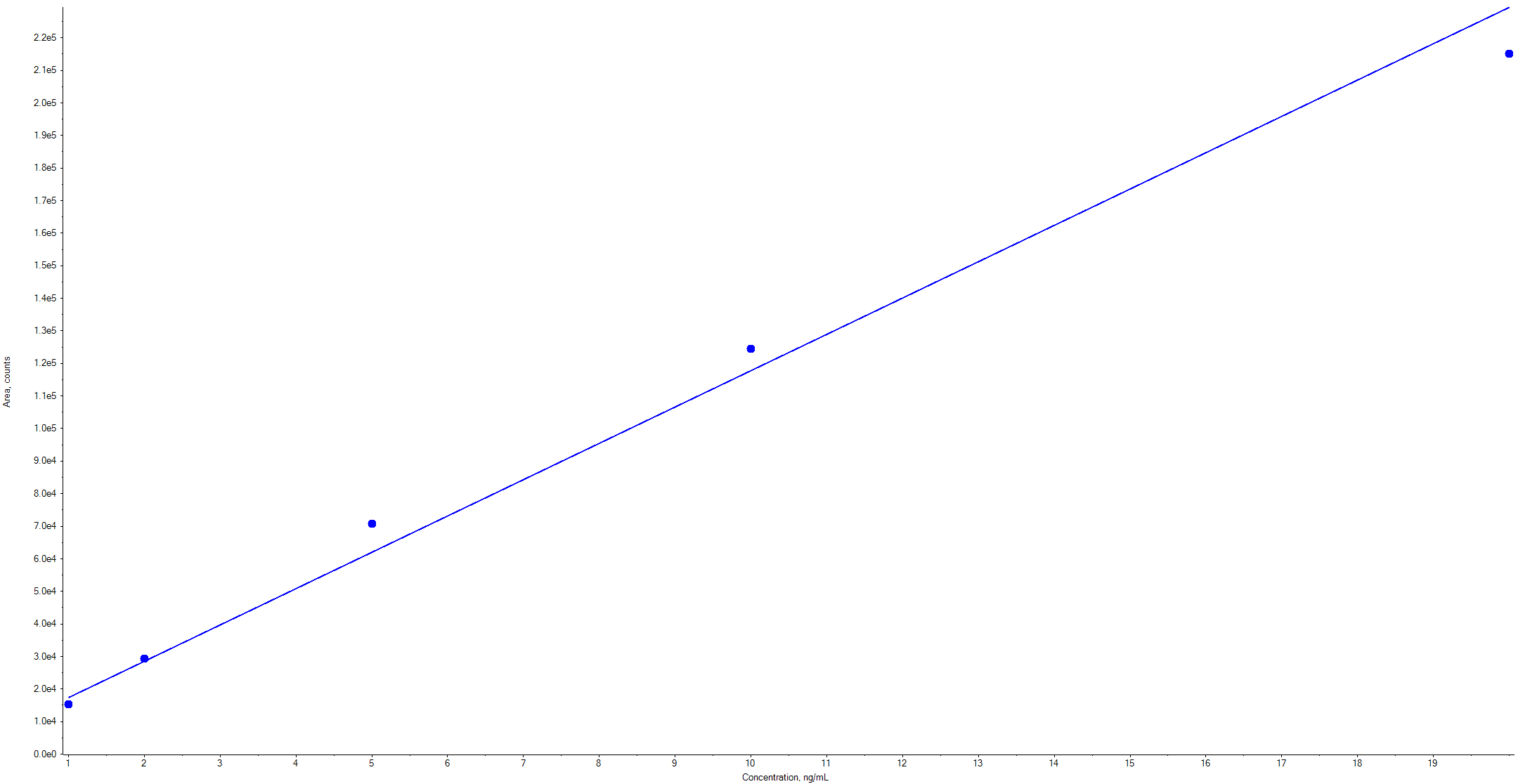


#### **Analyte:** ibotenic acid **(**158.900/113.200 Da**)**

| Data File | DataHunanshifandaxue 20210112.wiff | Result Table | Hunanshifandaxue 20220819-1.rdb |
| --- | --- | --- | --- |
| Acquisition Date | 1/14/2022 1:16:48 PM | Algorithm Used | MQL |
| Acquisition Method | 20180927-1.dam | Instrument Name | 4000 Q TRAP |
| Project | Muscarine and muscimol |  |  |

| Sample Name | Sample Type | Area (cps) | RT (min) | Target [Conc]. (ng/mL) | Calculated Conc. (ng/mL) |
| --- | --- | --- | --- | --- | --- |
| COO Std 1 ng_mL | Standard | 2.400e+04 | 0.865 | 1.00 | 1.13 |
| COO Std 2 ng_mL | Standard | 3.630e+04 | 0.868 | 2.00 | 1.66 |
| COO Std 5 ng_mL | Standard | 1.160e+05 | 0.865 | 5.00 | 5.12 |
| COO Std 10 ng_mL | Standard | 2.320e+05 | 0.867 | 10.0 | 10.2 |
| COO Std 20 ng_mL | Standard | 4.580e+05 | 0.865 | 20.0 | 19.9 |
| Solvent blank | Unknown | 9.770e+02 | 0.666 | N/A | 0.136 |
| MHHNU32337 | Unknown | 1.150e+03 | 0.0961 | N/A | 0.143 |
| MHHNU32266 | Unknown | 2.680e+03 | 0.676 | N/A | 0.209 |
| MHHNU31689 | Unknown | 7.960e+03 | 0.893 | N/A | 0.438 |
| MHHNU32362 | Unknown | 2.850e+03 | 0.675 | N/A | 0.217 |
| MHHNU32359 | Unknown | 5.160e+03 | 0.642 | N/A | 0.317 |
| MHHNU32195 | Unknown | 4.950e+03 | 0.636 | N/A | 0.308 |
| MHHNU32351 | Unknown | 3.870e+03 | 0.632 | N/A | 0.261 |
| MHHNU33070 | Unknown | 5.240e+04 | 0.897 | N/A | 2.36 |
| MHHNU32362 | Unknown | 5.790e+04 | 0.893 | N/A | 2.60 |

| \| COO Std 1 ng_mL \| \| \| \| --- \| --- \| --- \| \|  \| \| \| \| RT (Exp. RT): \| 0.865 (0.865) min \| \| \| Calculated Conc: \| 1.13 ng/mL \| \| \| Area: \| 2.40e+004 \|  \| \| Sample Type: \| (Standard) \| \| | 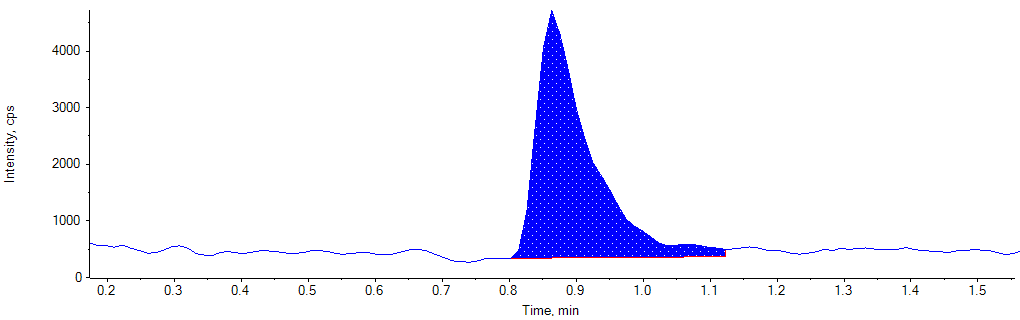 |
| --- | --- | --- | --- | --- | --- | --- | --- | --- | --- | --- | --- | --- | --- | --- | --- | --- | --- | --- | --- |

| \| COO Std 2 ng_mL \| \| \| \| --- \| --- \| --- \| \|  \| \| \| \| RT (Exp. RT): \| 0.868 (0.865) min \| \| \| Calculated Conc: \| 1.66 ng/mL \| \| \| Area: \| 3.63e+004 \|  \| \| Sample Type: \| (Standard) \| \| | 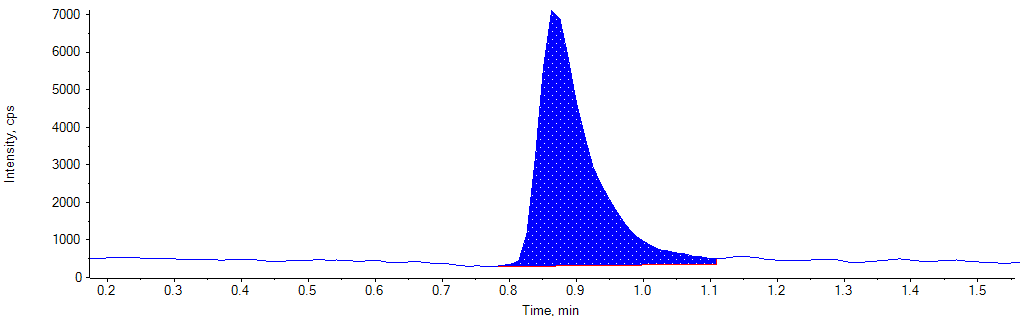 |
| --- | --- | --- | --- | --- | --- | --- | --- | --- | --- | --- | --- | --- | --- | --- | --- | --- | --- | --- | --- |

| \| COO Std 5 ng_mL \| \| \| \| --- \| --- \| --- \| \|  \| \| \| \| RT (Exp. RT): \| 0.865 (0.865) min \| \| \| Calculated Conc: \| 5.12 ng/mL \| \| \| Area: \| 1.16e+005 \|  \| \| Sample Type: \| (Standard) \| \| | 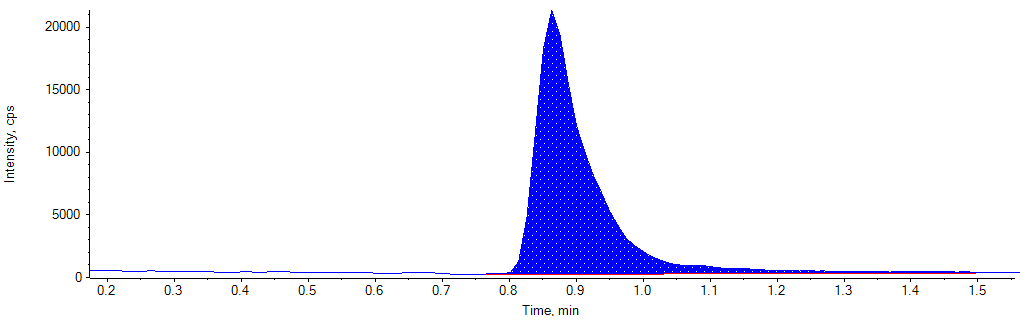 |
| --- | --- | --- | --- | --- | --- | --- | --- | --- | --- | --- | --- | --- | --- | --- | --- | --- | --- | --- | --- |

| \| COO Std 10 ng_mL \| \| \| \| --- \| --- \| --- \| \|  \| \| \| \| RT (Exp. RT): \| 0.867 (0.865) min \| \| \| Calculated Conc: \| 10.2 ng/mL \| \| \| Area: \| 2.32e+005 \|  \| \| Sample Type: \| (Standard) \| \| | 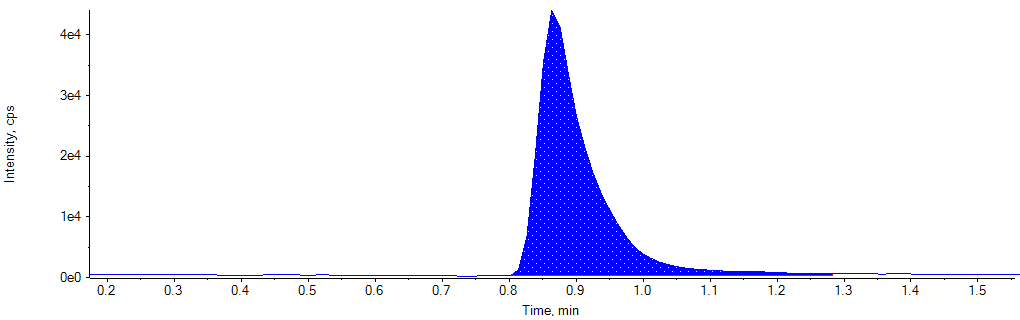 |
| --- | --- | --- | --- | --- | --- | --- | --- | --- | --- | --- | --- | --- | --- | --- | --- | --- | --- | --- | --- |

| \| COO Std 20 ng_mL \| \| \| \| --- \| --- \| --- \| \|  \| \| \| \| RT (Exp. RT): \| 0.865 (0.865) min \| \| \| Calculated Conc: \| 19.9 ng/mL \| \| \| Area: \| 4.58e+005 \|  \| \| Sample Type: \| (Standard) \| \| | 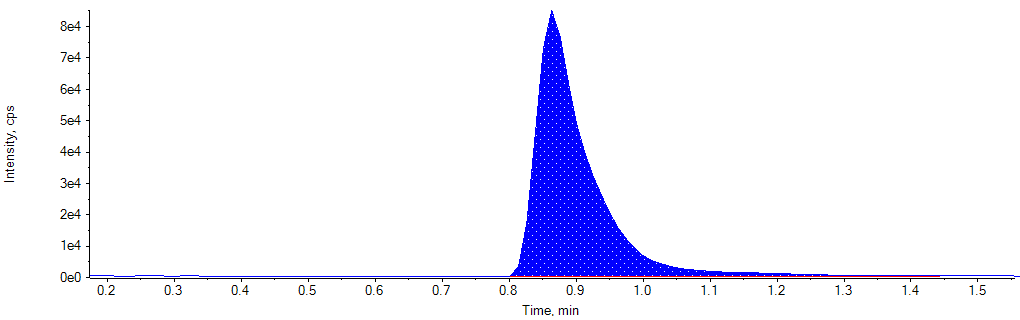 |
| --- | --- | --- | --- | --- | --- | --- | --- | --- | --- | --- | --- | --- | --- | --- | --- | --- | --- | --- | --- |

| \| Solvent blank \| \| \| \| --- \| --- \| --- \| \|  \| \| \| \| RT (Exp. RT): \| 0.666 (0.865) min \| \| \| Calculated Conc: \| 0.136 ng/mL \| \| \| Area: \| 9.77e+002 \|  \| \| Sample Type: \| (Unknown) \| \| | 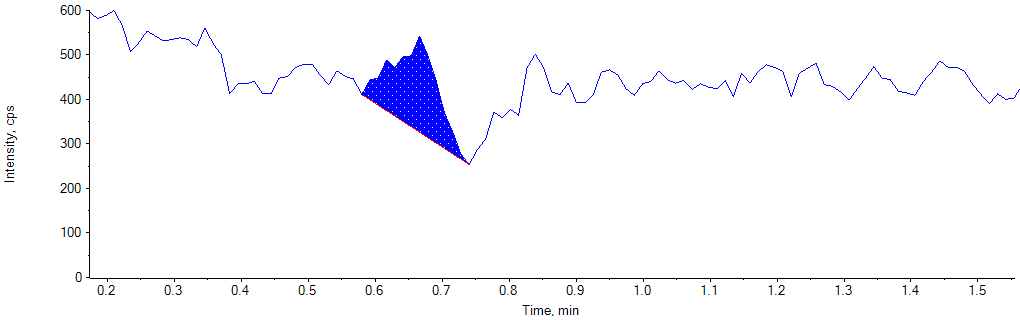 |
| --- | --- | --- | --- | --- | --- | --- | --- | --- | --- | --- | --- | --- | --- | --- | --- | --- | --- | --- | --- |

| \| MHHNU32337 \| \| \| \| --- \| --- \| --- \| \|  \| \| \| \| RT (Exp. RT): \| 0.0961 (0.00) min \| \| \| Calculated Conc: \| 0.143 ng/mL \| \| \| Area: \| 1.15e+003 \|  \| \| Sample Type: \| (Unknown) \| \| | 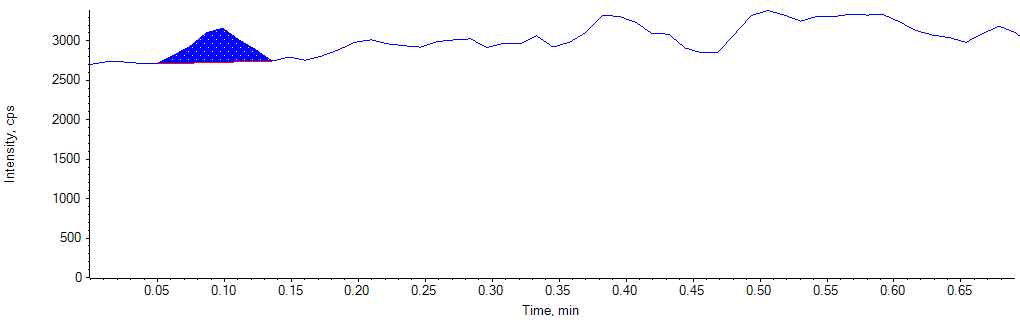 |
| --- | --- | --- | --- | --- | --- | --- | --- | --- | --- | --- | --- | --- | --- | --- | --- | --- | --- | --- | --- |

| \| MHHNU32266 \| \| \| \| --- \| --- \| --- \| \|  \| \| \| \| RT (Exp. RT): \| 0.676 (0.865) min \| \| \| Calculated Conc: \| 0.209 ng/mL \| \| \| Area: \| 2.68e+003 \|  \| \| Sample Type: \| (Unknown) \| \| | 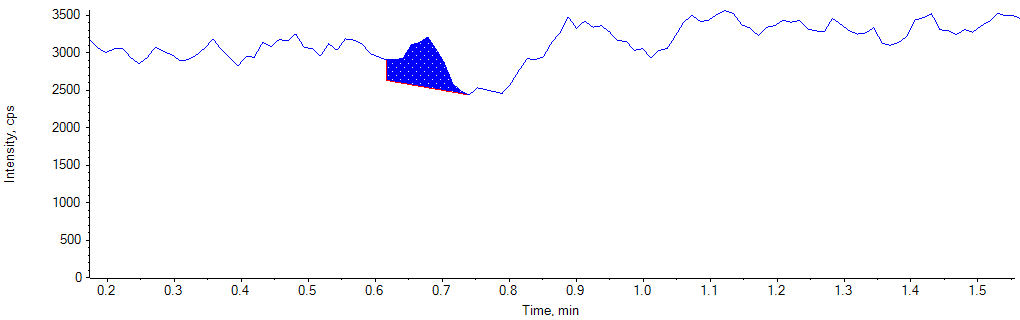 |
| --- | --- | --- | --- | --- | --- | --- | --- | --- | --- | --- | --- | --- | --- | --- | --- | --- | --- | --- | --- |

| \| MHHNU31689 \| \| \| \| --- \| --- \| --- \| \|  \| \| \| \| RT (Exp. RT): \| 0.893 (0.865) min \| \| \| Calculated Conc: \| 0.438 ng/mL \| \| \| Area: \| 7.96e+003 \|  \| \| Sample Type: \| (Unknown) \| \| | 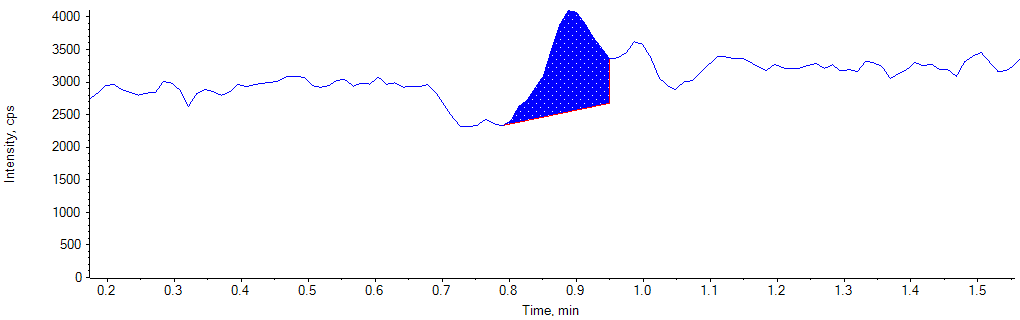 |
| --- | --- | --- | --- | --- | --- | --- | --- | --- | --- | --- | --- | --- | --- | --- | --- | --- | --- | --- | --- |

| \| MHHNU32362 \| \| \| \| --- \| --- \| --- \| \|  \| \| \| \| RT (Exp. RT): \| 0.675 (0.865) min \| \| \| Calculated Conc: \| 0.217 ng/mL \| \| \| Area: \| 2.85e+003 \|  \| \| Sample Type: \| (Unknown) \| \| | 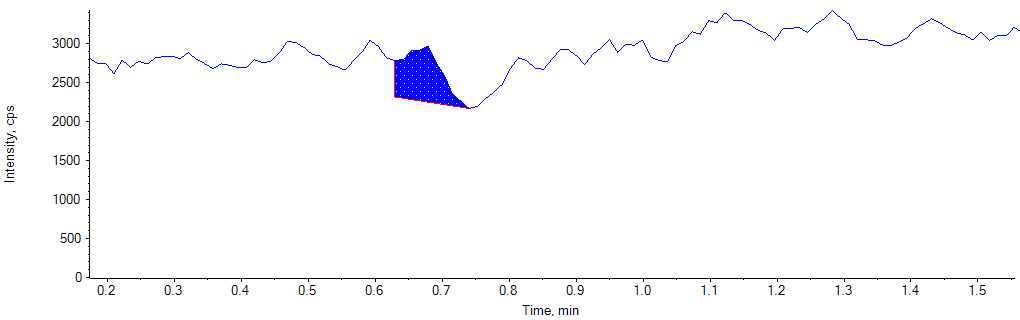 |
| --- | --- | --- | --- | --- | --- | --- | --- | --- | --- | --- | --- | --- | --- | --- | --- | --- | --- | --- | --- |

| \| MHHNU32359 \| \| \| \| --- \| --- \| --- \| \|  \| \| \| \| RT (Exp. RT): \| 0.642 (0.865) min \| \| \| Calculated Conc: \| 0.317 ng/mL \| \| \| Area: \| 5.16e+003 \|  \| \| Sample Type: \| (Unknown) \| \| | 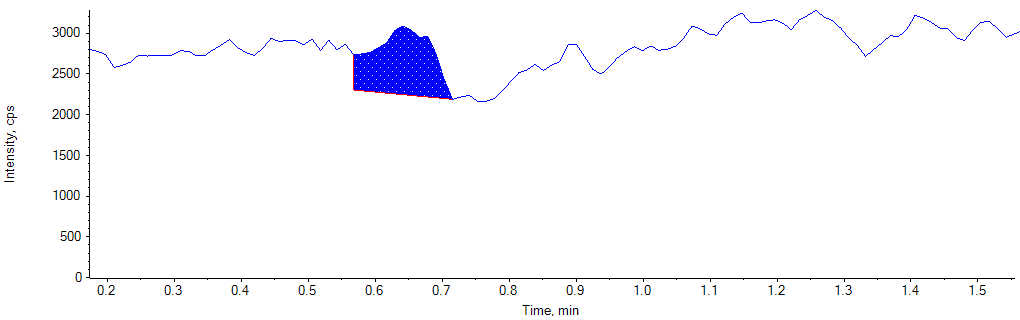 |
| --- | --- | --- | --- | --- | --- | --- | --- | --- | --- | --- | --- | --- | --- | --- | --- | --- | --- | --- | --- |

| \| MHHNU32195 \| \| \| \| --- \| --- \| --- \| \|  \| \| \| \| RT (Exp. RT): \| 0.636 (0.865) min \| \| \| Calculated Conc: \| 0.308 ng/mL \| \| \| Area: \| 4.95e+003 \|  \| \| Sample Type: \| (Unknown) \| \| | 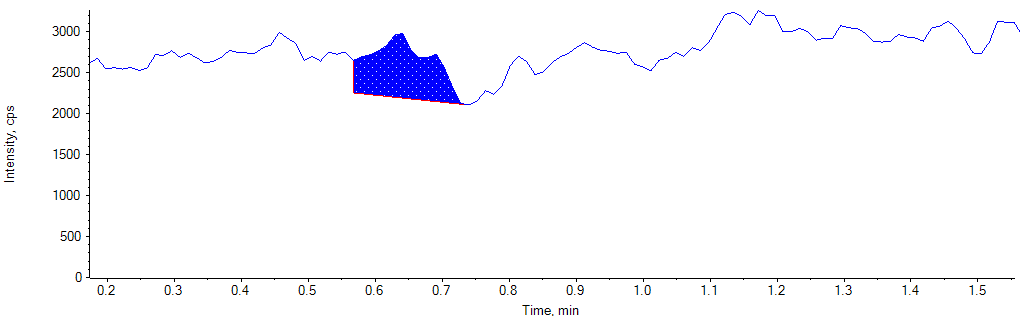 |
| --- | --- | --- | --- | --- | --- | --- | --- | --- | --- | --- | --- | --- | --- | --- | --- | --- | --- | --- | --- |

| \| MHHNU32351 \| \| \| \| --- \| --- \| --- \| \|  \| \| \| \| RT (Exp. RT): \| 0.632 (0.865) min \| \| \| Calculated Conc: \| 0.261 ng/mL \| \| \| Area: \| 3.87e+003 \|  \| \| Sample Type: \| (Unknown) \| \| | 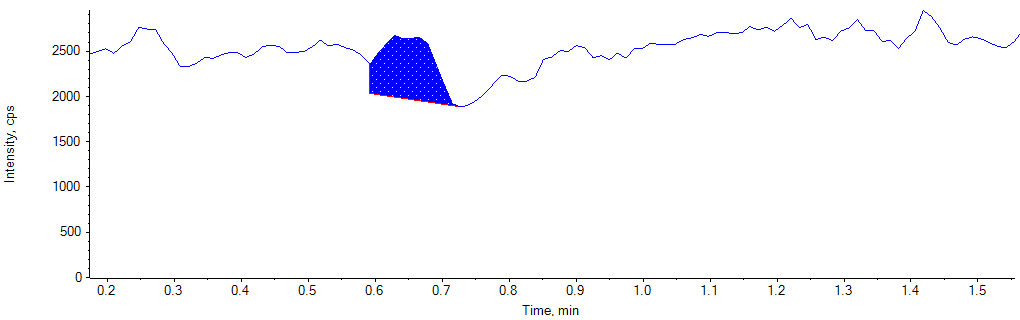 |
| --- | --- | --- | --- | --- | --- | --- | --- | --- | --- | --- | --- | --- | --- | --- | --- | --- | --- | --- | --- |

| \| MHHNU33070 \| \| \| \| --- \| --- \| --- \| \|  \| \| \| \| RT (Exp. RT): \| 0.897 (0.865) min \| \| \| Calculated Conc: \| 2.36 ng/mL \| \| \| Area: \| 5.24e+004 \|  \| \| Sample Type: \| (Unknown) \| \| | 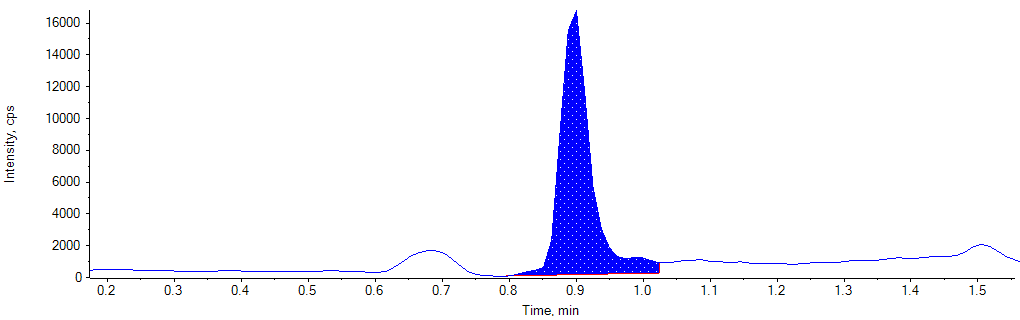 |
| --- | --- | --- | --- | --- | --- | --- | --- | --- | --- | --- | --- | --- | --- | --- | --- | --- | --- | --- | --- |

| \| MHHNU32362 \| \| \| \| --- \| --- \| --- \| \|  \| \| \| \| RT (Exp. RT): \| 0.893 (0.865) min \| \| \| Calculated Conc: \| 2.60 ng/mL \| \| \| Area: \| 5.79e+004 \|  \| \| Sample Type: \| (Unknown) \| \| | 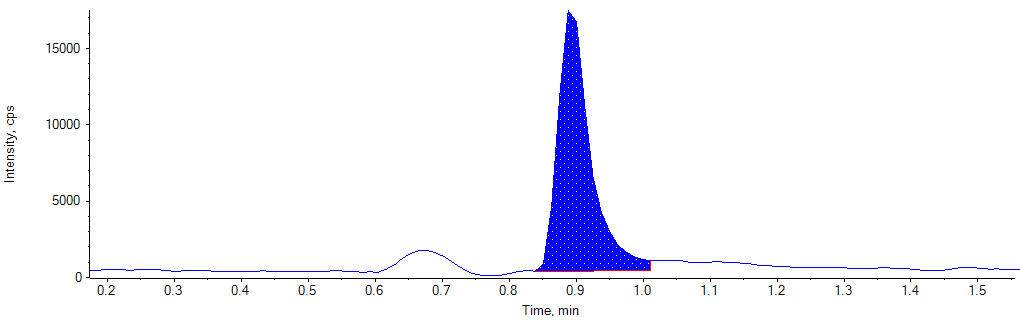 |
| --- | --- | --- | --- | --- | --- | --- | --- | --- | --- | --- | --- | --- | --- | --- | --- | --- | --- | --- | --- |

#### **Analyte Name:** ibotenic acid

#### Internal Standard: *No data for IS Peak Name*

####

| Data File | DataHunanshifandaxue 20210112.wiff | Result Table | Hunanshifandaxue 20220819-1.rdb |
| --- | --- | --- | --- |
| Acquisition Date | 1/14/2022 1:16:48 PM | Algorithm Used | MQL |
| Acquisition Method | 20180927-1.dam | Instrument Name | 4000 Q TRAP |
| Project | Muscarine and muscimol |  |  |

#### Regression Equation: y = 2.31e+004 x + -2.15e+003 (r = 0.9984)

| Expected Concentration | Number of Values | MeanCalculated Concentration | % Accuracy | Std. Deviation | %CV | |
| --- | --- | --- | --- | --- | --- | --- |
| 1 | 1 | 1.13 | 113.2 | NaN | | NaN |
| 2 | 1 | 1.66 | 83.2 | NaN | | NaN |
| 5 | 1 | 5.12 | 102.4 | NaN | | NaN |
| 10 | 1 | 10.15 | 101.5 | NaN | | NaN |
| 20 | 1 | 19.93 | 99.6 | NaN | | NaN |


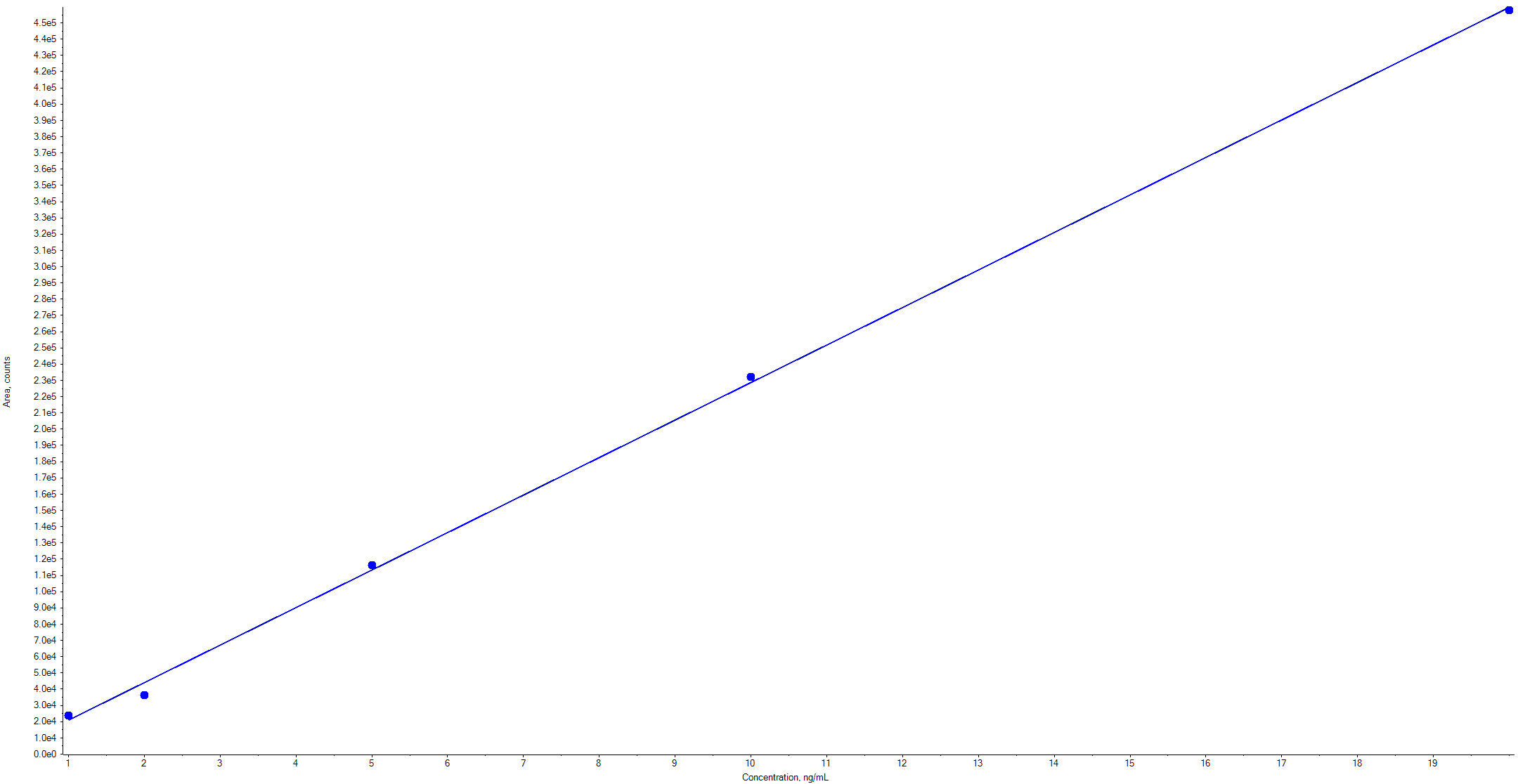


#### **Analyte:** Psilocybin **(**285.200/205.300 Da**)**

| Data File | DataStd 20220822.wiff | Result Table | Hunanshifandaxue 20220822.rdb |
| --- | --- | --- | --- |
| Acquisition Date | 1/16/2022 8:31:37 AM | Algorithm Used | MQL |
| Acquisition Method | Psilocybin 20191022.dam | Instrument Name | 4000 Q TRAP |
| Project | Psilocybin |  |  |

| Sample Name | Sample Type | Area (cps) | RT (min) | Target [Conc]. (ng/mL) | Calculated Conc. (ng/mL) |
| --- | --- | --- | --- | --- | --- |
| Std 2 ng_mL | Standard | 1.110e+03 | 1.27 | 2.00 | 2.97 |
| Std 5 ng_mL | Standard | 2.170e+03 | 1.25 | 5.00 | 4.15 |
| Std 10 ng_mL | Standard | 5.110e+03 | 1.22 | 10.0 | 7.41 |
| Std 20 ng_mL | Standard | 1.330e+04 | 1.21 | 20.0 | 16.5 |
| Std 60 ng_mL | Standard | 4.890e+04 | 1.25 | 60.0 | 56.0 |
| Solvent blank | Unknown | 1.560e+01 | 1.36 | N/A | N/A |
| MHHNU32337 | Unknown | 2.080e+01 | 1.25 | N/A | N/A |
| MHHNU33070 | Unknown | 3.330e+01 | 1.37 | N/A | N/A |
| MHHNU32266 | Unknown | 2.920e+01 | 1.04 | N/A | N/A |
| MHHNU32362 | Unknown | 1.870e+01 | 1.24 | N/A | N/A |
| MHHNU32359 | Unknown | 4.480e+01 | 1.03 | N/A | N/A |
| MHHNU32195 | Unknown | 1.040e+01 | 1.39 | N/A | N/A |
| MHHNU32162 | Unknown | 4.170e+01 | 1.01 | N/A | N/A |
| MHHNU32351 | Unknown | 4.170e+01 | 1.09 | N/A | N/A |
| MHHNU31689 | Unknown | 1.170e+02 | 1.04 | N/A | N/A |

| \| Std 2 ng_mL \| \| \| \| --- \| --- \| --- \| \|  \| \| \| \| RT (Exp. RT): \| 1.27 (0.00) min \| \| \| Calculated Conc: \| 2.97 ng/mL \| \| \| Area: \| 1.11e+003 \|  \| \| Sample Type: \| (Standard) \| \| | 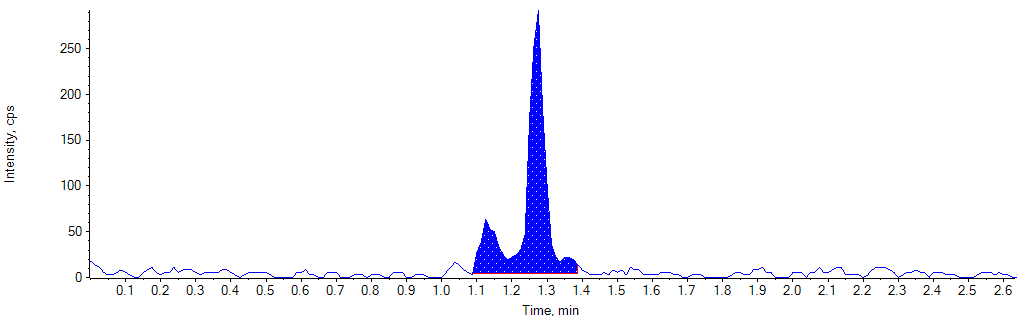 |
| --- | --- | --- | --- | --- | --- | --- | --- | --- | --- | --- | --- | --- | --- | --- | --- | --- | --- | --- | --- |

| \| Std 5 ng_mL \| \| \| \| --- \| --- \| --- \| \|  \| \| \| \| RT (Exp. RT): \| 1.25 (0.00) min \| \| \| Calculated Conc: \| 4.15 ng/mL \| \| \| Area: \| 2.17e+003 \|  \| \| Sample Type: \| (Standard) \| \| | 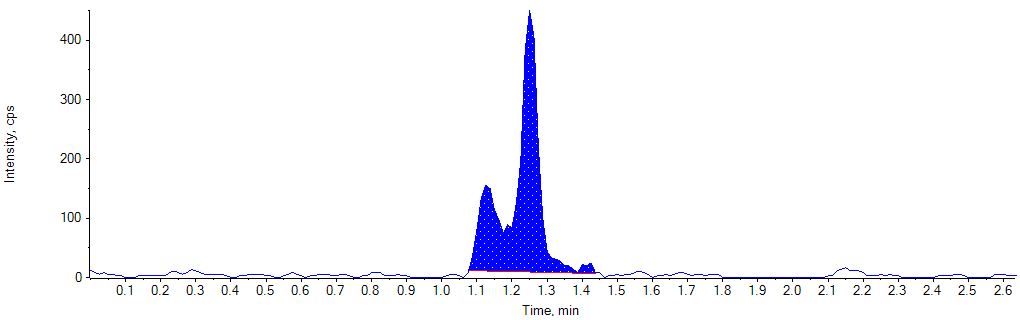 |
| --- | --- | --- | --- | --- | --- | --- | --- | --- | --- | --- | --- | --- | --- | --- | --- | --- | --- | --- | --- |

| \| Std 10 ng_mL \| \| \| \| --- \| --- \| --- \| \|  \| \| \| \| RT (Exp. RT): \| 1.22 (1.25) min \| \| \| Calculated Conc: \| 7.41 ng/mL \| \| \| Area: \| 5.11e+003 \|  \| \| Sample Type: \| (Standard) \| \| | 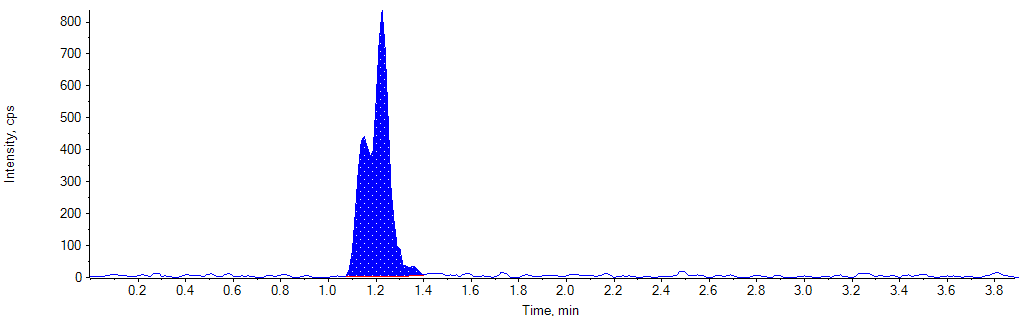 |
| --- | --- | --- | --- | --- | --- | --- | --- | --- | --- | --- | --- | --- | --- | --- | --- | --- | --- | --- | --- |

| \| Std 20 ng_mL \| \| \| \| --- \| --- \| --- \| \|  \| \| \| \| RT (Exp. RT): \| 1.21 (1.25) min \| \| \| Calculated Conc: \| 16.5 ng/mL \| \| \| Area: \| 1.33e+004 \|  \| \| Sample Type: \| (Standard) \| \| | 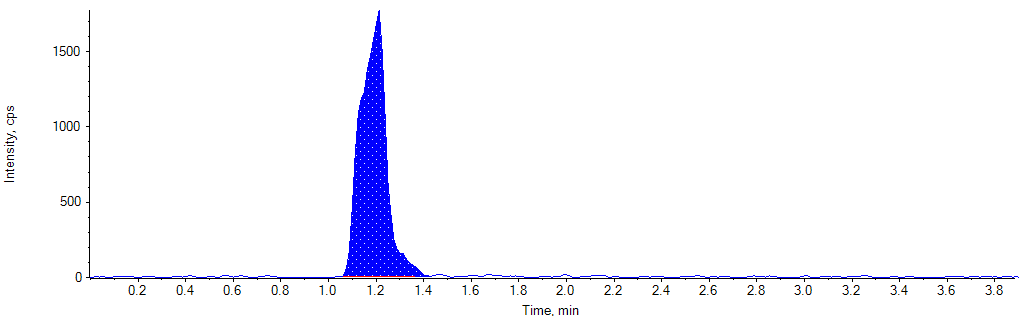 |
| --- | --- | --- | --- | --- | --- | --- | --- | --- | --- | --- | --- | --- | --- | --- | --- | --- | --- | --- | --- |

| \| Std 50 ng_mL \| \| \| \| --- \| --- \| --- \| \|  \| \| \| \| RT (Exp. RT): \| 1.15 (0.00) min \| \| \| Calculated Conc: \| 56.0 ng/mL \| \| \| Area: \| 4.89e+004 \|  \| \| Sample Type: \| (Standard) \| \| | 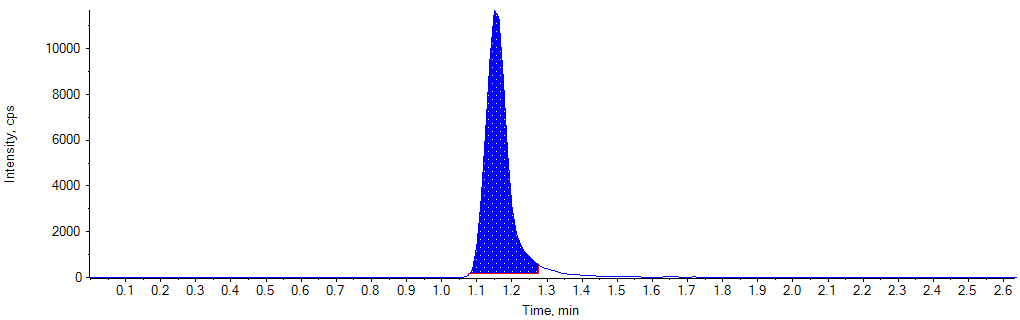 |
| --- | --- | --- | --- | --- | --- | --- | --- | --- | --- | --- | --- | --- | --- | --- | --- | --- | --- | --- | --- |

| \| Solvent blank \| \| \| \| --- \| --- \| --- \| \|  \| \| \| \| RT (Exp. RT): \| 1.36 (1.25) min \| \| \| Calculated Conc: \| 1.76 ng/mL \| \| \| Area: \| 1.56e+001 \|  \| \| Sample Type: \| (Unknown) \| \| | 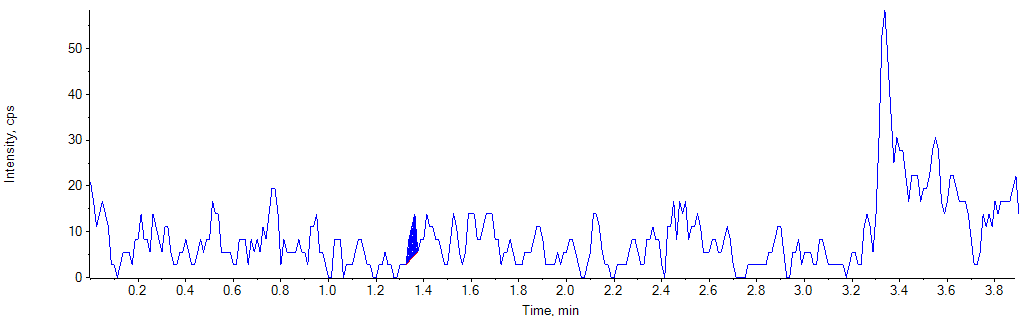 |
| --- | --- | --- | --- | --- | --- | --- | --- | --- | --- | --- | --- | --- | --- | --- | --- | --- | --- | --- | --- |

| \| MHHNU32337 \| \| \| \| --- \| --- \| --- \| \|  \| \| \| \| RT (Exp. RT): \| 1.25 (1.25) min \| \| \| Calculated Conc: \| 1.76 ng/mL \| \| \| Area: \| 2.08e+001 \|  \| \| Sample Type: \| (Unknown) \| \| | 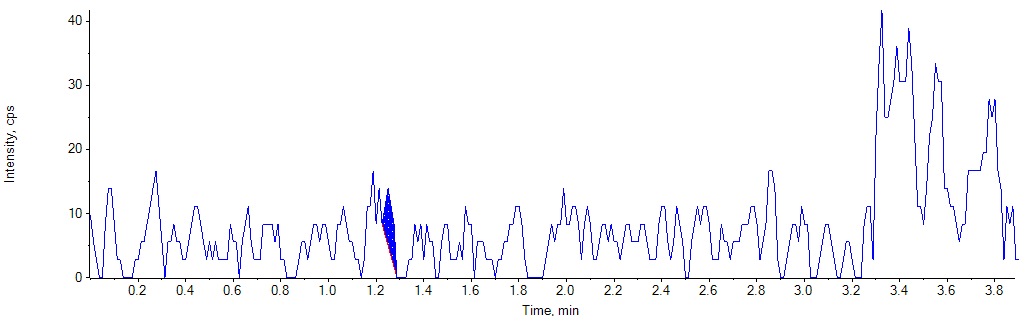 |
| --- | --- | --- | --- | --- | --- | --- | --- | --- | --- | --- | --- | --- | --- | --- | --- | --- | --- | --- | --- |

| \| MHHNU33070 \| \| \| \| --- \| --- \| --- \| \|  \| \| \| \| RT (Exp. RT): \| 1.37 (1.25) min \| \| \| Calculated Conc: \| 1.78 ng/mL \| \| \| Area: \| 3.33e+001 \|  \| \| Sample Type: \| (Unknown) \| \| | 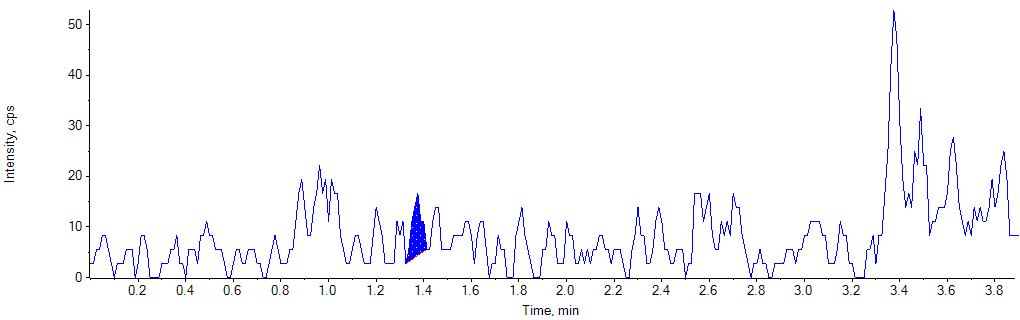 |
| --- | --- | --- | --- | --- | --- | --- | --- | --- | --- | --- | --- | --- | --- | --- | --- | --- | --- | --- | --- |

| \| MHHNU32266 \| \| \| \| --- \| --- \| --- \| \|  \| \| \| \| RT (Exp. RT): \| 1.04 (1.25) min \| \| \| Calculated Conc: \| 1.77 ng/mL \| \| \| Area: \| 2.92e+001 \|  \| \| Sample Type: \| (Unknown) \| \| | 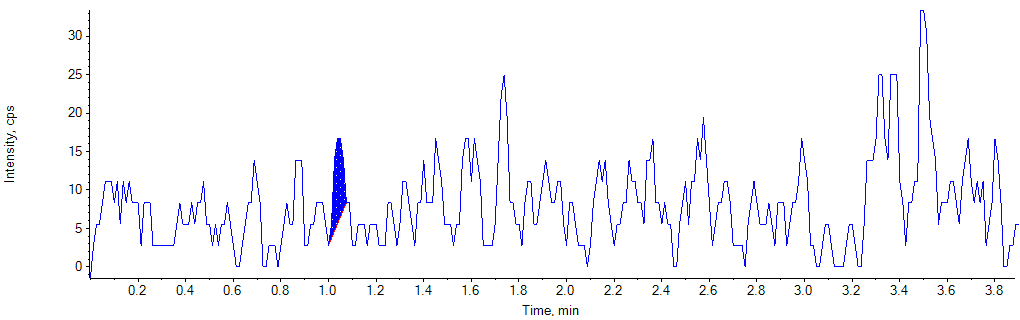 |
| --- | --- | --- | --- | --- | --- | --- | --- | --- | --- | --- | --- | --- | --- | --- | --- | --- | --- | --- | --- |

| \| MHHNU32362 \| \| \| \| --- \| --- \| --- \| \|  \| \| \| \| RT (Exp. RT): \| 1.24 (1.25) min \| \| \| Calculated Conc: \| 1.76 ng/mL \| \| \| Area: \| 1.87e+001 \|  \| \| Sample Type: \| (Unknown) \| \| | 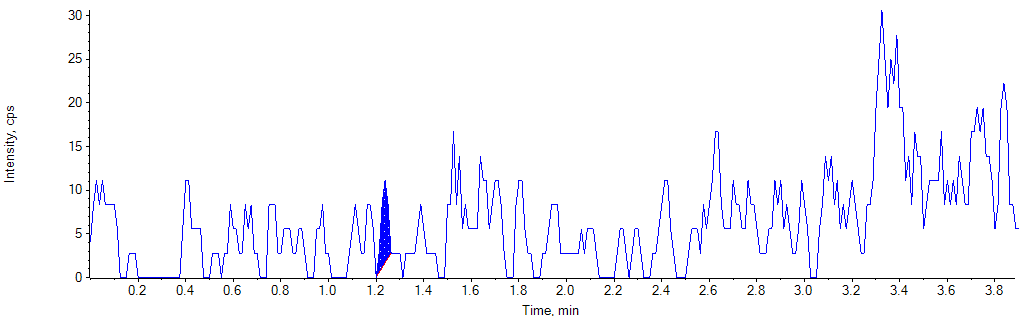 |
| --- | --- | --- | --- | --- | --- | --- | --- | --- | --- | --- | --- | --- | --- | --- | --- | --- | --- | --- | --- |

| \| MHHNU32359 \| \| \| \| --- \| --- \| --- \| \|  \| \| \| \| RT (Exp. RT): \| 1.03 (1.25) min \| \| \| Calculated Conc: \| 1.79 ng/mL \| \| \| Area: \| 4.48e+001 \|  \| \| Sample Type: \| (Unknown) \| \| | 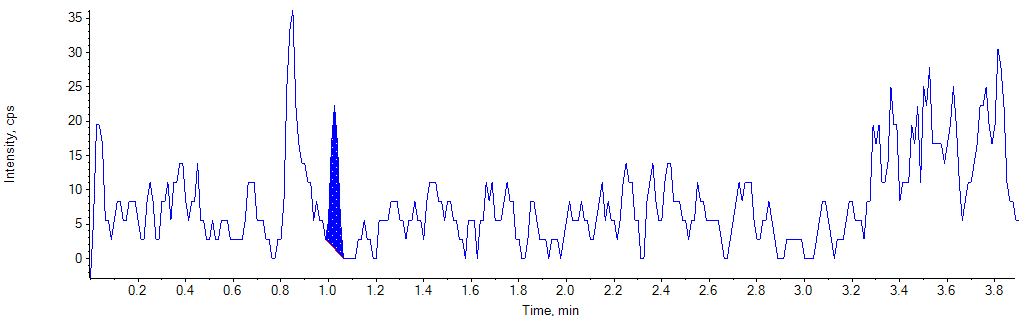 |
| --- | --- | --- | --- | --- | --- | --- | --- | --- | --- | --- | --- | --- | --- | --- | --- | --- | --- | --- | --- |

| \| MHHNU32195 \| \| \| \| --- \| --- \| --- \| \|  \| \| \| \| RT (Exp. RT): \| 1.39 (1.25) min \| \| \| Calculated Conc: \| 1.75 ng/mL \| \| \| Area: \| 1.04e+001 \|  \| \| Sample Type: \| (Unknown) \| \| | 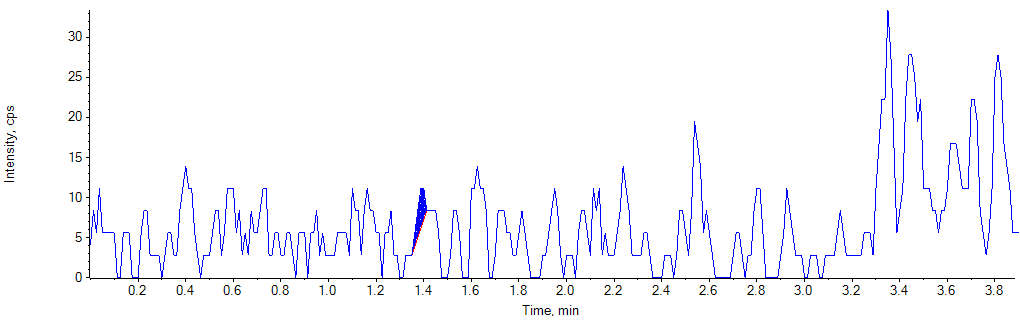 |
| --- | --- | --- | --- | --- | --- | --- | --- | --- | --- | --- | --- | --- | --- | --- | --- | --- | --- | --- | --- |

| \| MHHNU32162 \| \| \| \| --- \| --- \| --- \| \|  \| \| \| \| RT (Exp. RT): \| 1.01 (1.25) min \| \| \| Calculated Conc: \| 1.79 ng/mL \| \| \| Area: \| 4.17e+001 \|  \| \| Sample Type: \| (Unknown) \| \| | 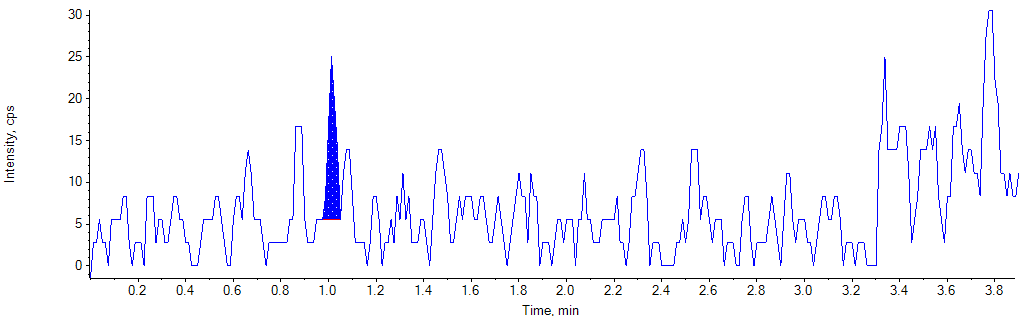 |
| --- | --- | --- | --- | --- | --- | --- | --- | --- | --- | --- | --- | --- | --- | --- | --- | --- | --- | --- | --- |

| \| MHHNU32351 \| \| \| \| --- \| --- \| --- \| \|  \| \| \| \| RT (Exp. RT): \| 1.09 (1.25) min \| \| \| Calculated Conc: \| 1.79 ng/mL \| \| \| Area: \| 4.17e+001 \|  \| \| Sample Type: \| (Unknown) \| \| | 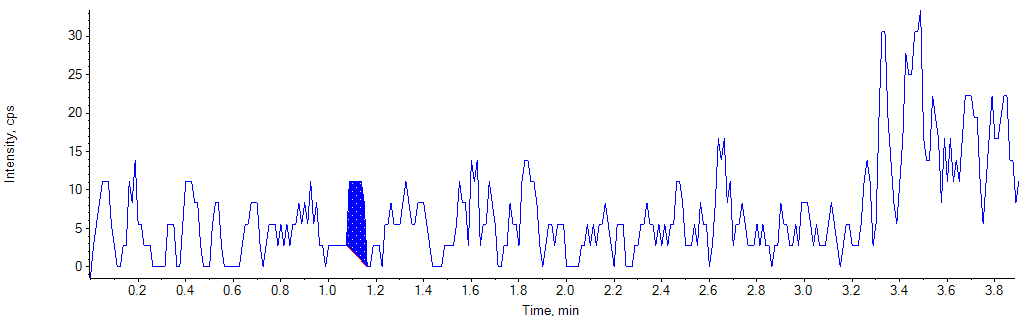 |
| --- | --- | --- | --- | --- | --- | --- | --- | --- | --- | --- | --- | --- | --- | --- | --- | --- | --- | --- | --- |

| \| MHHNU31689 \| \| \| \| --- \| --- \| --- \| \|  \| \| \| \| RT (Exp. RT): \| 1.04 (1.25) min \| \| \| Calculated Conc: \| 1.87 ng/mL \| \| \| Area: \| 1.17e+002 \|  \| \| Sample Type: \| (Unknown) \| \| | 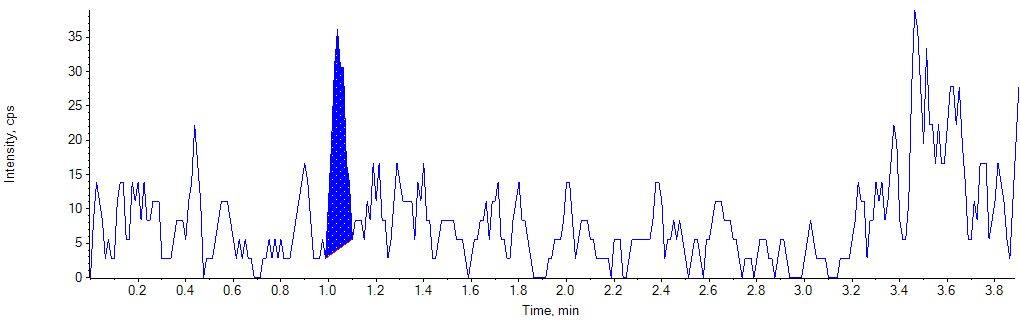 |
| --- | --- | --- | --- | --- | --- | --- | --- | --- | --- | --- | --- | --- | --- | --- | --- | --- | --- | --- | --- |

####

#### **Analyte Name:** Psilocybin

#### Internal Standard: *No data for IS Peak Name*

####

| Data File | DataStd 20220822.wiff | Result Table | Hunanshifandaxue 20220822.rdb |
| --- | --- | --- | --- |
| Acquisition Date | 1/16/2022 8:31:37 AM | Algorithm Used | MQL |
| Acquisition Method | Psilocybin 20191022.dam | Instrument Name | 4000 Q TRAP |
| Project | Psilocybin |  |  |

#### Regression Equation: y = 783 x + -1.08e+003 (r = 0.9907)

| Expected Concentration | Number of Values | MeanCalculated Concentration | % Accuracy | Std. Deviation | %CV | |
| --- | --- | --- | --- | --- | --- | --- |
| 2 | 1 | 2.80 | 139.8 | NaN | | NaN |
| 5 | 1 | 4.16 | 83.2 | NaN | | NaN |
| 10 | 1 | 7.91 | 79.1 | NaN | | NaN |
| 20 | 1 | 18.31 | 91.6 | NaN | | NaN |
| 60 | 1 | 63.82 | 106.4 | NaN | | NaN |


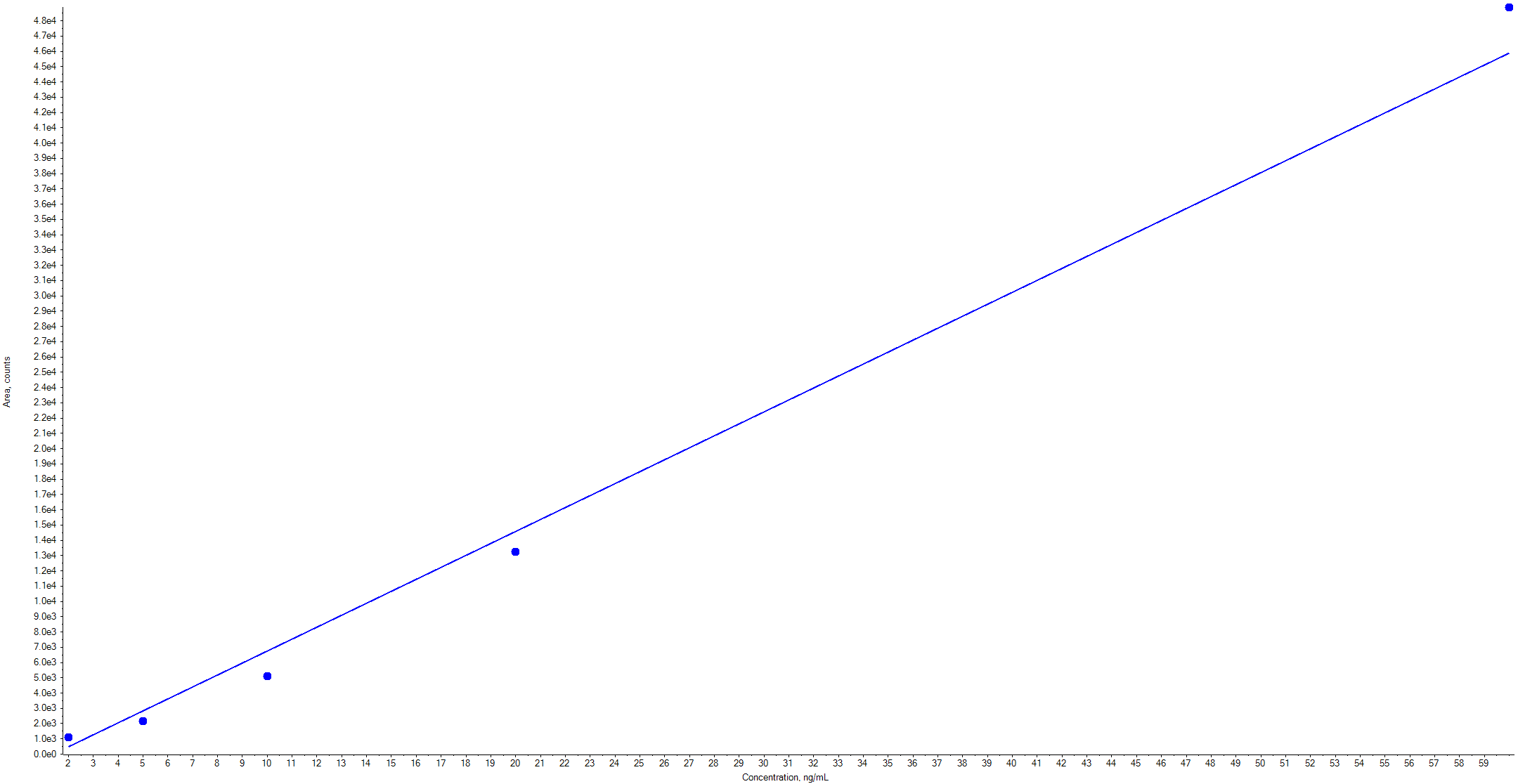

Supplement: Supplementary Data Sheet 1 — Combined sequences (ITS, nrLSU, and rpb2) alignment dataset. [file Data_Sheet_2.docx]
